# A Long Fragment Aligner called ALFALFA

**DOI:** 10.1186/s12859-015-0533-0

**Published:** 2015-05-15

**Authors:** Michaël Vyverman, Bernard De Baets, Veerle Fack, Peter Dawyndt

**Affiliations:** Department of Applied Mathematics, Computer Science and Statistics, Ghent University, Krijgslaan 281 Building S9, Ghent, B-9000 Belgium; Department of Mathematical Modelling, Statistics and Bioinformatics, Ghent University, Coupure links 653, Ghent, B-9000 Belgium

**Keywords:** Long read mapping, Enhanced sparse suffix array, Super-maximal exact matches, Paired-end reads

## Abstract

**Background:**

Rapid evolutions in sequencing technology force read mappers into flexible adaptation to longer reads, changing error models, memory barriers and novel applications.

**Results:**

ALFALFA achieves a high performance in accurately mapping long single-end and paired-end reads to gigabase-scale reference genomes, while remaining competitive for mapping shorter reads. Its seed-and-extend workflow is underpinned by fast retrieval of super-maximal exact matches from an enhanced sparse suffix array, with flexible parameter tuning to balance performance, memory footprint and accuracy.

**Conclusions:**

ALFALFA is open source and available at http://alfalfa.ugent.be.

**Electronic supplementary material:**

The online version of this article (doi:10.1186/s12859-015-0533-0) contains supplementary material, which is available to authorized users.

## Background

Bioinformatics research is currently dominated by the (r)evolution in high-throughput sequencing technology. New sequencing platforms produce biological sequence fragments faster and cheaper than ever before. The resulting growth in access to large amounts of data opens perspectives for new applications and prestigious projects, but simultaneously pushes existing sequence analysis tools beyond their limits as data storage, computational analysis and interpretation become true bottlenecks in life sciences research.

Mapping sequencing reads to reference genomes plays a key role in many genomics analysis pipelines. The olympic motto *citius*, *altius*, *fortius* in the context of this computationally intensive problem drives read mappers into the algorithmically challenging quest to find an optimal balance between maximal speed, minimal memory footprint and maximal accuracy. Read mappers are also expected to shoot at a moving target, as reads produced by fast evolving technologies differ in length distribution and sequencing errors. Read mappers and their underlying index structures are therefore under constant development to handle specific applications or data models and to further improve implementations [1,2].

Although design and implementation of existing read mappers differ in their choice of algorithmic techniques, optimizations and heuristics, they share many of their key concepts and follow a common general outline. A preprocessing step of indexing reference genomes and/or sequencing reads must guarantee fast substring matching. The overall search space is pruned to candidate genomic regions by searching matching segments (called seeds) between reads and the reference genome. These candidate regions are then further investigated to look for acceptable alignments that reach a particular score.

Recent advances in next-generation sequencing technologies have led to increased read lengths, higher error rates and error models showing more and longer indels. This general trend is likely to continue with third-generation sequencing technologies like Oxford Nanopore and Pacific Biosciences [3]. Most of the current read mappers target short reads and allow for no or low numbers of mismatches and/or indels. This makes them vulnerable to the ongoing technological advances. It has inspired a second generation of novel read mappers (GEM [4]), while authors of short read mappers present new versions equipped for aligning longer reads with higher error rates (Bowtie 2 [5], BWA-SW [6], BWA-MEM [7] and CUSHAW3 [8]). Recurring strategies include increasing the seed lengths, clustering neighboring seeds into candidate regions and optimizing the implementations of global and local alignment algorithms using banded and bit-parallel versions. However, except for BWA-SW and BWA-MEM, none of the existing mappers scales well for read lengths up to several kilobases.

**Figure 1:**
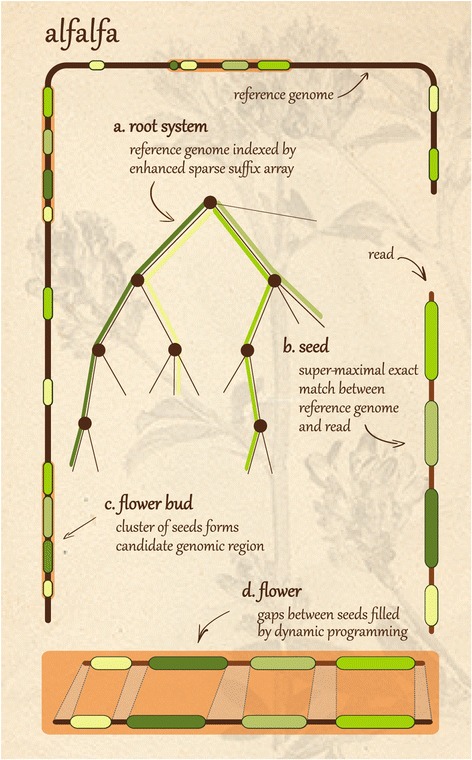
Algorithmic workflow of ALFALFA. ALFALFA follows a canonical seed-and-extend workflow for mapping reads onto a reference genome. The reference genome is indexed by an enhanced sparse suffix array **(a)** to enable quick retrieval of variable-length seeds called super-maximal exact matches between a read and the reference genome **(b)**. Seeds are then grouped into non-overlapping clusters that mark candidate genomic regions for read alignment **(c)**. Handling of candidate regions is prioritized by agglomerate base pair coverage of the seeds. The final extend phase samples seeds from candidate regions to form collinear chains that are bridged using banded dynamic programming **(d)**. All of these steps strive to make optimal reuse of seeds in order to avoid superfluous computations. Background image used with permission from Walter Obermayer.

The read mapper ALFALFA presented in this paper is extremely fast and accurate at mapping long reads (>500 bp), while still being competitive for moderately sized reads (>100 bp). Its implementation of the canonical seed-and-extend approach (Figure 1) is empowered by a novel index structure, combined with several new optimizations and heuristics. Both end-to-end and local read alignment are supported, and several strategies for paired-end mapping can efficiently handle large variations in insert size. ALFALFA is unique in using enhanced sparse suffix arrays to index reference genomes. This data structure facilitates fast calculation of maximal and super-maximal exact matches [9] and supports the important design goal of balancing between processing speed, memory consumption and mapping accuracy. The speed-memory trade-off is tuned by setting the sparseness value of the index. The techniques and heuristics used to filter and combine seeds and candidate regions are designed to handle longer reads. Furthermore, ALFALFA uses a chaining algorithm to speed up dynamic programming extension of candidate regions.

## Implementation

Due to huge differences in size between sequencing reads and reference genomes, most read mappers share a high-level strategy of *i*) finding matching segments that are used to *ii*) prune the search space to genomic regions in which *iii*) alignments are found that meet a particular scoring threshold. These steps are usually preceded by a step in which either the reference genome or read data set is indexed. Notwithstanding this common search strategy, read mappers differ in many of their design choices (from seed type, over index structure to scoring function used for accepting alignments) and in a multitude of optimizations and heuristics used during all search phases. These algorithmic choices do not only govern the trade-off between performance, memory usage and mapping accuracy, but are also geared towards particular sequencing technologies or specific types of applications. Best-mappers such as Bowtie [10] and BWA [11] search for a single optimal alignment according to a particular scoring function, whereas all-mappers such as RazerS3 [12] and GEM focus on finding all alignments within a given Hamming or Levenshtein distance.

Next-generation and third-generation sequencing technologies produce ever longer reads with varying degrees and types of sequencing errors. This evolution has a serious impact on the design of read mappers, as can be seen from the wide range of software packages that have been proposed over the last years. Longer reads feature the possibility of more and/or longer seeds. This opens perspectives for reducing the number of candidate regions along the reference genome that need further investigation, but at the same time increases their size.

The ALFALFA algorithm is outlined in Figure 1. ALFALFA takes advantage of the technological evolution towards longer reads by using maximal exact matches [MEMs] [13,14] and super-maximal exact matches [SMEMs] [7] as seeds (Figure 1 step *b*). These seeds are then extensively filtered and triaged to allow for more accurate prioritization of candidate regions (Figure 1 step *c*). To further limit the number of expensive dynamic programming computations needed, ALFALFA chains seeds together to form a gapped alignment. As a result, the extension phase is limited to filling gaps in between chains while evaluating alignment quality (Figure 1 step *d*). The following sections discuss the ALFALFA workflow in more detail.

### Enhanced sparse suffix arrays

To boost seed-finding, read mappers rely on fast and low memory-footprint index structures such as *k*-mer lookup tables and FM-indexes [1]. ALFALFA is the first read mapper that makes use of an enhanced sparse suffix array [ESSA] index structure (Figure 1 step *a*). Instead of indexing all suffixes of the reference genome into a suffix array, sparse suffix arrays reduce memory consumption by sparsely sampling the list of suffixes, this in contrast to compressed suffix arrays and FM-indexes, which instead store a sparse sample of suffix array values. Sparse suffix arrays can be further enhanced with auxiliary data structures to provide fast string matching [9], similar to the way Burrows-Wheeler transformed texts are enhanced with auxiliary data structures to form FM-indexes. The sparseness value *s* of sparse suffix arrays (controlled by the option -s) provides an easily tunable trade-off to balance performance and memory footprint. In theory, sparse suffix arrays take up 9/*s*+1 bytes of memory per indexed base. A sparse suffix array with sparseness factor 12 thus indexes the entire human genome with a memory footprint of 5.8 GB. This is similar in size to the memory consumed by some of the FM-indexes used by other read mappers (Table 1). Earlier results have shown that enhanced sparse suffix arrays are competitive in MEM-finding when compared to implementations using an FM-index [9]. They especially perform extremely well in cases where the number of seeds is high, a likely scenario when mapping long reads.

**Table 1:** **Comparison of the used index structures and memory requirements of evaluated mappers**

**Mapper**	**Index type**	**Adjustable**	**Size on disk**	**Peak memory**	**Construction time**
			**(GB)**	**(GB)**	**(h:mm)**
ALFALFA (*s*=4)	ESSA	yes	10.7	11.0	0:19
ALFALFA (*s*=12)	ESSA	yes	5.6	5.7	0:10
Bowtie 2	FM-index	yes	3.9	5.3	1:58
BWA	FM-index	no	5.2	5.4	1:18
CUSHAW3	FM-index	no	3.7	3.5	0:36
GEM	FM-index	yes	4.8	5.0	N.A. ^*§*^

The ALFALFA index is evaluated for two different sparseness values. The read mappers BWA-SW and BWA-MEM both use the same index structure, jointly reported as BWA. The third column indicates whether memory footprint can be tuned via user-specified options. The fourth column reports the memory footprint of the index structure when stored on disk. The fifth column provides peak memory of the read mapper observed during alignment of 1kbp reads. The time needed to construct the index structure is given in the last column. ^*§*^A pre-built GEM index was downloaded from the GEM website as the indexer of this mapper ran into a fatal error in our test environment.

### Seed-finding

Seed-finding is the first major phase in the mapping process. Depending on the data and parameter settings, it usually takes about a quarter to half of the total runtime. Ideally, seed-finding produces a limited number of long seeds that cover as much of the mapping location as possible. Finding too many seeds results in an exponential increase of candidate mapping locations and usually favors highly repetitive regions in the genome. As a result, mapping locations may be missed. Finding too few seeds results in a possible loss of good mapping locations and shorter chains. The latter may increase the computational cost of the extension phase.

**Figure 2:**
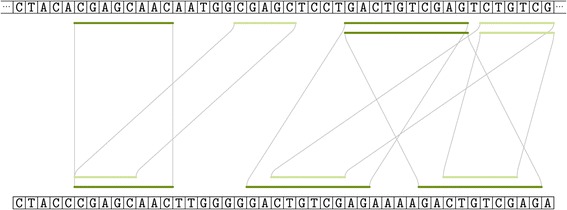
Example illustrating the difference between maximal and super-maximal exact matches. All maximal exact matches with minimal length four between a sequencing read (bottom) and a reference genome (top). Dark green lines represent pairs of intervals that are super-maximal exact matches. Light green lines represent pairs of intervals that are maximal exact matches but are not super-maximal.

ALFALFA tries to balance the number and the quality of seeds using a combination of maximal and super-maximal exact matches. The intervals [*i*..*i*+*ℓ*−1] and [*j*..*j*+*ℓ*−1] correspond to a maximal exact match between a read and a reference genome if there is a perfect match between both subsequences of length *ℓ* starting at position *i* in the read and at position *j* in the reference genome, with mismatches occurring at positions (*i*−1,*j*−1) and (*i*+*ℓ*,*j*+*ℓ*) just before and after the location of the matching subsequence. Since MEMs between a read and a reference genome may overlap, super-maximal exact matches are defined as MEMs that are not contained in another MEM in the read [15]. The difference between MEMs and SMEMs is shown in Figure 2. MEMs were introduced in one of our earlier proof-of-concept implementations [14] and CUSHAW2 [13], whereas SMEMs were introduced in BWA-MEM [7]. In comparison to fixed length seeds, MEMs and SMEMs have the advantage of potentially covering larger parts of a read. As such, they bear more information about the relevance of a region in which the seed is found. This information can be used to filter out candidate regions with low probability of finding good alignments.

One of the strongholds of ALFALFA is its use of the essaMEM algorithm [9] as a way to identify candidate regions for mapping reads to the reference genome. essaMEM locates MEMs and SMEMs using exact string matching between an enhanced sparse suffix array index structure and a subset of suffixes sampled from the read. ALFALFA automatically selects the sampling value based on the sparseness of the index and the minimum seed length (Supplementary Methods in Additional file 1). In addition, ALFALFA imposes a minimum seed length, which is automatically tuned using the read length and the expected number of differences in an alignment. Although the algorithm does not guarantee to find all SMEMs, the produced set of MEMs and SMEMs works well in practice. In case no seeds are found using using the initial parameter settings, a rescue procedure is initiated that gradually lowers restrictions until seeds are found. This procedure helps the algorithm to find suitable candidate regions for rare reads that contain excessive amounts of errors compared to the average of the read set.

### Candidate regions

A combination of neighboring seeds increases the evidence that some region in the reference genome holds potential to serve as a mapping location [5]. ALFALFA therefore sorts seeds according to their starting position in the reference genome and bins them into non-overlapping clusters using the locally longest seeds as anchors around which regions are built. This results in a list of candidate regions along the reference genome. To limit the number of candidate regions requiring further examination, only SMEMs and rare MEMs are used for candidate region identification (more details are provided in the candidate region identification section of the Supplementary Methods). Afterwards, MEMs overlapping with candidate regions are also taken into consideration to increase the number of read bases covered by seeds. This new optimization introduced by ALFALFA positively affects accuracy without a major performance overhead.

A successful candidate region extension results in one or more *feasible* alignments that show sufficient similarity between the read and the reference genome. Candidate regions are then ranked by their coverage of read bases, calculated from the seeds that make up the clusters. Sequential processing of these prioritized candidate regions halts when either a high number of feasible alignments has been found, a series of consecutive candidate regions failed to produce an acceptable alignment or read coverage drops below a certain threshold. An exception is made for regions containing seeds that are unique in the read. If no feasible alignments could be found, ALFALFA may invoke several rescue procedures that decrease the restrictions imposed on candidate regions and, if necessary, find a larger set of seeds.

### Chaining and alignment

Read mappers employ optimized dynamic programming algorithms to verify that candidate regions contain acceptable alignments. Such optimizations are needed since this is one of the most time-consuming steps in the read mapping process. The dimensions of a dynamic programming matrix correspond to the bounds of a candidate region, but computations are often restricted to a band around the main diagonal of the matrix. The width of this band depends on the minimal alignment score required.

**Figure 3:**
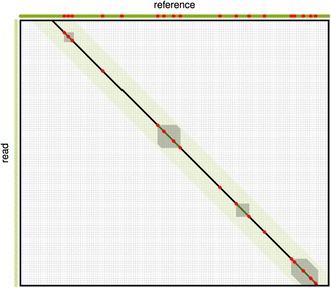
Example illustrating the cost of various dynamic programming algorithms. A typical alignment matrix to illustrating the magnitude of possible savings in computational cost by using a chain-guided alignment in comparison to standard banded dynamic programming. The figure shows the dynamic programming matrix for the semi-global alignment of a reference genome (rows) against a read (columns). The piecewise linear line represents the trace of an optimal alignment. Black parts of this line indicate locations of the seeds forming the chain. Red dots indicate mismatches in the alignment and green lines were not covered by seeds. The band size for banded dynamic programming is indicated in green and the areas in grey indicate the areas in which dynamic programming is performed for a chain-guided alignment. For this example, if banded alignment saves 86% of the matrix, chain-guided alignment saves 88% of the banded matrix.

ALFALFA further reduces the dimensions of the matrix by forming a collinear chain of a subset of the seeds that make up a candidate region. Dynamic programming can then be restricted to fill the gaps in between consecutive non-overlapping seeds. This technique has proven its value in whole genome alignment [16] and read mapping [14,17]. An example of the computational reduction achieved by chain-guided alignment can be seen in Figure 3.

The chaining algorithm starts from an anchor seed and greedily adds new seeds that do not exhibit a high skew to the chain. The skew is defined as the difference of the distances between two seeds on the read sequence and the reference genome. The amount of skew allowed is automatically decided based on the gap between the seeds and the parameters that influence the feasibility of an alignment. ALFALFA allows multiple chains per candidate region, based on the available anchor seeds. Anchor selection is based on seed length and seeds contained in chains can no longer be used as anchors in successive chain construction.

In evaluating candidate regions, ALFALFA supports both end-to-end and local alignment. Each of these alignment procedures starts with calculating a collinear chain of seeds and uses the same banded dynamic programming algorithm with a configurable scoring function that may take into account affine gap penalties. Insertions, deletions and single nucleotide polymorphisms in between consecutive non-overlapping seeds are handled without invoking the dynamic programming routine to avoid superfluous computations.

The final alignment and the mapping qualities are generated in a post-processing phase. By default, ALFALFA performs chain-guided alignment to obtain the CIGAR string, but an option can be enabled to use a banded dynamic programming routine over the full length of the read instead. Doing so increases the quality of the alignment, at the cost of a slightly reduced performance. We have found that the chain-guided alignment in this phase of the algorithm is on average 1.7 times faster than the banded dynamic programming approach.

### Paired-end reads

ALFALFA supports multiple strategies for mapping paired-end reads. The default strategy is commonly employed by read mappers: both ends are mapped independently of one another and alignments having corresponding orientations and locations with respect to certain insert size restrictions are subsequently paired. We have also implemented and tested several other strategies that concurrently map paired-end reads. One of these strategies has been used by Bowtie 2, among others, and maps one of the reads and then performs full dynamic programming to obtain the bridging alignment for the mate. The other strategies first identify candidate regions for both mates and then either prioritize extension based on the best regions of both mates or filter the list using the paired-end criteria. Similar to the single-end alignment algorithm, the paired-end algorithms can invoke rescue procedures if no concordant pair was found. In this case, the rescue procedures call upon one of the other strategies rather than decreasing the restrictiveness of some heuristics. Tests have shown that the best overall approach is the one that independently maps paired-end reads.

## Results and discussion

Execution speed, memory footprint and accuracy of ALFALFA have been scrutinized in a benchmark study that includes five other state-of-the-art long read mappers. All tests were run on a cluster with dual-socket quad-core Intel Xeon Nehalem (L5520) processors at clock speed 2.27 GHz and 12 GB RAM/node running Scientific Linux 6.5. Executables for ALFALFA v0.8, Bowtie v 2−2.2.3, BWA v 0.7.9*a* and CUSHAW v3.0.3 were built from source using gcc v4.4.7. Build 1.376 (beta) of GEM was obtained from its website, as source code was not available at the time of writing.

Two configurations of all read mappers were tested. First, read mappers were configured to produce a maximum of 4 alignments per read, if possible. Second, read mappers were configured to produce a single best alignment per read. Other parameters were kept to their default settings, unless the authors suggested specific settings for certain types of data (Supplementary Protocol in Additional file 1).

The human genome is used as reference genome to map a large array of moderately sized reads generated by current sequencing platforms and artificial reads generated by two simulators covering lengths expected to become commonplace in the near future. These simulated data sets are also crucial in evaluating mapping accuracy, which otherwise could not be evaluated objectively on true data. Care was taken to cover a broad range of error models observed in read sets generated by current sequencing technologies.

The wgsim simulator v0.3.1-r13 [18] — developed for SAMtools, but now a standalone project — was used to generate a series of single-end reads with lengths of one, five and ten thousand base pairs. Errors were introduced at rates between 2% and 10% of the total read length, with varying indel/mutation frequencies. Reads ranging from 100 bp to 10 kbp and abiding to specific error models induced by Illumina and 454 technologies were generated using the Mason simulator v0.1.1 [19]. Default parameter settings were used to generate reads of length 100 bp and 200 bp and parameter settings from the literature [5,12] were used for longer read data sets.

### Memory footprint

An index of the human genome assembly GRCh37 was constructed by all read mappers using their default parameter settings, except for GEM. A pre-built GEM index was downloaded from the GEM website as the indexer of this mapper ran into a fatal error on our test environment. Memory requirements of read mappers are mainly dependent on the memory footprint of the index structures they use. An overview of the index structure memory requirements can be found in Table 1. In this table, BWA-SW and BWA-MEM are reported as BWA, as they both use the same index structure. From the table, it can be seen that most tools require 3−5 GB of memory, both for storing the index on disk and for the peak memory during mapping a data set of 1kbp reads. Among the tested read mappers, CUSHAW3 seems to be the most memory efficient one. In contrast, ALFALFA requires twice as much memory as the other tools when configured with lower sparseness setting. The default setting (sparseness value 12) is competitive in terms of memory requirements with the other tools. The last column of Table 1 also shows index construction time, which is lowest for ALFALFA.

### Performance

**Figure 4:**
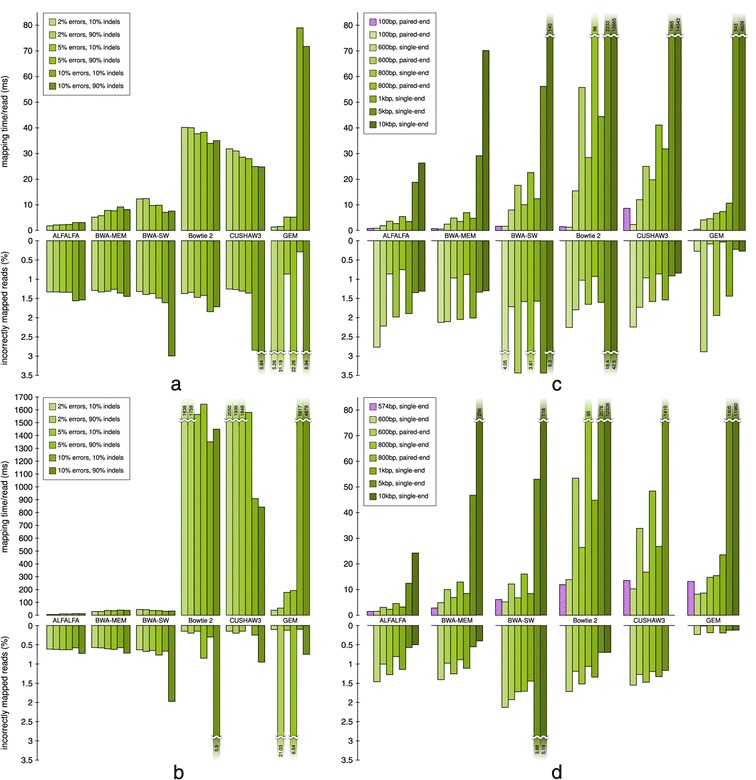
Accuracy and performance comparison of several long read mappers on real and simulated data. Test results on real data are indicated by purple bars and results on simulated data are represented by green bars. Data sets **(a)** and **(b)** are generated by the wgsim simulator and have a fixed length of respectively 1kbp and 5kbp, but vary in error rate and percentage of errors that are indels as specified in the legend. Data sets **(c)** and **(d)** are generated by the Mason simulator following Illumina **(c)** and 454 **(d)** error models and have varying read lengths as specified in the legend. Upper bars show the average mapping time per read (in milliseconds). Lower bars show the percentage of reads for which no alignment was found within 10bp of the simulated origin. Some results for mappers with a very low performance were gained from a data set that was ten or one hundred times smaller than the original data set (see Additional file 1).

Wall times of test runs were measured using the GNU/Linux time command. Performance results for most simulated and real data sets can be found in the upper barcharts in Figure 4, expressed in milliseconds per read. Runtime results for the complete benchmark can be found in Additional file 1.

ALFALFA is the fastest read mapper. It is only outperformed by GEM and BWA-MEM for the shortest reads and by GEM for a single data set of 1kbp reads. The lower performance of ALFALFA for shorter reads might be explained by an increased number of shorter MEM seeds and failure of one or more filtering heuristics when using the default parameters. The difference in runtime between ALFALFA and the other mappers increases with read length. For reads longer than 1kbp, ALFALFA, BWA-MEM and BWA-SW become orders of magnitude faster than the other mappers. Even compared to BWA-MEM, the second fastest mapper, ALFALFA is on average three times faster and up to five times faster for reads of at least 1kbp long. This can partially be explained by our automatic tuning of the minimum seed length, candidate region filtering heuristics and chain-guided alignment strategy. If memory is abundant, the runtime of ALFALFA can be further improved by lowering the sparseness of the index. ALFALFA is up to twice as fast when the sparseness is lowered from the default value of 12 to 4, the lowest setting tested.

The performance of GEM is mostly affected by the user-set error rate. For Illumina reads with low error rates and low error reads simulated with wgsim, GEM is among the fastest algorithms. For high error rate data sets, however, GEM becomes much slower. The performance of CUSHAW3 could be higher on hardware that supports SSE4 operations, which CUSHAW3 uses by default. However, CUSHAW3 is known to focus more on accuracy than speed [8]. For longer reads, the performance of Bowtie 2 is hampered by its dynamic programming alignment subroutine, whose runtime has a quadratic dependency on the read length. In addition, runtimes of CUSHAW3 dramatically increase when multiple alignments per read are requested (see Additional file 1). The performance of Bowtie 2 and BWA-MEM is also influenced by the number of alignments per read requested, but the increase in runtime is less significant.

For most mappers, there is no loss in performance when mapping single and paired-end reads. The exceptions are CUSHAW3 and Bowtie 2, whose performance is much lower when mapping paired-end reads due to the high read length and large insert size window and the fact that these mappers perform full dynamic programming to find an alignment for the mate of a mapped read.

Figure 4 also shows that the performance of ALFALFA, BWA-MEM and GEM decreases for reads containing more errors, whereas the performance of Bowtie 2, BWA-SW and CUSHAW3 increases. This could be explained by the fact that the latter mappers stop the alignment procedure more rapidly for reads that are more difficult to map, whereas the former increase the effort in finding an alignment for these reads. The type of errors, *i.e.* mutations versus indels, does not seem to have an effect on runtime.

We also assessed the scalability of ALFALFA with respect to the number of threads. To do so, we compared the speedup of ALFALFA to that of other read mappers for both single and paired-end read data sets. For 16 threads, the speedup of ALFALFA is on average 14.35, which is slightly lower than that of BWA-MEM (14.86) and CUSHAW3 (14.44), but higher than the speedup gained by BWA-SW (13.02), GEM (10.37) and Bowtie 2 (8.96). More details are shown in Additional file 1: Figure S8.

### Accuracy on simulated data

On simulated reads, accuracy was measured using the *recall rate* and our own definition of accuracy. Recall rate is defined as the number of reads for which an alignment is found within 10bp of the simulated origin. Our *accuracy* measure is less stringent and considers a read to be mapped correctly if an alignment either fulfills the recall rate requirements or has an edit distance that is not higher than the number of simulated differences to the reference genome. The lower bars in Figure 4 represent the recall rate in case each mapper reported a single alignment per read. When returning multiple alignments per read, a read is considered to be mapped correctly if at least one of the returned alignments fulfills the requirements imposed by our own accuracy measure. These additional results, together with the results using our own definition of accuracy, can be found in Additional file 1.

In contrast to the performance results, the difference in accuracy between the evaluated read mappers is small. All tested mappers exhibit both a high recall rate and accuracy when reporting either a single or up to four alignments per read. We will therefore refer to accuracy for both measures, unless we want to stress the difference between the two accuracy measures used. In general, CUSHAW3, BWA-MEM and ALFALFA are the most accurate mappers, with BWA-SW and Bowtie 2 having a somewhat lower accuracy. In most cases, either CUSHAW3, BWA-MEM or GEM is the most accurate mapper, by a small margin.

The accuracy of GEM is highly dependent on the command line parameter settings. We have tried several parameter settings to optimize the time-accuracy trade-off, but it is possible that GEM reaches a more optimal trade-off for untested parameter settings. As a result, the accuracy of GEM can vary greatly, being the highest for some data sets, but the lowest for other data sets. For example, on the 1kbp data sets with 2% errors, setting the parameters to this maximum error value results in a very low accuracy. In contrast, on 5% and 10% error rates, GEM has the highest recall rate for the data sets with low numbers of indel errors. The effect of the sparseness of the ESSA index on the accuracy of ALFALFA is depicted in Additional file 1: Figure S10, but is rather small in general.

From the results of the wgsim data sets in Figure 4, it can be seen that the accuracy of all mappers drops with increasing error rate. A noticable exception is GEM, whose accuracy depends on the chosen parameter settings. The effect of increasing error rate seems smallest for BWA-MEM, whereas CUSHAW3 does not perform well for reads with 10% errors. It is, however, possible to increase the accuracy of CUSHAW3 using command line parameters, as by default CUSHAW3 allows only 10% errors.

In addition to the raw error rate, an increase in the number of indel type errors has also a detrimental effect on accuracy. This effect seems smallest for Bowtie 2, whereas it has the highest effect on GEM.

In contrast to the above, an increase in read length has a predominantly positive effect on accuracy. For the longest reads, accuracy is almost 100% for most mappers. Note, however, that several of the results for Bowtie 2, CUSHAW3 and GEM were obtained on a smaller data set due to a forced timeout in our testing environment of 72 hours. Nonetheless, a few samples on a different machine indicated that these mappers indeed have a high accuracy at the cost of performance.

The type of errors also has an impact on accuracy. Wgsim simulated reads have a uniformly distributed error model, which differs from the Illumina and 454 error models. For equal read length, the accuracy on simulated reads with an Illumina error profile is lower than the accuracy on reads with a 454 error profile. For Illumina reads, CUSHAW3 is more accurate than BWA-MEM and ALFALFA, whereas the reverse in true on 454 reads.

The effect of paired-end read mapping on accuracy can be seen for the Mason simulated reads. As expected, paired-end read data sets exhibit a higher accuracy than single end read data sets. GEM hugely benefits from Illumina type paired-end reads. The only exception is BWA-SW, which performs worse for paired-end reads. This might be explained by the fact that BWA-SW automatically tries to estimate insert size, whereas other mappers trust on users to present insert size boundaries.

From the Additional tables in Additional file 1, we have found that the difference between the accuracy and recall rate measures is noticable for most mappers. The biggest effect was present in BWA-SW, Bowtie 2 and CUSHAW3, whereas the lowest effect was measured for GEM.

If multiple alignments per read are reported (see Additional file 1), the accuracy of several mappers increases significantly. The effect is the greatest for CUSHAW3 and ALFALFA, whereas it is lower for BWA-MEM and GEM. As a result, ALFALFA becomes the most accurate mapper for some data sets in this setting. In contrast, the accuracy of Bowtie 2 drops frequently, as the -k mode that is required to return multiple alignments works differently from the regular mode. Finally, BWA-SW does not offer an option to return multiple alignments per read.

### Mapping quality

In addition to accuracy and recall rate, we compared the sensitivity and specitivity of ALFALFA against that of other mappers. These evaluated measures are represented in receiver operating characteristic [ROC] curves in which the true positive rate is plotted against the false positive rate in terms of mapping quality values (MAPQ field in SAM files). For these plots, we limited ourselves to the wgsim simulated reads and used the evaluation script in the wgsim package to generate the data points. In addition to the wgsim simulated read data sets presented in Figure 4, we used a data set of single-end reads of length 600 bp with a small (1%) error rate.

**Figure 5:**
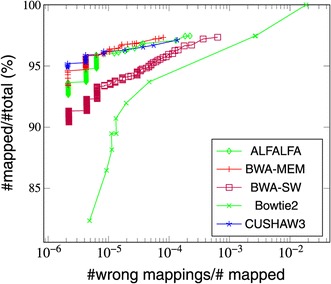
ROC curve for a data set of 600bp single-end reads with 1% error rate.

**Figure 6:**
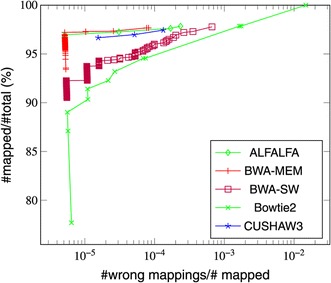
ROC curve for a data set of 1kbp single-end reads with 5% error rate.

Overall, Bowtie 2 has the highest sensitivity, which reaches 100%. However, Bowtie 2 is also less able to distinguish between good and bad alignments. CUSHAW3, BWA-MEM and ALFALFA exhibit the best trade-off between true positives and false positives. Figures 5 and 6 display the results for respectively the 600bp data set and a data set of 1 kbp reads with a 5% error rate.

For the 600 bp data set, CUSHAW3 is most sensitive for high mapping quality, whereas BWA-MEM becomes more sensitive for lower mapping quality values. ALFALFA obtains a trade-off that fluctuates between that of CUSHAW3 and BWA-MEM. For the 1 kbp data set with higher error rate, BWA-MEM is best able to distinguish between true and false positive hits, with ALFALFA a close second.

### Performance and accuracy on real data

To validate our findings on simulated data, we also compared the performance of ALFALFA on one real Illumina read data set and one real 454 data set. The results can be found in Table 2. Because the real origin of the reads cannot be indisputably determined, we use the sensitivity, *i.e.* the number of mapped reads as an accuracy measure.

**Table 2:** **Benchmark comparison of long read mappers on two real data sets**

	**Illumina reads**		**454 reads**	
	**Runtime**	**Sensitivity**	**Runtime**	**Sensitivity**
ALFALFA	5:48	99.09	0:33	99.75
BWA-MEM	5:19	99.71	1:04	99.60
BWA-SW	12:18	99.34	2:20	97.54
Bowtie 2	11:04	97.98	4:33	99.02
CUSHAW3	64:30	99.67	5:10	91.31
GEM	3:09	97.65	5:02	93.29

The Illumina paired-end read data set [SRA:ERR024139] consists of 2×100 bp reads and the reads of the 454 single-end data set [SRA:SRR003161] are on average 574 bp long. Performance measures are runtime in h:mm and percentage of mapped reads (sensitivity).

As our focus was on long reads, the Illumina read data set that consists of 2×100 bp paired-end reads falls out of the scope of ALFALFA (both in design choice and default parameter settings). As a result, it is outperformed by BWA-MEM and GEM in terms of mapping time and BWA-MEM, BWA-SW and CUSHAW3 in terms of sensitivity.

The single-end 454 reads have an average length of 574, which is well within the scope of our mapper. For this data set, ALFALFA is by far the fastest mapper. In addition, it also has the highest sensitivity. This is consistent with the good accuracy of ALFALFA for the simulated 454 reads.

## Conclusions

In this paper, we presented a novel long read mapper, called ALFALFA. The name is an acronym for “A Long Fragment Aligner/A Long Fragment Aligner". It is repeated twice as a pun on repetitive and overlapping fragments observed in genome sequences that heavily distort read mapping and genome assembly.

At a high level, ALFALFA is similar to other read mappers, as it implements the widely used seed-and-extend approach. However, ALFALFA featurs a novel index structure used for simultaneously finding MEMs and SMEMs, whose minimum length is automatically tuned to the read length and the number of errors. The seeds are used for identifying and selecting candidate alignment regions, taking into account the frequency of occurrence of the seeds in the reference sequence. In addition, the seeds are reused in a chain-guided alignment between the read and candidate alginment region. A more detailed algorithmic comparison between ALFALFA, BWA-MEM and CUSHAW3 is given in Additional file 1: Table S2.

Evaluation of read mapping algorithms requires a joint assessment of their accuracy, performance and memory footprint. Depending on specific properties of the test data, read mappers will often present different trade-offs between these evaluation criteria.

The benchmark results demonstrate that ALFALFA is extremely fast at mapping long reads, while still being competitive for moderately sized reads. Together with BWA-SW and BWA-MEM, it is one of a few mappers that scale well for read lengths up to several kilobases.

Measuring mapping accuracy can only be done objectively based on simulated reads whose original location on the reference genome is known. As mapping of long sequencing reads has not yet been benchmarked and sequencing platforms show various rates and types of errors, we examined a broad range of different read lengths and error models using two existing read simulators. These benchmark results show that in general all tested mappers have a high mapping accuracy for many of the tested data sets. In most of the test cases, ALFALFA is among the top most accurate mappers, with BWA-MEM, CUSHAW3 and GEM being slightly more accurate.

Memory requirements of ALFALFA are only slightly higher than most other long read mappers. In addition, ALFALFA features an interesting and easily tunable speed-memory trade-off by allowing users to specify the sparseness factor of the index.

Although the read lengths examined here are considered long at this moment, it would be interesting to evaluate the performance of ALFALFA and other read mappers for even longer reads, such as 100 kbp or 1 Mbp reads. As the alignment of such reads would approach the global alignment problem, other problems should be taken into account. For example, the presence of genomic rearrangements would require a more complex chaining algorithm.

In addition, a high number of errors, such as present in reads produced by Pacific Biosciences and Oxford Nanopore sequencers, remains a challenge to read mapping algorithms. Currently, ALFALFA relies on finding a few long exact seeds, and constructing chains of seeds with a small skew. To accomodate for these high error rates, we will investigate the use of inexact matches as seeds, and experiment with less restrictive chaining algorithms. Furthermore, additional information, such as base qualities and information on expected gap length could be incorporated to prioritize candidate region extension and to improve the dynamic programming subroutine and scoring system.

## Availability and requirements

- **Project name:** ALFALFA
- **Project home page:**http://alfalfa.ugent.be
- **Operating systems:** tested on Linux operating systems
- **Programming Language:** C++
- **Other requirements:** GCC v4.1.2 or higher
- **License:** New BSD License
- **Any restrictions to use by non-academics:** none

## Additional file

Additional file 1
**Document containing supplementary results, methods, protocol and program usage information.** This document contains several additional sections, tables, and figures. The tables contain full benchmark results. The Supplementary Methods section contains in-depth information on the ALFALFA mapping algorithm. The Supplementary Protocol section lists the details of the benchmark study, including a description of the read simulation process and parameters settings used for tuning the read mappers. The Supplementary Data section provides details on the command line options that can be used to tweak ALFALFA, their default settings and general usage tips.

## Abbreviations

ALFALFA: A Long Fragment Aligner/A Long Fragment Aligner
bp: base pairs
EBI: European Bioinformatics Institute
ESSA: Enhanced Sparse Suffix Array
GB: Gigabyte
GHz: Gigahertz
GNU: GNU’s Not Unix
indel: insertion or deletion
kbp: Kilo-base pair
Mbp: Mega-base pair
MEM: Maximal Exact Match
NCBI: National Center for Biotechnology Information
RAM: Random-access memory
SAM: Sequence Alignment Map
SMEM: Super-Maximal Exact Match

## References

[1] Li H, Homer N (2010). A survey of sequence alignment algorithms for next-generation sequencing. Brief Bioinform.

[2] Vyverman M, De Baets B, Fack V, Dawyndt P (2012). Prospects and limitations of full-text index structures in genome analysis. Nucleic Acids Res.

[3] Mason CE, Elemento O (2012). Faster sequencers, larger datasets, new challenges. Genome Biol.

[4] Marco-Sola S, Sammeth M, Ribeca P, Guigó R (2012). The GEM mapper: fast, accurate and versatile alignment by filtration. Nat Methods.

[5] Langmead B, Salzberg SL (2012). Fast gapped-read alignment with Bowtie 2. Nat Methods.

[6] Li H, Durbin R (2010). Fast and accurate long-read alignment with Burrows-Wheeler transform. Bioinformatics.

[7] Li H. Aligning sequence reads, clone sequences and assembly contigs with BWA-MEM. http://arxiv.org/abs/1303.3997.

[8] Liu Y, Popp B, Schmidt B (2014). CUSHAW3: sensitive and accurate base-space and color-space short-read alignment with hybrid seeding. PloS one.

[9] Vyverman M, De Baets B, Fack V, Dawyndt P (2013). essaMEM: finding maximal exact matches using enhanced sparse suffix arrays. Bioinformatics.

[10] Langmead B, Trapnell C, Pop M, Salzberg SL (2009). Ultrafast and memory-efficient alignment of short DNA sequences to the human genome. Genome Biol.

[11] Li H, Durbin R (2009). Fast and accurate short read alignment with Burrows-Wheeler transform. Bioinformatics.

[12] Weese D, Reinert K, Holtgrewe M (2012). RazerS 3: Faster, fully sensitive read mapping. Bioinformatics.

[13] Liu Y, Schmidt B (2012). Long read alignment based on maximal exact match seeds. Bioinformatics.

[14] Vyverman M, De Schrijver J, Van Criekinge W, Dawyndt P, Fack V, Pellegrini M, Fred A, Filipe J, Gamboa H (2011). Accurate long read mapping using enhanced suffix arrays. Proceedings of the International Conference on Bioinformatics Models, Methods and Algorithms (BIOINFORMATICS 2011).

[15] Li H (2012). Exploring single-sample SNP and INDEL calling with whole-genome de novo assembly. Bioinformatics.

[16] Kurtz S, Phillippy A, Delcher AL, Smoot M, Shumway M, Antonescu C, Salzberg SL (2004). Versatile and open software for comparing large genomes. Genome Biol.

[17] Hall IM, Faust G G (2012). YAHA: fast and flexible long-read alignment with optimal breakpoint detection. Bioinformatics.

[18] Li H. The wgsim read simulator. [https://github.com/lh3/wgsim]

[19] Holtgrewe M. Mason - a read simulator for second generation sequencing data. [http://www.seqan.de/projects/mason.html]

