# Supplementary material for: A Long Fragment Aligner called ALFALFA
Source: BMC Bioinformatics. 2015 May 15;16(1):159. doi: 10.1186/s12859-015-0533-0 (PMC4449525; doi:10.1186/s12859-015-0533-0)
Supplement: Additional file 1 — Document containing supplementary results, methods, protocol and program usage information. This document contains several additional sections, tables, and figures. The tables contain full benchmark results. The Supplementary Methods section contains in-depth information on the ALFALFA mapping algorithm. The Supplementary Protocol section lists the details of the benchmark study, including a description of the read simulation process and parameters settings used for tuning the read mappers. The Supplementary Data section provides details on the command line options that can be used to tweak ALFALFA, their default settings and general usage tips. [file 12859_2015_533_MOESM1_ESM.pdf]

A Long Fragment Aligner called ALFALFA

Michaël Vyverman[1], Bernard De Baets[2], Veerle Fack[1], and Peter Dawyndt*[1]

[1]Department of Applied Mathematics, Computer Science and Statistics, Ghent University, Krijgslaan 281 Building S9, B-9000 Ghent, Belgium
[2]Department of Mathematical Modelling, Statistics and Bioinformatics, Ghent University, Coupure links 653, B-9000 Ghent, Belgium

March 2015

This document contains several additional sections, tables, and figures.

- Table S1 contains detailed accuracy and performance results for several long read mappers on a large number of different long and moderately sized read sets.
- Figure S1 provides an accuracy and performance comparison of several long read mappers on simulated 10kbp single-end reads.
- Figures S2 to S7 contain ROC curves for several 1kbp data sets.
- Figure S8 provides an evaluation of the scalability of ALFALFA with respect to the number of threads.
- Figure S9 shows the effect of the sparseness of the ESSA index on the performance and accuracy of ALFALFA.
- Table S2 provides a comparison of the algorithmic design choices made in ALFALFA, BWA-MEM and CUSHAW3.
- Figure S10 shows the command line anatomy of ALFALFA.
- The Supplementary Methods section contains an in-depth description of the ALFALFA mapping and alignment algorithm. It also contains examples to illustrate and motivate specific design choices.
- The Supplementary Protocol section lists details of the benchmark study, including a description of the read simulation process and parameters settings used for tuning the read mappers.
- The Supplementary Data section provides details on the command line options that can to be used to tweak ALFALFA, their default settings and general usage tips.

*Corresponding author: Peter.Dawyndt@UGent.be

Table S1

Table S1 contains accuracy and performance results for several read mappers on an extensive benchmark of long and moderately sized read sets. In addition to the read mappers shown in Figures 4 and S1, results for ALFALFA with an index built using sparseness value 4 are also included. The difference in memory requirements between both indexes of ALFALFA can be found in Table 1.

For most mappers, results for two parameter settings are reported. The first setting triggers read mappers to return 4 alignments per read, or all for BWA-MEM. The second setting allows mappers to report only a single alignment per read. As an exception, BWA-SW does not provide the option to report multiple alignments.

Wall times for read mapping are reported excluding the time needed to build the index, but including time needed to generate the SAM output file. Some of the read mappers were run on a reduced set of reads due to the long runtimes for certain data sets. These cases are reported with the description of the individual data sets.

For all simulated data sets, the accuracy and recall rates are given. The recall rate is measured as the percentage of reads for which an alignment was found within 10bp of the simulated origin. For accuracy, a read is considered to be successfully mapped if an alignment was found within 10bp of the similated origin or an alignment was found containing fewer differences than the number of simulated errors. Most results are summarized in Figures 4 and S1, in which the percentage of incorrectly mapped reads (according to the recall measure) is plotted.

Table S1.1: Data set with 499998 simulated single-end reads, having a length of 600bp and containing 1% errors.

	multiple alignments			single alignment		
mapper	runtime	accuracy	recall	runtime	accuracy	recall
ALFALFA ($s = 4$)	0:06	99.91	99.76	0:06	99.27	98.36
ALFALFA ($s = 12$)	0:14	99.93	99.78	0:14	99.31	98.41
BWA-MEM	0:45	99.95	99.80	0:35	99.33	98.44
BWA-SW				0:50	99.19	98.31
Bowtie 2	2:09	98.20	97.71	1:58	99.07	98.16
CUSHAW3	4:07	99.95	99.79	1:33	99.36	98.44
GEM	1:08	99.99	99.98	1:09	99.90	99.89

Table S1.2: Data set with 199980 simulated single-end reads, having a length of 1kbp and containing 2% errors of which 10% are indels.

	multiple alignments			single alignment		
mapper	runtime	accuracy	recall	runtime	accuracy	recall
ALFALFA ($s = 4$)	0:03	99.98	99.88	0:03	99.67	98.70
ALFALFA ($s = 12$)	0:06	99.98	99.87	0:06	99.66	98.67
BWA-MEM	0:20	99.98	99.87	0:17	99.68	98.71
BWA-SW				0:41	99.56	98.68
Bowtie 2	2:30	99.16	98.59	2:13	99.60	98.62
CUSHAW3	5:21	99.98	99.87	1:46	99.69	98.74
GEM	0:04	94.79	94.78	0:04	94.77	94.74

Table S1.3: Data set with 199980 simulated single-end reads, having a length of 1kbp and containing 2% errors of which 90% are indels.

	multiple alignments			single alignment		
mapper	runtime	accuracy	recall	runtime	accuracy	recall
ALFALFA ($s = 4$)	0:03	99.88	99.86	0:03	98.99	98.65
ALFALFA ($s = 12$)	0:06	99.90	99.87	0:07	99.00	98.67
BWA-MEM	0:22	99.89	99.86	0:19	99.00	98.67
BWA-SW				0:41	98.95	98.60
Bowtie 2	2:35	99.13	98.72	2:13	99.02	98.66
CUSHAW3	5:23	99.90	99.87	1:43	99.10	98.73
GEM	0:05	68.95	68.94	0:05	68.92	68.81

Table S1.4: Data set with 199980 simulated single-end reads, having a length of 1kbp and containing 5% errors of which 10% are indels.

	multiple alignments			single alignment		
mapper	runtime	accuracy	recall	runtime	accuracy	recall
ALFALFA ($s = 4$)	0:05	99.93	99.85	0:04	99.42	98.61
ALFALFA ($s = 12$)	0:08	99.95	99.86	0:07	99.45	98.66
BWA-MEM	0:31	99.96	99.86	0:26	99.48	98.69
BWA-SW				0:32	99.40	98.63
Bowtie 2	2:18	99.13	98.07	2:05	99.38	98.53
CUSHAW3	4:46	99.95	99.86	1:35	99.54	98.69
GEM	0:17	99.27	99.26	0:17	99.21	99.13

Table S1.5: Data set with 199980 simulated single-end reads, having a length of 1kbp and containing 5% errors of which 90% are indels.

	multiple alignments			single alignment		
mapper	runtime	accuracy	recall	runtime	accuracy	recall
ALFALFA ($s = 4$)	0:05	99.85	99.85	0:05	98.82	98.67
ALFALFA ($s = 12$)	0:07	99.85	99.84	0:07	98.81	98.66
BWA-MEM	0:30	99.88	99.86	0:25	98.92	98.74
BWA-SW				0:32	98.80	98.51
Bowtie 2	2:21	99.18	98.28	2:07	98.80	98.58
CUSHAW3	4:47	99.85	99.83	1:33	98.88	98.64
GEM	0:17	78.01	77.98	0:17	77.97	77.74

Table S1.6: Data set with 199980 simulated single-end reads, having a length of 1kbp and containing 10% errors of which 10% are indels.

	multiple alignments			single alignment		
mapper	runtime	accuracy	recall	runtime	accuracy	recall
ALFALFA ($s = 4$)	0:07	99.47	98.95	0:06	98.76	97.75
ALFALFA ($s = 12$)	0:10	99.81	99.67	0:10	99.11	98.44
BWA-MEM	0:36	99.92	99.84	0:30	99.31	98.64
BWA-SW				0:23	99.16	98.39
Bowtie 2	1:59	99.09	96.99	1:53	98.98	98.16
CUSHAW3	2:43	98.42	98.32	1:23	97.88	97.15
GEM	4:24	99.97	99.94	4:23	99.88	99.71

Table S1.7: Data set with 199980 simulated single-end reads, having a length of 1kbp and containing 10% errors of which 90% are indels.

	multiple alignments			single alignment		
mapper	runtime	accuracy	recall	runtime	accuracy	recall
ALFALFA ($s = 4$)	0:07	99.53	99.28	0:07	98.34	98.03
ALFALFA ($s = 12$)	0:10	99.72	99.67	0:10	98.59	98.46
BWA-MEM	0:32	99.83	99.75	0:27	98.81	98.55
BWA-SW				0:25	97.83	97.01
Bowtie 2	2:06	99.14	97.56	1:56	98.59	98.28
CUSHAW3	2:51	95.52	95.37	1:22	94.51	94.16
GEM	4:01	91.60	91.50	3:59	91.53	91.06

Table S1.8: Data set with 199976 simulated single-end reads, having a length of 5kbp and containing 2% errors of which 10% are indels. [§]Measurements restricted to the first 2000 reads.

	multiple alignments			single alignment		
mapper	runtime	accuracy	recall	runtime	accuracy	recall
ALFALFA ($s = 4$)	0:15	99.99	99.98	0:15	99.61	99.36
ALFALFA ($s = 12$)	0:20	99.99	99.98	0:20	99.63	99.39
BWA-MEM	1:40	99.99	99.98	1:36	99.66	99.43
BWA-SW				2:30	99.61	99.37
Bowtie 2[§]	2:28	99.90	99.90	1:01	99.90	99.85
CUSHAW3[§]	5:44	100.00	100.00	1:08	99.90	99.85
GEM	2:06	99.95	99.95	2:06	99.93	99.89

Table S1.9: Data set with 199976 simulated single-end reads, having a length of 5kbp and containing 2% errors of which 90% are indels. [§]Measurements restricted to the first 2000 reads.

	multiple alignments			single alignment		
mapper	runtime	accuracy	recall	runtime	accuracy	recall
ALFALFA ($s = 4$)	0:15	99.98	99.98	0:15	99.39	99.37
ALFALFA ($s = 12$)	0:20	99.98	99.98	0:19	99.40	99.38
BWA-MEM	1:40	99.98	99.98	1:36	99.44	99.42
BWA-SW				2:25	99.36	99.33
Bowtie 2[§]	2:43	99.90	99.85	0:58	99.80	99.80
CUSHAW3[§]	5:40	100.00	100.00	1:04	99.80	99.80
GEM	3:04	79.10	79.10	3:05	79.08	78.97

Table S1.10: Data set with 199976 simulated single-end reads, having a length of 5kbp and containing 5% errors of which 10% are indels. [§]Measurements restricted to the first 2000 reads.

	multiple alignments			single alignment		
mapper	runtime	accuracy	recall	runtime	accuracy	recall
ALFALFA ($s = 4$)	0:25	99.98	99.98	0:24	99.48	99.35
ALFALFA ($s = 12$)	0:36	99.98	99.98	0:35	99.48	99.37
BWA-MEM	2:08	99.99	99.98	2:03	99.52	99.39
BWA-SW				2:03	99.48	99.35
Bowtie 2[§]	2:19	99.90	99.90	0:52	99.90	99.85
CUSHAW3[§]	5:11	100.00	100.00	1:01	99.90	99.85
GEM	9:57	99.99	99.99	9:52	99.95	99.88

Table S1.11: Data set with 199976 simulated single-end reads, having a length of 5kbp and containing 5% errors of which 90% are indels. [§]Measurements restricted to the first 2000 reads.

	multiple alignments			single alignment		
mapper	runtime	accuracy	recall	runtime	accuracy	recall
ALFALFA ($s = 4$)	0:24	99.98	99.98	0:24	99.38	99.38
ALFALFA ($s = 12$)	0:34	99.98	99.98	0:33	99.38	99.37
BWA-MEM	2:05	99.98	99.98	1:57	99.39	99.38
BWA-SW				1:58	99.27	99.23
Bowtie 2[§]	2:18	99.20	99.20	0:54	99.20	99.15
CUSHAW3[§]	5:10	100.00	100.00	0:52	100.00	100.00
GEM	10:37	93.72	93.70	10:42	93.69	93.46

Table S1.12: Data set with 199976 simulated single-end reads, having a length of 5kbp and containing 10% errors of which 10% are indels. [§]Measurements restricted to the first 2000 reads.

	multiple alignments			single alignment		
mapper	runtime	accuracy	recall	runtime	accuracy	recall
ALFALFA ($s = 4$)	0:29	99.95	99.92	0:29	99.43	99.35
ALFALFA ($s = 12$)	0:42	99.97	99.97	0:42	99.47	99.42
BWA-MEM	2:20	99.98	99.98	2:14	99.51	99.42
BWA-SW				1:42	99.43	99.33
Bowtie 2[§]	2:10	99.95	99.95	0:45	99.70	99.70
CUSHAW3[§]	2:00	100.00	100.00	0:30	99.75	99.75
GEM[§]	3:07	100.00	100.00	3:07	99.95	99.90

Table S1.13: Data set with 199976 simulated single-end reads, having a length of 5kbp and containing 10% errors of which 90% are indels. [§]Measurements restricted to the first 2000 reads.

	multiple alignments			single alignment		
mapper	runtime	accuracy	recall	runtime	accuracy	recall
ALFALFA ($s = 4$)	0:30	99.90	99.89	0:31	99.23	99.22
ALFALFA ($s = 12$)	0:42	99.91	99.91	0:41	99.28	99.27
BWA-MEM	2:15	99.91	99.91	2:07	99.30	99.28
BWA-SW				1:47	98.53	98.03
Bowtie 2[§]	2:00	94.25	94.25	0:48	94.10	94.10
CUSHAW3[§]	2:02	99.20	99.20	0:28	99.05	99.05
GEM[§]	2:43	99.60	99.45	2:42	99.55	99.25

Table S1.14: Data set with 199922 simulated single-end reads, having a length of 10kbp and containing 2% errors of which 10% are indels. §Measurements restricted to the first 2000 reads.

	multiple alignments			single alignment		
mapper	runtime	accuracy	recall	runtime	accuracy	recall
ALFALFA ($s = 4$)	0:32	99.99	99.99	0:32	99.64	99.57
ALFALFA ($s = 12$)	0:36	100.00	99.99	0:35	99.64	99.57
BWA-MEM	3:19	99.99	99.99	3:11	99.71	99.65
BWA-SW				5:34	99.69	99.61
Bowtie 2§	12:17	99.90	99.90	5:00	99.95	99.95
CUSHAW3§	43:51	100.00	100.00	7:55	99.95	99.95
GEM	12:19	99.99	99.99	12:27	99.97	99.93

Table S1.15: Data set with 199922 simulated single-end reads, having a length of 10kbp and containing 2% errors of which 90% are indels. §Measurements restricted to the first 2000 reads.

	multiple alignments			single alignment		
mapper	runtime	accuracy	recall	runtime	accuracy	recall
ALFALFA ($s = 4$)	0:33	99.99	99.99	0:33	99.59	99.59
ALFALFA ($s = 12$)	0:36	99.99	99.99	0:36	99.60	99.59
BWA-MEM	3:13	99.99	99.99	3:15	99.63	99.63
BWA-SW				5:04	99.56	99.54
Bowtie 2§	12:15	99.70	99.70	5:18	99.65	99.65
CUSHAW3§	43:33	100.00	100.00	7:37	99.85	99.85
GEM	18:23	86.24	86.24	18:36	86.23	86.12

Table S1.16: Data set with 199922 simulated single-end reads, having a length of 10kbp and containing 5% errors of which 10% are indels. §Measurements restricted to the first 2000 reads.

	multiple alignments			single alignment		
mapper	runtime	accuracy	recall	runtime	accuracy	recall
ALFALFA ($s = 4$)	0:45	99.99	99.99	0:45	99.62	99.59
ALFALFA ($s = 12$)	0:54	99.99	99.99	0:54	99.63	99.60
BWA-MEM	4:15	99.99	99.99	4:01	99.66	99.62
BWA-SW				4:21	99.63	99.59
Bowtie 2§	10:57	99.95	99.95	4:12	100.00	100.00
CUSHAW3§	39:43	100.00	100.00	6:57	99.95	99.95
GEM	64:15	99.99	99.98	62:43	99.95	99.87

Table S1.17: Data set with 199922 simulated single-end reads, having a length of 10kbp and containing 5% errors of which 90% are indels. §Measurements restricted to the first 2000 reads.

	multiple alignments			single alignment		
mapper	runtime	accuracy	recall	runtime	accuracy	recall
ALFALFA ($s = 4$)	0:49	99.98	99.98	0:48	99.54	99.54
ALFALFA ($s = 12$)	0:56	99.98	99.98	0:56	99.54	99.54
BWA-MEM	4:05	99.99	99.99	4:02	99.60	99.60
BWA-SW				3:55	99.24	99.09
Bowtie 2§	12:00	96.60	96.60	5:08	96.55	96.55
CUSHAW3§	40:04	100.00	100.00	6:45	99.95	99.95
GEM§	0:39	98.10	98.10	0:39	98.10	98.10

Table S1.18: Data set with 199922 simulated single-end reads, having a length of 10kbp and containing 10% errors of which 10% are indels. §Measurements restricted to the first 2000 reads.

	multiple alignments			single alignment		
mapper	runtime	accuracy	recall	runtime	accuracy	recall
ALFALFA ($s = 4$)	1:03	99.96	99.87	1:01	99.55	99.46
ALFALFA ($s = 12$)	1:09	99.98	99.97	1:07	99.59	99.57
BWA-MEM	4:29	99.99	99.99	4:27	99.64	99.61
BWA-SW				3:30	99.60	99.56
Bowtie 2§	10:26	99.85	99.85	3:43	99.85	99.85
CUSHAW3§	15:11	100.00	100.00	3:08	99.95	99.90
GEM§	25:22	100.00	100.00	23:56	100.00	99.95

Table S1.19: Data set with 199922 simulated single-end reads, having a length of 10kbp and containing 10% errors of which 90% are indels. §Measurements restricted to the first 2000 reads.

	multiple alignments			single alignment		
mapper	runtime	accuracy	recall	runtime	accuracy	recall
ALFALFA ($s = 4$)	1:10	99.91	99.88	1:08	99.44	99.41
ALFALFA ($s = 12$)	1:14	99.93	99.92	1:10	99.48	99.47
BWA-MEM	4:30	99.92	99.92	4:22	99.50	99.50
BWA-SW				3:43	97.43	96.02
Bowtie 2§	9:50	90.60	90.60	3:59	90.60	90.60
CUSHAW3§	15:49	99.80	99.80	3:02	99.75	99.75
GEM§	21:48	99.95	99.85	21:39	99.95	99.85

Table S1.20: Data set with 2 × 1 million simulated paired-end reads, having a length of 100bp and generated with Illumina error model. Insert size is 300bp with a standard deviation of 10%.

	multiple alignments			single alignment		
mapper	runtime	accuracy	recall	runtime	accuracy	recall
ALFALFA ($s = 4$)	0:23	99.53	98.30	0:21	99.48	97.02
ALFALFA ($s = 12$)	0:14	99.56	98.51	0:14	99.51	97.23
BWA-MEM	0:10	99.95	97.88	0:09	99.95	97.88
BWA-SW				0:27	99.91	95.95
Bowtie 2	0:27	99.85	98.90	0:21	99.89	97.75
CUSHAW3	0:41	99.95	99.40	0:38	99.92	97.76
GEM	0:08	99.92	99.73	0:08	99.92	99.73

Table S1.21: Data set with 2 × 1 million simulated paired-end reads, having a length of 200bp and generated with Illumina error model. Insert size is 3kbp with a standard deviation of 10%.

	multiple alignments			single alignment		
mapper	runtime	accuracy	recall	runtime	accuracy	recall
ALFALFA ($s = 4$)	0:11	99.89	99.52	0:10	99.80	98.51
ALFALFA ($s = 12$)	0:21	99.89	99.56	0:20	99.80	98.57
BWA-MEM	0:18	99.88	98.57	0:19	99.88	98.57
BWA-SW				1:17	99.83	97.58
Bowtie 2	3:10	99.80	99.33	2:06	99.85	98.49
CUSHAW3	1:35	99.98	99.76	1:05	99.89	98.54
GEM	0:15	99.99	99.97	0:15	99.99	99.97

Table S1.22: Data set with 1 million simulated single-end reads, having a length of 600bp and generated with Illumina error model.

	multiple alignments			single alignment		
mapper	runtime	accuracy	recall	runtime	accuracy	recall
ALFALFA ($s = 4$)	0:14	98.97	98.96	0:13	97.82	97.77
ALFALFA ($s = 12$)	0:30	98.99	98.97	0:31	97.83	97.78
BWA-MEM	0:51	99.05	99.04	0:41	97.94	97.90
BWA-SW				2:12	98.47	98.28
Bowtie 2	4:47	98.46	98.28	4:17	98.26	98.20
CUSHAW3	7:26	99.46	99.42	3:20	98.35	98.27
GEM	1:09	97.21	97.21	1:08	97.11	97.11

Table S1.23: Data set with 2 × 1 million simulated paired-end reads, having a length of 600bp and generated with Illumina error model. Insert size is 3kbp with a standard deviation of 10%.

	multiple alignments			single alignment		
mapper	runtime	accuracy	recall	runtime	accuracy	recall
ALFALFA ($s = 4$)	0:26	99.84	99.83	0:25	99.17	99.13
ALFALFA ($s = 12$)	0:57	99.85	99.83	0:59	99.18	99.14
BWA-MEM	1:21	99.08	99.03	1:21	99.08	99.03
BWA-SW				4:53	98.20	96.56
Bowtie 2	23:01	99.07	99.00	15:29	99.03	98.97
CUSHAW3	37:33	99.91	99.90	6:56	99.10	99.04
GEM	1:16	99.92	99.92	1:16	99.92	99.92

Table S1.24: Data set with 1 million simulated single-end reads, having a length of 800bp and generated with Illumina error model.

	multiple alignments			single alignment		
mapper	runtime	accuracy	recall	runtime	accuracy	recall
ALFALFA ($s = 4$)	0:28	99.06	99.06	0:28	98.02	98.00
ALFALFA ($s = 12$)	0:43	99.08	99.08	0:45	98.03	98.02
BWA-MEM	1:14	99.01	99.00	0:58	97.97	97.95
BWA-SW				2:48	98.53	98.42
Bowtie 2	8:47	98.57	98.40	7:54	98.36	98.35
CUSHAW3	13:31	99.48	99.47	5:30	98.45	98.42
GEM	1:53	98.17	98.17	1:51	98.06	98.06

Table S1.25: Data set with 2 × 1 million simulated paired-end reads, having a length of 800bp and generated with Illumina error model. Insert size is 3kbp with a standard deviation of 10%.

	multiple alignments			single alignment		
mapper	runtime	accuracy	recall	runtime	accuracy	recall
ALFALFA ($s = 4$)	0:55	99.89	99.89	0:57	99.25	99.24
ALFALFA ($s = 12$)	1:30	99.90	99.90	1:30	99.26	99.25
BWA-MEM	1:55	99.14	99.12	1:55	99.14	99.12
BWA-SW				6:16	97.83	96.39
Bowtie 2	40:10	99.01	98.94	26:42	99.09	99.07
CUSHAW3	71:50	99.93	99.93	11:25	99.16	99.14
GEM	2:02	99.97	99.97	2:03	99.97	99.97

Table S1.26: Data set with 100000 simulated single-end reads, having a length of 1kbp and generated with Illumina error model.

	multiple alignments			single alignment		
mapper	runtime	accuracy	recall	runtime	accuracy	recall
ALFALFA ($s = 4$)	0:03	99.09	99.09	0:03	98.13	98.13
ALFALFA ($s = 12$)	0:06	99.11	99.11	0:05	98.11	98.11
BWA-MEM	0:09	99.01	99.01	0:07	97.99	97.99
BWA-SW				0:20	98.53	98.43
Bowtie 2	1:28	98.61	98.44	1:14	98.41	98.40
CUSHAW3	2:28	99.44	99.43	0:53	98.47	98.46
GEM	0:18	98.71	98.71	0:17	98.56	98.56

Table S1.27: Data set with 50000 simulated single-end reads, having a length of 5kbp and generated with Illumina error model. [§]Measurements for *single alignment* restricted to the first 5000 reads. [†]Measurements for *multiple alignments* restricted to the first 5000 reads.

	multiple alignments			single alignment		
mapper	runtime	accuracy	recall	runtime	accuracy	recall
ALFALFA ($s = 4$)	0:09	99.10	99.10	0:09	98.63	98.63
ALFALFA ($s = 12$)	0:15	99.12	99.12	0:15	98.65	98.65
BWA-MEM	0:25	99.12	99.12	0:24	98.66	98.66
BWA-SW				0:46	96.85	96.56
Bowtie 2[§]	20:05	98.93	98.47	3:06	81.60	81.60
CUSHAW3[†]	13:03	99.80	99.80	27:18	99.09	99.09
GEM	7:32	99.88	99.88	7:32	99.78	99.78

Table S1.28: Data set with 10000 simulated single-end reads, having a length of 10kbp and generated with Illumina error model. [§]Measurements for *single alignment* restricted to the first 1000 reads. [†]Measurements for *multiple alignments* restricted to the first 1000 reads.

	multiple alignments			single alignment		
mapper	runtime	accuracy	recall	runtime	accuracy	recall
ALFALFA ($s = 4$)	0:03	98.97	98.97	0:03	98.62	98.62
ALFALFA ($s = 12$)	0:04	98.98	98.98	0:04	98.69	98.69
BWA-MEM	0:12	98.98	98.98	0:11	98.70	98.70
BWA-SW				0:23	94.63	93.70
Bowtie 2[§]	21:21	99.23	98.47	3:31	57.5	57.5
CUSHAW3[†]	19:43	99.95	99.95	40:23	99.16	99.16
GEM	14:56	99.86	99.86	12:51	99.73	99.73

Table S1.29: Data set with 1 million simulated single-end reads, having an average length of 600bp and generated with the 454 error model.

	multiple alignments			single alignment		
mapper	runtime	accuracy	recall	runtime	accuracy	recall
ALFALFA ($s = 4$)	0:15	99.64	99.54	0:15	98.73	98.36
ALFALFA ($s = 12$)	0:26	99.75	99.69	0:25	98.86	98.54
BWA-MEM	1:44	99.84	99.77	1:21	99.02	98.59
BWA-SW				1:26	98.63	97.87
Bowtie 2	4:08	98.39	97.43	3:51	98.71	98.28
CUSHAW3	6:46	99.71	99.63	2:51	99.00	98.45
GEM	2:19	99.93	99.91	2:16	99.88	99.76

Table S1.30: Data set with 2×1 million simulated paired-end reads, having an average length of 600bp and generated with the 454 error model. Insert size is 3kbp with a standard deviation of 10%.

	multiple alignments			single alignment		
mapper	runtime	accuracy	recall	runtime	accuracy	recall
ALFALFA ($s = 4$)	0:30	99.64	99.39	0:28	99.18	98.65
ALFALFA ($s = 12$)	0:51	99.81	99.70	0:51	99.38	99.00
BWA-MEM	2:44	99.53	99.02	2:46	99.53	99.02
BWA-SW				3:22	99.32	98.07
Bowtie 2	21:35	99.22	98.55	14:50	99.36	98.81
CUSHAW3	19:29	99.53	98.94	9:24	99.48	98.73
GEM	2:05	99.99	99.99	2:23	99.99	99.99

Table S1.31: Data set with 1 million simulated single-end reads, having an average length of 800bp and generated with the 454 error model.

	multiple alignments			single alignment		
mapper	runtime	accuracy	recall	runtime	accuracy	recall
ALFALFA ($s = 4$)	0:21	99.77	99.74	0:21	98.86	98.65
ALFALFA ($s = 12$)	0:37	99.83	99.80	0:38	98.93	98.72
BWA-MEM	2:13	99.87	99.83	1:54	99.03	98.74
BWA-SW				1:53	98.83	98.28
Bowtie 2	7:50	98.64	97.68	7:20	98.78	98.48
CUSHAW3	10:27	99.69	99.64	4:40	98.91	98.52
GEM	4:08	99.97	99.96	4:06	99.92	99.81

Table S1.32: Data set with 2×1 million simulated paired-end reads, having an average length of 800bp and generated with the 454 error model. Insert size is 3kbp with a standard deviation of 10%.

	multiple alignments			single alignment		
mapper	runtime	accuracy	recall	runtime	accuracy	recall
ALFALFA ($s = 4$)	0:41	99.79	99.70	0:40	99.32	99.05
ALFALFA ($s = 12$)	1:16	99.88	99.83	1:15	99.43	99.19
BWA-MEM	3:37	99.48	99.11	3:35	99.48	99.11
BWA-SW				4:27	99.20	98.29
Bowtie 2	36:40	99.24	98.50	26:27	99.32	98.94
CUSHAW3	26:05	99.41	98.96	13:26	99.35	98.80
GEM	4:16	100.00	99.99	4:17	100.00	99.99

Table S1.33: Data set with 100000 simulated single-end reads, having an average length of 1kbp and generated with the 454 error model.

	multiple alignments			single alignment		
mapper	runtime	accuracy	recall	runtime	accuracy	recall
ALFALFA ($s = 4$)	0:03	99.81	99.78	0:03	98.96	98.81
ALFALFA ($s = 12$)	0:05	99.86	99.84	0:05	99.03	98.86
BWA-MEM	0:16	99.89	99.87	0:14	99.10	98.89
BWA-SW				0:14	98.94	98.56
Bowtie 2	1:20	98.86	97.88	1:14	98.89	98.66
CUSHAW3	1:52	99.68	99.64	0:44	98.96	98.67
GEM	0:39	99.98	99.96	0:39	99.92	99.81

Table S1.34: Data set with 50000 simulated single-end reads, having an average length of 5kbp and generated with the 454 error model. §Measurements for *single alignment* restricted to the first 5000 reads. †Measurements for *multiple alignments* restricted to the first 5000 reads.

	multiple alignments			single alignment		
mapper	runtime	accuracy	recall	runtime	accuracy	recall
ALFALFA ($s = 4$)	0:08	99.97	99.97	0:08	99.46	99.46
ALFALFA ($s = 12$)	0:10	99.97	99.97	0:10	99.43	99.43
BWA-MEM	0:42	99.98	99.98	0:38	99.45	99.45
BWA-SW				0:44	99.24	96.12
Bowtie 2§	28:08	99.72	98.00	2:53	99.30	99.30
CUSHAW3†	8:20	99.5	99.5	19:35	98.97	98.83
GEM	19:35	100.00	100.00	19:31	99.93	99.88

Table S1.35: Data set with 10000 simulated single-end reads, having an average length of 10kbp and generated with the 454 error model. [§]Measurements for *single alignment* restricted to the first 1000 reads. [†]Fatal error encountered during mapping process.

	multiple alignments			single alignment		
mapper	runtime	accuracy	recall	runtime	accuracy	recall
ALFALFA ($s = 4$)	0:04	99.46	98.45	0:04	99.23	98.22
ALFALFA ($s = 12$)	0:03	99.89	99.79	0:04	99.60	99.50
BWA-MEM	0:44	99.96	99.96	0:42	99.60	99.60
BWA-SW				0:19	99.98	94.81
Bowtie 2[§]	35:23	99.95	97.92	3:25	99.30	99.30
CUSHAW3[†]	*N.A.*	*N.A.*	*N.A.*	*N.A.*	*N.A.*	*N.A.*
GEM	33:27	100.00	100.00	33:13	99.94	99.88

Accuracy-time trade-offs for 10kbp reads

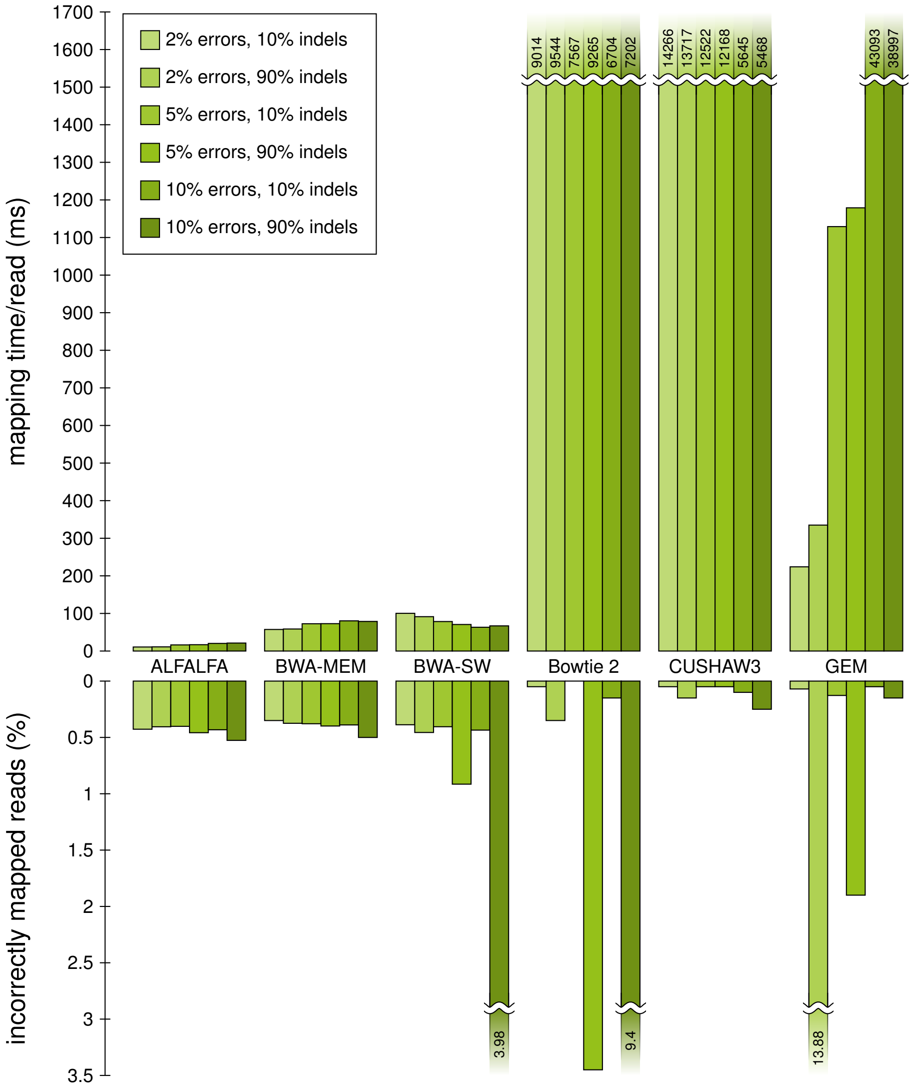

Figure S1: Accuracy and performance comparison of several long read mappers on 200000 simulated 10kbp single-end reads. The six different data sets vary in error rate and percentage of errors that are indels as specified in the legend. For ALFALFA, BWA-MEM and BWA-SW the entire data set was used to obtain the results, but a data set that was one hundred times smaller was used for Bowtie 2 and CUSHAW3 due to the low performance. For GEM, the first three results were obtained using the full data sets and the last three using the reduced data set. Upper bars show the average mapping time per read (in milliseconds), whereas lower bars show the percentage of reads for which no alignment was found within 10bp of the simulated origin. All tools were configured to produce a maximum of one alignment per read.

Sensitivity-specificity trade-offs for 1kbp reads

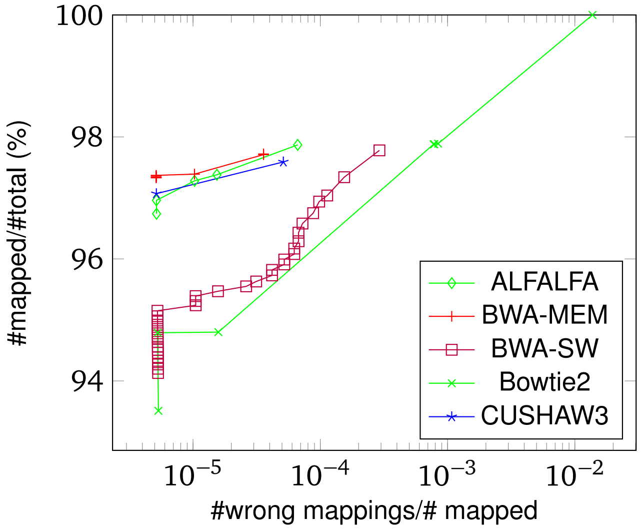

Figure S2: ROC curve plotting the sensitivity (vertical axis) against the false positive rate (horizontal axis) using different mapping quality cut-offs (MAPQ> 0), for a data set with 199980 simulated single-end reads, having a length of 1kbp and containing 2% errors of which 10% are indels.

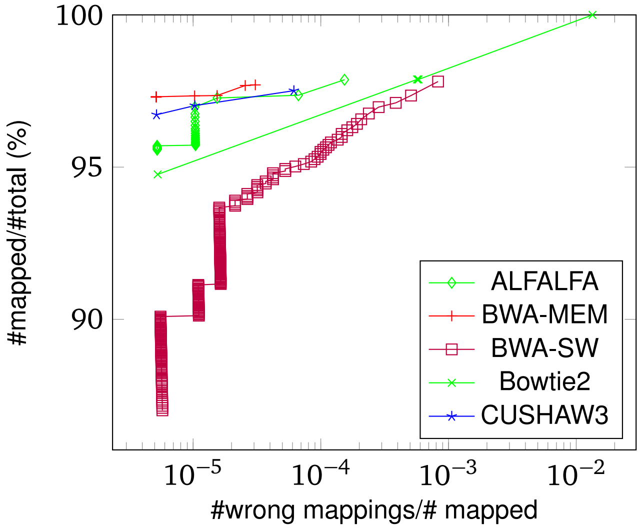

Figure S3: ROC curve plotting the sensitivity (vertical axis) against the false positive rate (horizontal axis) using different mapping quality cut-offs (MAPQ> 0), for a data set with 199980 simulated single-end reads, having a length of 1kbp and containing 2% errors of which 90% are indels.

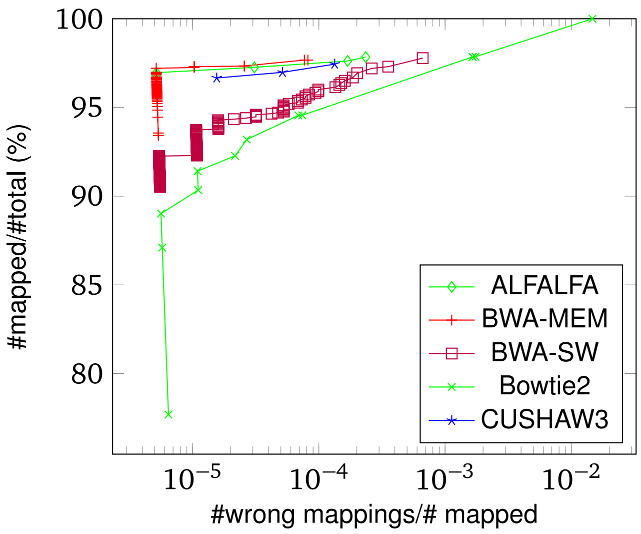

Figure S4: ROC curve plotting the sensitivity (vertical axis) against the false positive rate (horizontal axis) using different mapping quality cut-offs (MAPQ> 0), for a data set with 199980 simulated single-end reads, having a length of 1kbp and containing 5% errors of which 10% are indels.

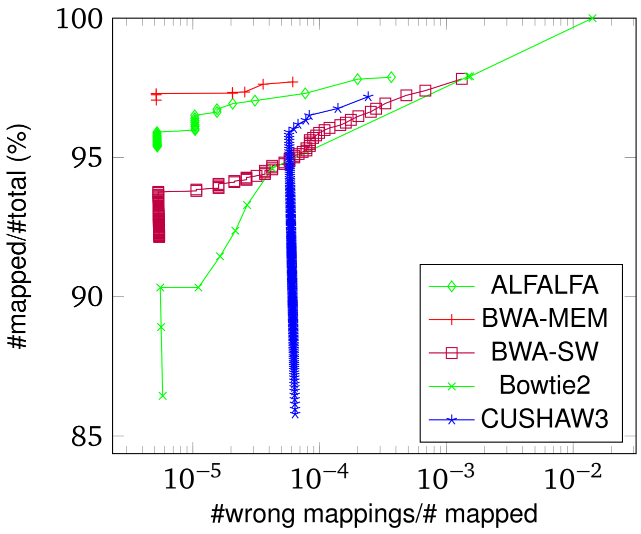

Figure S5: ROC curve plotting the sensitivity (vertical axis) against the false positive rate (horizontal axis) using different mapping quality cut-offs (MAPQ> 0), for a data set with 199980 simulated single-end reads, having a length of 1kbp and containing 5% errors of which 90% are indels.

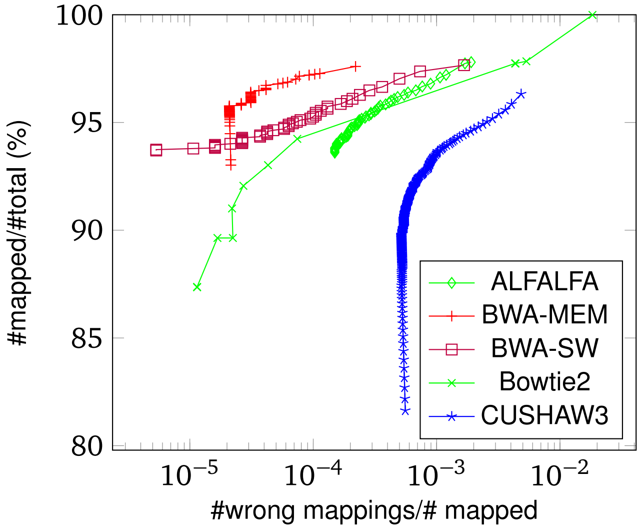

Figure S6: ROC curve plotting the sensitivity (vertical axis) against the false positive rate (horizontal axis) using different mapping quality cut-offs (MAPQ> 0), for a data set with 199980 simulated single-end reads, having a length of 1kbp and containing 10% errors of which 10% are indels.

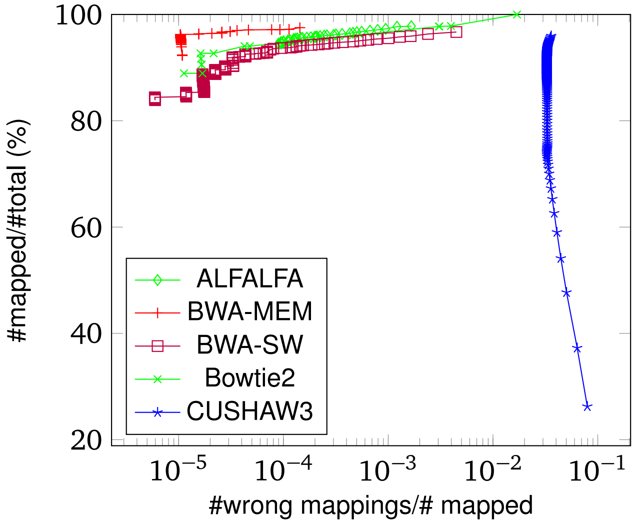

Figure S7: ROC curve plotting the sensitivity (vertical axis) against the false positive rate (horizontal axis) using different mapping quality cut-offs (MAPQ> 0), for a data set with 199980 simulated single-end reads, having a length of 1kbp and containing 10% errors of which 90% are indels.

Evaluation of the scalability

In addition to the single-threaded performance of ALFALFA, we assessed its scalability with respect to the number of threads. The multithreading support of ALFALFA is based on the design used in BWA-MEM. Batches of reads are first read by a single thread and then processed in parallel using the `kthread` class from the *klib* library [1]. We compared the speedup of ALFALFA to that of the other tools for both single and paired-end read data sets. For 16 threads, the speedup of ALFALFA is on average 14.35, which is slightly lower than that of BWA-MEM (14.86) and CUSHAW3 (14.44), but higher than the speedup gained by BWA-SW (13.02), GEM (10.37) and Bowtie 2 (8.96).

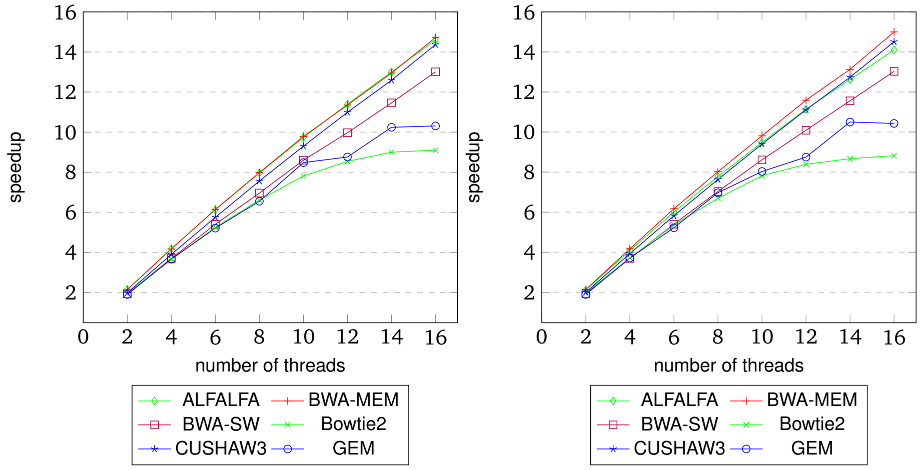

(a) single-end reads (b) paired-end reads

Figure S8: Comparison of the scalability of read mappers with respect to the number of threads. The results show the speedup gained in function of the number of threads (all even values, up to and including 16 threads). Single-threaded runtimes can be found in Tables S1.24 and S1.25 for respectively Figure S8a and Figure S8b. All tests were run on a cluster with dual-socket octa-core Intel Xeon Sandy Bridge ($E5 - 2670$) processors at clock speed 2.6GHz and 32GB RAM/node running Scientific Linux 6.1 and the gcc v4.4.7 compiler was used to build all read mappers from source code, except for GEM which is not open source.

[1] https://github.com/attractivechaos/klib

Effect of sparseness on performance of ALFALFA

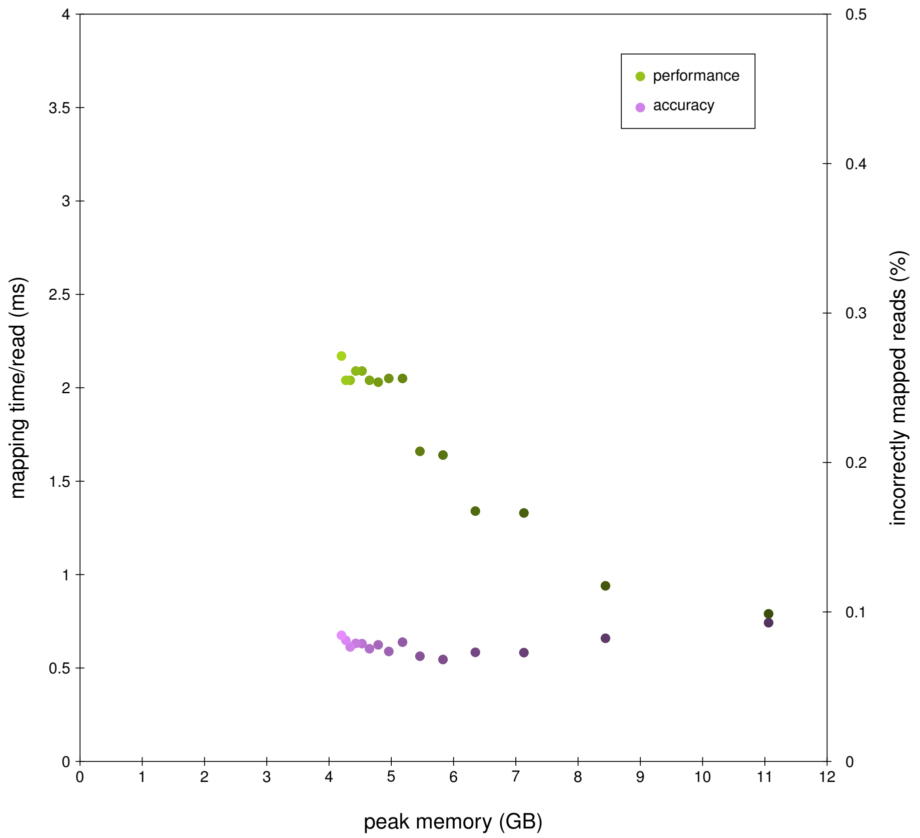

Figure S9: Influence of sparseness when indexing the reference genome with an enhanced sparse suffix array on the memory footprint, execution speed and accuracy of the read mapper ALFALFA. The scatterplot displays the peak memory measured during read mapping for all even sparseness values between 4 and 32, against the corresponding performance and accuracy of the read mapper. For these tests ALFALFA reported a maximum of 4 alignments per read. Green dots (left axis) plot performance as the average time needed to map one read (in milliseconds). Purple dots (right axis) plot accuracy as the percentage of reads for which no alignment was found within 10bp of the simulated origin or with less errors than were simulated. Darker shades of green and purple correspond to lower sparseness values. Evaluation was done using the 600bp read set from the benchmark study that was generated using the Wgsim simulator (Table S1.1). As a reference, the benchmark study evaluates ALFALFA with a sparseness value of 12, corresponding to the fifth dot from the right.

Table S2: comparison of algorithmic design choices

Read mappers are a complex interplay of many algorithms and heuristics that can all influence performance on a different way. Most read mappers use the same general outline, but differ greatly in choice of algorithms, data structures, heuristics and implementation details. To contrast ALFALFA with other read mappers, Table S2 contains a comparison of the algorithmic design choices in ALFALFA and two other read mappers that share many of its algorithms and heuristics: BWA-MEM and CUSHAW3. Note that in addition to these tools, the techniques given in the table have also been used in other read mappers or in similar applications, and the presented list is not meant to be exhaustive. The categories presented in Table S2 more or less follow the steps described in the Supplementary Methods section.

ALFALFA is unique in that it uses an enhanced sparse suffix array as an index structure for fast lookups in the reference genome, whereas other tools rely on the FM-index for the same purpose. The index structure not only provides a different memory-time trade-off for finding seeds [1], but the seed-finding algorithms implemented on this index structure also finds a combination of MEMs and SMEMs that is different from that in other tools. BWA-MEM will find SMEMs with some additional MEMs, whereas CUSHAW3 initially finds MEMs. CUSHAW3 can, however, find fixed length seeds during a rescue procedure. Both ALFALFA and CUSHAW3 tune the minimum seed length depending on the read length and number of errors, whereas BWA-MEM uses a fixed minimum seed length.

All tools define a type of candidate alignment region whose size approximates that of the read. To our understanding, CUSHAW3 defines a region for each seed found. BWA-MEM forms chains of seeds, and filters out small and overlapping chains. In contrast, ALFALFA clusters seeds together into non-overlapping regions, but does not immediately chain the seeds.

ALFALFA does not extend all candidate regions, as is explained in the supplementary methods. In addition, the order of extension is based on the number of bases in the read covered by seeds. BWA-MEM similarly orders the chains based on the read coverage and also on the seed length. BWA-MEM also filters seeds (candidate regions) if the seed is already contained in a previous alignment. We did not find any information on ordering or filtering of candidate regions for CUSHAW3, but it could be assumed that CUSHAW3 extends all candidate alignment regions, as it is designed to be highly accurate.

BWA-MEM and CUSHAW3 both utilize a (optimized) banded Smith-Waterman algorithm to calculate the alignment score and alignment. In contrast, ALFALFA performs a chain-guided alignment which reuses the seeds and only performs a dynamic programming routine to fill the gaps in the chain. Note that both BWA-MEM and ALFALFA utilize the *klib* library [2]. In addition, all tools have several rescue procedures that are invoked at several steps of the mapping procedure to increase accuracy where initial high-performance heuristics have failed.

[2] https://github.com/attractivechaos/klib

Table S2: Comparison of the algorithmic design choices made by the read mappers ALFALFA, BWA-MEM and CUSHAW3. The first column contains several components of the algorithmic design, data structures used, heuristics employed, or implementation details. The values for BWA-MEM and CUSHAW3 were obtained from our interpretation of the original publications [2–4]. Note that this information might therefore not reflect the current state of the algorithms.

	ALFALFA	BWA-MEM	CUSHAW3
index structure	ESSA	FM-index	FM-index
seed-finding algorithm	3 ESSA-based algorithms	[5]	[3]
seeds	MEMs, SMEMs	SMEMs (and MEMs)	MEMs, fixed length seeds, variable length seeds
minimum seed length	variable	fixed	variable
reseeding	lower minimum length	reseed SMEMs	lower minimum length and other seed types
candidate regions	non-overlapping cluster	(overlapping) chain of seeds	based on single seed
region filter	multiple criteria	overlapping chains and previous alignments	unknown
order of region evaluation	read coverage (all seeds)	read coverage (chain) and global seed length	unknown
extend	chain-guided alignment (multiple chains)	banded SW-alignment with optimizations	banded SW-alignment
rescue after extend	yes	unknown	yes
post-processing	chain-guided alignment or banded SW	banded SW with optimizations	banded SW

Command line anatomy of ALFALFA

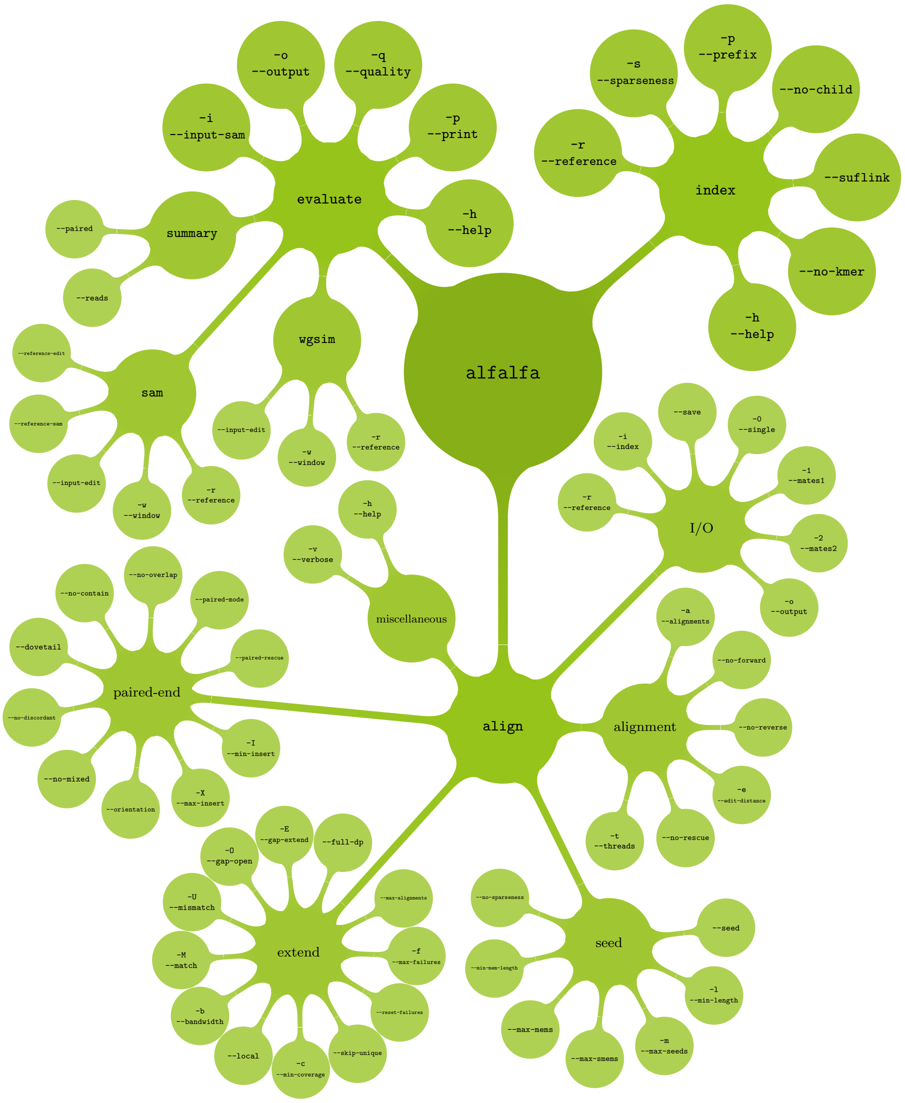

Figure S10: Command line anatomy of ALFALFA. Options can be used to customize the read mapper and are grouped per command, subcommand and category. See Supplementary Data for further details on the options used by ALFALFA.

Supplementary Methods

The ultimate goal set by ALFALFA is to report a number of different *feasible* alignments between the reads and the reference genome. An alignment is deemed feasible if the fraction of differences in the alignment to the read length is smaller than the value set by the parameter `-e`. This fraction multiplied by the read length returns the maximal allowed edit distance for a read alignment. Parameter `-e` is also used to determine when two alignments are considered different, namely when the difference between their mapping positions is less than the maximal allowed edit distance. The number of different alignments returned is set by the parameter `-a`, which is usually much smaller than the actual number of alignments calculated during the mapping process.

To accomplish this goal, ALFALFA employs the seed-and-extend principle which is widely used among read mappers. This principle dictates that the algorithm first searches for short matches between a read and the reference genome, and then extends these *seeds* into full alignments. Although ALFALFA shares its high-level strategy with other read mappers, the low-level implementation of the various phases of the algorithm differs significantly from other mappers. These differences are prominent in the type of seeds that are found, in the way seeds are filtered and combined into candidate regions for extension, in the selection of candidate regions, and in the extension strategy. In addition, ALFALFA applies many new heuristics to optimize its performance for long reads.

The pseudocode in Algorithm 1 shows the high-level single-end alignment strategy followed by ALFALFA. The seeds calculated in line 4 are filtered and grouped together into candidate regions in line 9. After being sorted, these candidate regions are further examined in line 13 to see if they may contain a good alignment. The seeds in the regions that meet the criteria are chained together in line 16, after which the chains are extended in line 17 using dynamic programming. The result of the extension procedure is an alignment score, which is converted into a full alignment in line 28 for the best alignments found.

The various subprocedures in Algorithm 1 are discussed in detail in the next sections. These sections contain a description and the pseudocode of the algorithms and heuristics used, supplemented with examples to illustrate design choices and a discussion on advantages and disadvantages of the methods and viable options for parameter settings, when applicable.

As an example, Figures S11 to S13 depict the flow of the algorithm for one read. Figure S11 shows the reference genome as separate segments (chromosomes or contigs) organized in a circle around a read sequence (light green) in the center. Note that the read sequence is not drawn in scale with the reference genome. The figure also shows the location of all seeds found as black bars around the read (in this case all MEMs with $\ell \geq 30$ were used as seeds). The seeds are also connected to their position in the reference genome. Bars above (below) the read represent seeds located on the forward (reverse) strand. The dots on the reference genome represent regions containing MEMs and are thus candidate mapping locations. In addition, dots in which more lines converge will have a higher score as more MEMs are found in there. In this example, most regions will contain only a single small seed and will thus be filtered out in later phases. Furthermore, the seeds span the entire read sequence only in a single region. This region, identified by the black dot outside the circle, is also the region from which the read was simulated.

Algorithm 1 outline of single-end mapping algorithm used by ALFALFA

Input: reference genome G, list of reads L_R
Output: SAM file containing alignments of reads onto G

```
read input and parse parameter options
construct enhanced sparse suffix array I for G
for all reads R in L_R do
    seeds ← calculateSeeds(G, R, I)
    if no seeds were found then
        lower minimum seed length
        seeds ← calculateSeeds(G, R, I)
    end if
    regions ← identifyCandidateRegions(seeds)
    sort regions according to seed coverage of the read sequence
    while regions remaining and not enough feasible alignments found do
        C ← current region
        if extendRegion(C) then
            sort seeds in C according to length
            for all seed s ∈ C do
                chain ← buildChain(s, C)
                alignment ← extendChain(chain)
                if feasibleAlignment(alignment) then
                    add alignment to R
                end if
            end for
        end if
    end while
    if no feasible alignments found then
        rescueProcedure(G, R, I, regions)
    end if
    sort feasible alignments
    postProcess(R)
end for
```

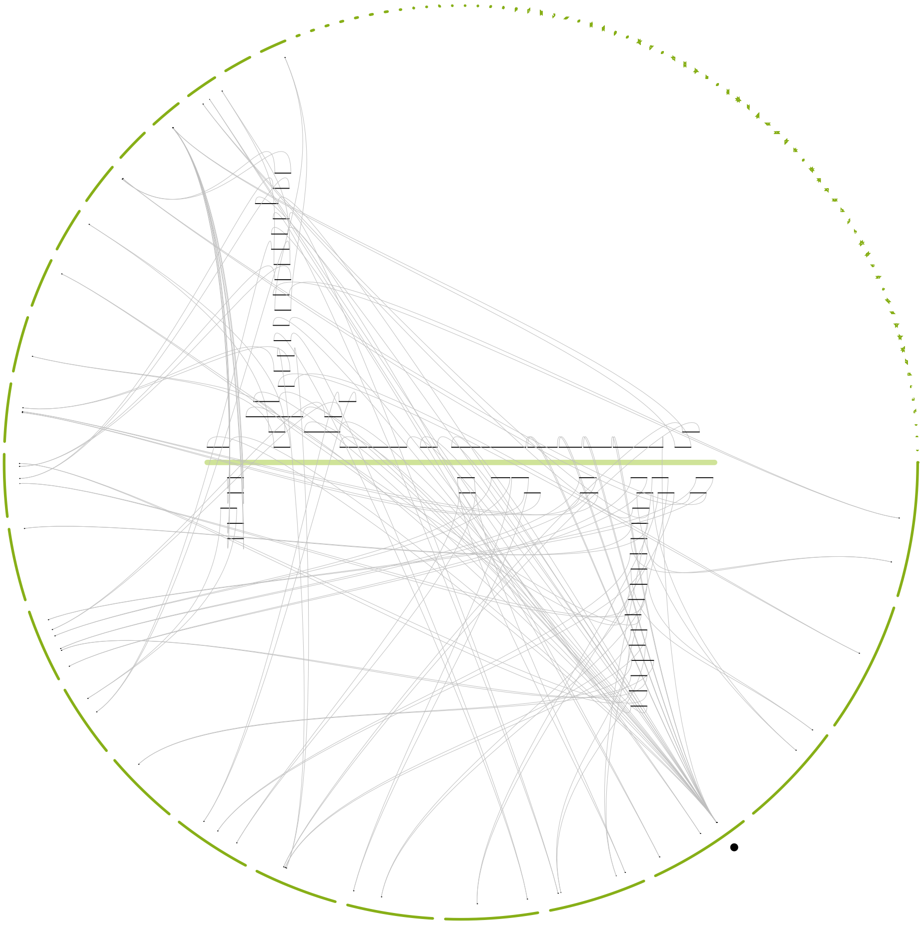

Figure S11: Example illustrating the general workflow of ALFALFA, showing the collection of seeds (MEMs) that are found between a read (light green bar in the center) and a reference genome (green circle, each segment is a chromosome or contig). Seeds are depicted as black bars around the read, on top of the read for seeds found on the forward strand and below the read for seeds found on the reverse strand. The seeds are connected to their location on the reference genome. As a reference, the black dot outside the circle represents the simulated location of the read.

In Figure S12, we have zoomed in to the area around the highest scoring candidate region. The read and reference genome are shown in the same way as in Figure S11, but now the displayed region of the reference genome is small enough to show the exact location of the seeds on the reference genome. The black arc outside the reference genome shows the location of the candidate region as delineated by ALFALFA. This candidate region extends somewhat beyond all seeds to take into account parts of the read that are not covered by seeds and additional insertions. The black segment on the reference genome corresponds to the location from which the read was simulated, thus corresponding to the location of an optimal alignment. The simulated region does not include the first two seeds, but does extend beyond the last seed. As a result, the candidate region identified by ALFALFA completely contains the simulated region.

Finally, Figure S13 illustrates the results of the chaining and alignment phase of the algorithm. The candidate region found by ALFALFA is shown at the top and the read at the bottom. Most read mappers extend candidate regions by performing full dynamic programming within the boundaries of the region. ALFALFA, however, combines seeds in the candidate region into a chain, covering large sections of the matching bases of the alignment. The seeds in the example are depicted as connected bars between the read and reference genome. Seeds making up the chain are shown in black, whereas those not included in the chain are shown in red. Dynamic programming is only performed in between two seeds and at the edges of the candidate region. As a result, large sections of the alignment are already fixed before costly dynamic programming routines are applied. The figure also clearly illustrates the importance of finding a good set of seeds. Ideally, the chain covers as much of the read sequence as possible. To illustrate the quality of the seeds for this example, the grey areas in Figure S13 represent parts of an optimal alignment that consists of three or more consecutive matching bases. Among those, light grey areas are covered by seeds, whereas darker ones are not. In this example, all large matching areas are covered, except some at the end of the read. Most matches that were not found as seeds are due to the lower limit imposed on seeds by ALFALFA during seed-finding.

Enhanced sparse suffix arrays

ALFALFA is the first read mapper that is underpinned by an enhanced sparse suffix array. This fast and low-memory footprint index structure can be used for quick retrieval of maximal exact matches (MEM) and super-maximal exact matches (SMEM) between a reference genome and a set of sequencing reads. The overall design of the index structure and the seed-finding algorithm are both adopted from essaMEM [1].

Enhanced sparse suffix arrays belong to the family of index structures that originate from suffix trees, the basic data structure supporting fast and flexible sequence analysis [6]. Suffix trees enable optimal linear time implementations of most genome analysis algorithms, but suffer from high memory footprints. As an alternative, suffix arrays (SA) [7] can replace suffix trees as a more memory-efficient, but less flexible index structure. To improve flexibility, SAs can be supplemented in a modular way with three additional arrays to form enhanced suffix arrays (ESA) [8]. ESAs reach full suffix tree expressiveness at the cost of additional memory requirements. These memory requirements can be alleviated

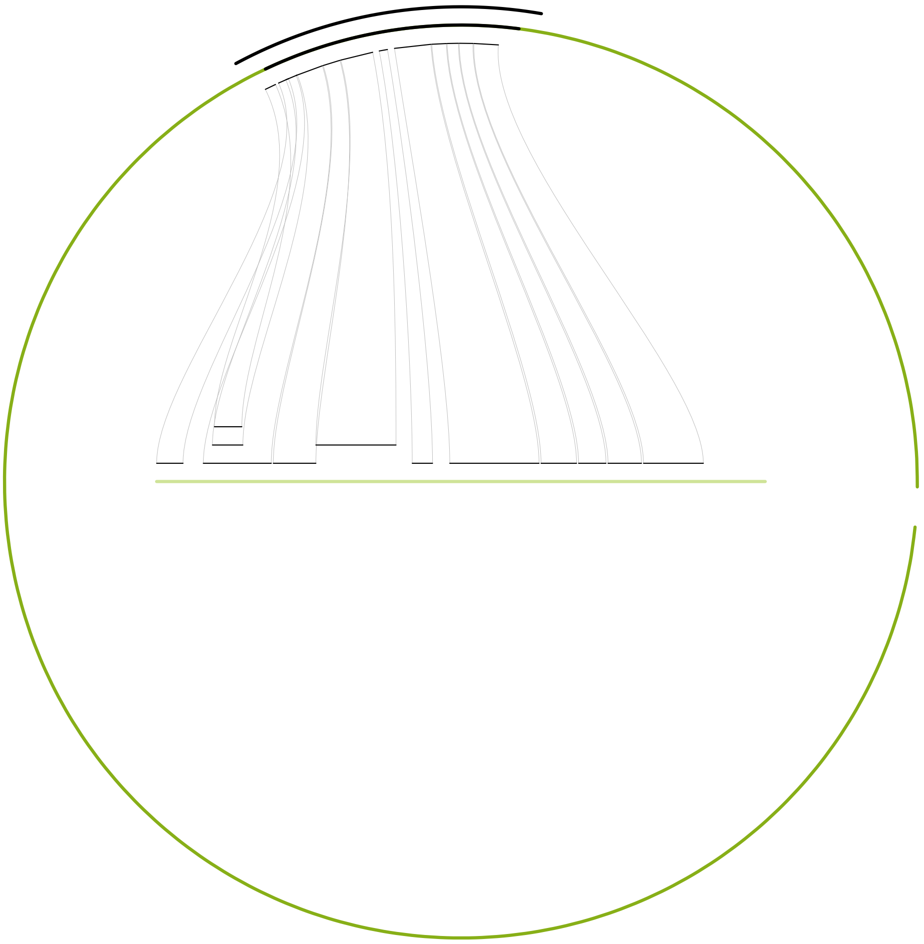

Figure S12: Example illustrating the general workflow of ALFALFA, showing the collection of seeds (MEMs) in a single candidate region. Seeds between the read (light green bar in the center) and the area around the candidate region (green circle) are depicted as connected black lines. The black segment on the reference genome indicates the region of an optimal (simulated) alignment and the black segment outside the circle indicates the part of the reference genome defined by ALFALFA as the candidate alignment region.

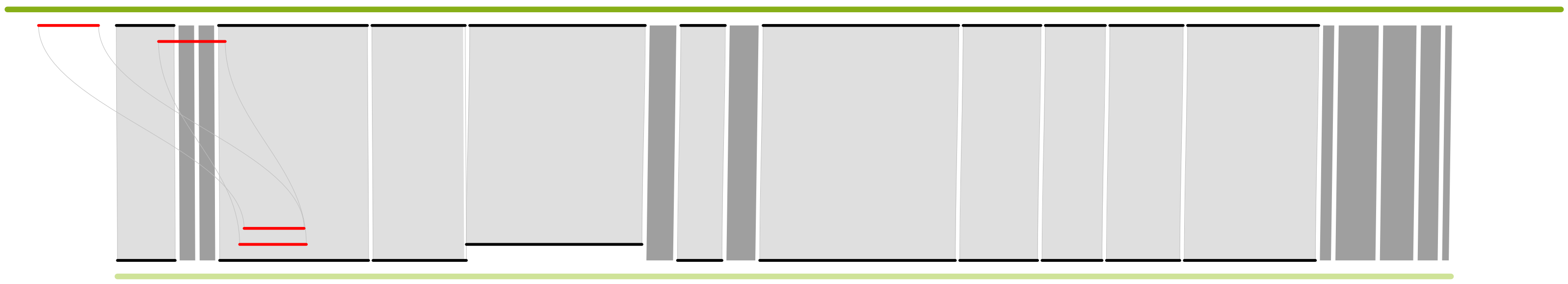

Figure S13: Example illustrating the general workflow of ALFALFA, showing the candidate alignment region found by ALFALFA (top) and the read (bottom), together with the seeds in the candidate region (connected bars). Seeds used in the chain are depicted in black, and seeds that are filtered out are depicted in red. Grey areas show regions in an optimal alignment of three or more consecutive matching bases. Light gray areas are covered by seeds, whereas dark gray areas are found using dynamic programming.

by indexing only a sampled number of suffixes (sparse suffix arrays; SSAs) or compressing the suffix array (compressed suffix arrays; CSAs). Similar to ESAs, enhanced sparse suffix arrays (ESSA) improve flexibility of SSAs by supplementing the basic SA with additional (sparse) data structures. A detailed review of this family of index structures and their applications in genome research can be found elsewhere [9, 10].

Seed-finding

Seed-finding is the first major step in the algorithm that, depending on the data and parameter settings, takes about a fourth to half the runtime of the mapping algorithm. This step also provides a basis for the accuracy and performance of the rest of the algorithm, as seeds are both used for identifying candidate mapping regions and chaining in the final phase of the mapping algorithm. Ideally, a read is fully covered with seeds to achieve good chaining, while few seeds fall outside candidate regions that contain feasible alignments to save CPU cycles on the investigation of spurious candidate regions. ALFALFA provides several parameters to tune the performance of the seed-finding process, each producing different performance-accuracy trade-offs. These parameters are:

- the sparseness of the index (parameter `-s`),
- the sparseness in the read (automatically configured),
- the minimum length of the seeds (parameter `-l`),
- the maximum number of seeds per read offset (parameter `-m`),
- the type of seeds searched for (parameter `--seed`).

An example of the trade-offs obtained by the different settings can be found at the end of this section.

In our benchmark study, the sparseness of the ESSA is set such that the memory footprint of the index is comparable to that of other read mappers. Sparseness in the read means that during seed-finding, offsets in the read are skipped to save time. It is, however, possible to still find all types of seeds such as maximal exact matches. ALFALFA automatically configures this parameter for optimal performance, based on the findings of the essaMEM algorithm. For an ESSA with sparseness s and minimum seed length ℓ, it is set to

$$\text{sparseness read} = \begin{cases} \left\lceil \frac{\ell-10}{s} \right\rceil, & \text{if } s \geq 4 \\ \left\lceil \frac{\ell-12}{s} \right\rceil, & \text{if } s < 4 \end{cases}$$

ALFALFA finds variable-length seeds with a fixed minimum length, which is 40 by default. The default was chosen, based on our experiments for the human reference genome and varying read lengths. For shorter genomes, this minimum length could be lowered to increase sensitivity of the algorithm. On the other hand, ALFALFA also automatically tunes the minimum seed length for reads longer than 1000bp. For those reads, the minimum length ℓ is incremented by 20 for every 500bp above 1kbp, with the total increment being divided by the maximum percentage of errors allowed in accepting alignments.

In addition to tuning minimal seed length, ALFALFA does not take into account seeds that appear too frequently (parameter `-m`). We say that a seed appears *multiple times* if a seed on the read sequence occurs multiple times in the reference genome. By default, ALFALFA also does not take into account a set of matches sharing the same length and offset in the read if the set contains more than $10,000$ seeds. Highly repetitive matches are unlikely to lead to unique alignments and can thus be safely omitted. Moreover, seeds that appear with a very high frequency unnecessarily increase computational effort of the candidate region selection phase.

ALFALFA uses maximal exact matches as seeds. There are, however, three different seed-finding algorithms implemented that produce different sets of MEMs. These algorithms are described in the following subsections.

Maximal exact matches

A maximal exact match (MEM) between two strings is an exact match that cannot be extended to the left or right without inducing a mismatch. This means that MEMs are variable-length seeds that are locally as long as possible, making them good candidates for seeds in (long) read mapping algorithms [2,3] and sequence alignment [11]. Formally, MEMs can be defined as follows:

Definition 1 *The triplet (i, j, ℓ) is called a maximal exact match (MEM) between two strings S and T of size $|S| = n$ and $|T| = m$ if:*

- $S[i..i + \ell - 1] = T[j..j + \ell - 1]$
- $i = 0$ *or* $j = 0$ *or* $S[i - 1] \neq T[j - 1]$
- $i = n - 1$ *or* $j = m - 1$ *or* $S[i + \ell] \neq T[j + \ell]$

The second and third condition are respectively called the left-maximality and right-maximality condition. If only the first two conditions hold, the pair is called a left-maximal exact match. Likewise if only the first and third condition hold, the pair is called a right-maximal exact match.

Algorithm 2 finds all MEMs of a given minimum length between a read and a reference genome using an ESSA index. In essence, the algorithm is very intuitive if no sparseness is present in the index structure nor in the read. In that case, the algorithm loops over all suffixes of the read in line 6 and matches each of those suffixes to the index until a mismatch is found. Index structures such as suffix arrays speed up the first and most time-consuming step of this algorithm in grouping together the suffixes of the reference genome by common prefix in a conceptual search tree data structure (Figure 1a). A single read suffix can thus be matched simultaneously against all indexed suffixes of the reference genome. Navigating the conceptual tree data structure requires either a logarithmic binary search algorithm (SA) or a constant time algorithm using information from additional arrays (ESA). At each branching point in the search tree, mismatches are found between the current matched substring of the read and a set of substrings in the reference genome, resulting in identification of right-maximality. All branching points starting from a depth equalling the minimum length are saved (line 7). At line 10, all right-maximal exact matches

Algorithm 2 MEM-finding algorithm

Input: reference genome G, ESSA index I and read sequence R
Output: all MEMs between G and R of minimum length ℓ

```
ℓ ← MINIMUMLENGTH(|R|,e) // minimum similarity = 100 − e
s ← sparseness of I
p ← SPARSENESS(ℓ,s) // p is the sparseness in the read
// ℓ_s is the minimum length of right-maximal exact matches
ℓ_s ← ℓ − (p · s − 1)
for i in [0..s − 1] do
    for j in [i..|R| − ℓ_s] do
        mli ← match R[j..j + ℓ_s − 1] to I
        if # characters matched ≥ ℓ_s then
            xmi ← match P[j + ℓ_s − 1..] to I within interval mli
            // xmi contains the longest right-maximal exact matches
            // xmi is a subinterval of mli
            for all right-maximal exact matches in xmi do
                compare up to s · p characters to check for left-maximality
                if length of match is at least ℓ then
                    report match
                end if
            end for
            // find progressively smaller MEMs by expanding xmi to mli
            while |xmi| < |mli| do
                expand xmi until smaller right-maximal matches are found
                for all right-maximal exact matches added values to xmi do
                    compare up to s · p characters to check for left-maximality
                    if length of match is at least ℓ then
                        report match as MEM
                    end if
                end for
            end while
        end if
        j ← j + s · p
    end for
end for
```

have been found and the remainder of the algorithm consists only of comparing the bases upstream of the matching positions to check for left-maximality.

The introduction of sparseness by indexing only one in every s suffixes of the reference genome does not lead to information loss if the sparseness factor is less than the minimum length imposed on MEMs. Moreover, it only requires minor adjustments to the algorithm as described above. Due to the properties of suffixes, MEMs starting at unsampled suffixes in the reference genome can be retrieved by (a) matching the first sampled suffix downstream in the reference genome and the corresponding suffix of the sequencing read (located within $s-1$ positions of the complete MEM starting position) and (b) comparing up to s bases upstream of the matched position to find the starting position of the complete MEM and to check for left-maximality. These alterations only require some extra CPU cycles during the second phase of the algorithm, which in practice does not result in a significant performance drop [1].

Analogously to sampling suffixes of the reference genome, it is also possible to match only a sampled set of the suffixes of the sequencing read during the first phase of the algorithm. By doing so, fewer executions of the time-consuming matching algorithm are required. Moreover, nothing prevents combining these sparse suffix sampling schemes of both the reference genome and the sequencing read, resulting in a reduced memory footprint of the index structure and a performance boost of the MEM-finding process. The only requirement for a successful combination is the use of two asynchronous sampling schemes, since equidistant samplings could cause matches with shifted periods not to share any corresponding suffixes. In addition to sampling every s-th suffix of the reference genome, we therefore sample s consecutive suffixes of the sequencing read for every block of $s \cdot p$ suffixes, with skip factor p indicating the sparseness of suffix sampling in the read. Figure S14 shows an example of this approach with $s = 2$ and $p = 2$. For a given MEM, all $s \cdot p^2 = 8$ possible strides of both samplings are given. In each case, it is possible to find a combination of sampled suffixes that have the same offset from the actual starting position of the MEM. Because the maximal offset is $s \cdot k$, the product of the sparseness parameters is restricted to the minimal length ℓ of the MEMs. In practice, this upper bound should be lowered by a constant, as the first phase of the MEM-finding algorithm generates all right-maximal exact matches of minimum length $\ell - s \cdot p$.

Theoretically, Algorithm 2 has a time complexity of $\mathcal{O}m^2 + |\textit{right MEMs}|$, with m the length of the read and $|\textit{right MEMs}|$ the number of right-maximal exact matches. In practice, the algorithm has proven to be very fast in finding all MEMs between two large genome sequences [1].

Super-maximal exact matches

For long read mapping, Algorithm 2 is rather slow and produces a large set of seeds, which hinders fast identification of a small set of candidate mapping locations. As a result, ALFALFA implements two other seed-finding algorithms that find smaller subsets of MEMs which have interesting properties for MEM-finding. The first algorithm finds (a subset of) super-maximal exact matches (SMEMs), which were first introduced for the *de novo* genome assembler *fermi* [5] and are also used in the read mapper BWA-MEM. A super-maximal exact match between a read and reference genome is a MEM that is not contained in another MEM in the read. The use of SMEMs as seeds is

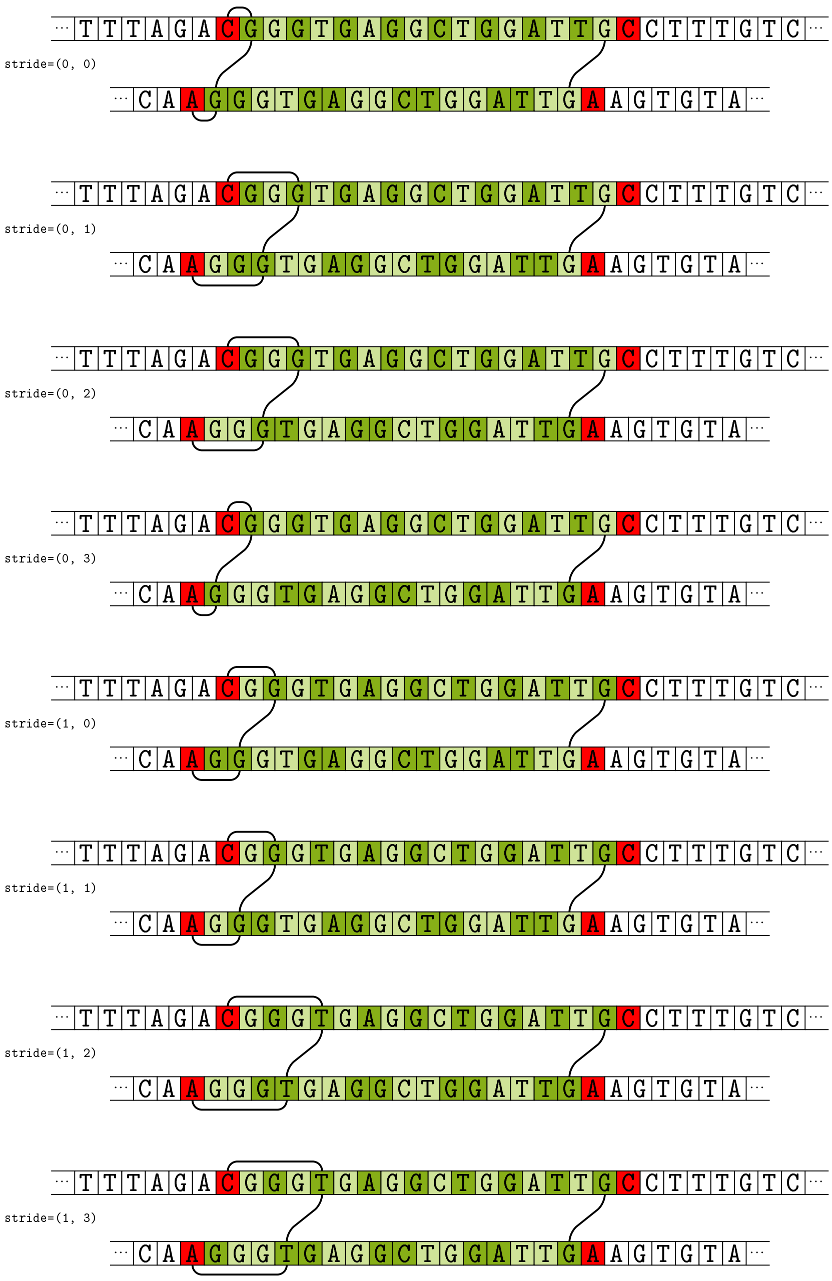

TTTAGACGGGTGAGGCTGGATTGCCTTTGTC
stride=(0, 0)
CAAGGGTGAGGCTGGATTGAAGTGTA
stride=(0, 1)
stride=(0, 2)
stride=(0, 3)
stride=(1, 0)
stride=(1, 1)
stride=(1, 2)
stride=(1, 3)

Figure S14: *(previous page)* All 8 possible cases that might occur during MEM-finding between a reference genome (top) indexed with sparseness $s = 2$, and a read sequence (bottom) with skip factor $p = 2$. The matching subsequence `GGGTGAGGCTGGATTG` (green) is maximal as it is flanked by mismatching base pairs `C` and `A` (red). Initial matches can only be found between two sampled suffixes, whose starting positions are indicated in dark green. Light green bases indicate starting positions of suffixes that are not sampled. With $s = 2$ and $p = 2$, every second suffix is indexed and $s \cdot (p-1)$ consecutive blocks of suffixes are skipped for every successive block of s suffixes that are matched in the read. The right-maximal exact match (vertical connection lines) that is found by the algorithm is a suffix of the complete MEM, whose starting position depends on the stride of the sampled suffix blocks. Horizontal connection lines show the prefix of the right-maximal exact match that needs to be additionally inspected in order to find the left border of the complete MEM. The length of this prefix is at most $s \cdot p$.

motivated by the fact that the MEM with the highest similarity to a part of the reference genome, i.e. the longest, will most likely be found in a region containing a good alignment between read and reference genome. As an example, Figure S15 shows all MEMs of minimum length 5 between two sequences. There are 4 MEMs in total, of which 3 are SMEMs. Only the third (with offset 19 in both sequences) is contained in the SMEM with offsets at $S[30]$ and $P[19]$.

Algorithm 3 is designed to find a smaller set of MEMs, of which most are SMEMs. Furthermore, it is guaranteed to find all SMEMs if there is no sparseness in the read or index of the reference genome. Technically, the algorithm is highly similar to the MEM-finding algorithm given in Algorithm 2. The main difference is that Algorithm 3 does not keep track of all right-maximal matches longer than the minimum length. Given an offset in the read, left-maximality is only checked for the longest right-maximal exact matches.

If the sparseness s of the ESSA and the sparseness p in the read are equal to one (no sparseness), the set of MEMs returned is exactly the set of all SMEMs. To see this, suppose that $m = (i, j, \ell)$ is an SMEM that corresponds to the substring $S[i..i + \ell - 1]$ in the reference genome S and $P[j..j + \ell - 1]$ in the read P. If there is no sparseness, the algorithm loops over all offsets of P. For offset j, the algorithm will match exactly ℓ characters because *i)* at least ℓ characters can be matched due to $P[j..j + \ell - 1] = S[i..i + \ell - 1]$ and *ii)* no more than ℓ characters can be matched due to m being an SMEM. Similarly, the algorithm will not find any right-maximal match with offset j and length ℓ that is not left-maximal, because otherwise a longer MEM could be found that contains m. As a result, the condition in line 12 has been met and m is reported.

Conversely, if the algorithm reports a MEM $m = (i, j, \ell)$, it is an SMEM. No other MEM matched $P[j..j + \ell]$ because it did not match any substring of S (line 7). As a result, there is no other MEM that covers $P[j..j + \ell - 1]$ and extends to the right of it. Similarly, if there would be a MEM covering at least $P[j..j + \ell - 1]$ and extending to the left of it, this match would be found as a right-maximal exact match together with m, but it would not be left-maximal. In that case, the condition in line 12 would be false and m would not be reported. Therefore, no other MEM can be found that is longer than ℓ

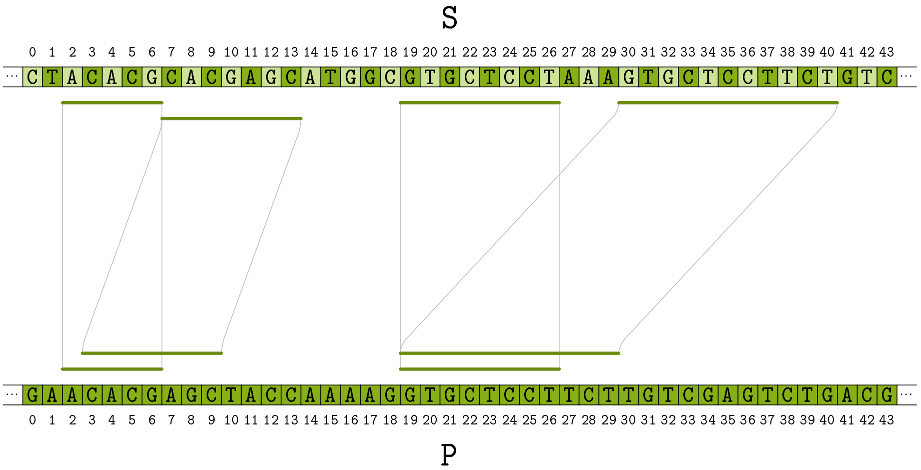

Figure S15: MEMs of minimum length 5 between reference genome S and read P. Among the MEMs, only the one of length 8 with offset 19 in both S and P (third from the left) is not an SMEM, as it is contained in a larger MEM. In addition to the MEMs, the figure shows the sampling of S in an ESSA index structure with sparseness 2. Only suffixes that start at dark green characters are sampled in the index.

Algorithm 3 SMEM-finding algorithm

Input: reference genome G, ESSA index I and read sequence R
Output: a set of SMEMs between G and R

```
ℓ ← MINIMUMLENGTH(|P|,e) // minimum similarity = 100 − e
s ← sparseness of I
p ← SPARSENESS(ℓ,s) // p is the sparseness in the read
// ℓ_s is the minimum length of right-maximal exact matches
ℓ_s ← ℓ − (p · s − 1)
for all i in range [0..s − 1] do
    for j from i to |R| − ℓ_s do
        xmi ← match R[j..] to I
        if # characters matched ≥ ℓ_s then
            // xmi contains the longest right-maximal exact matches
            for all right-maximal exact matches in xmi do
                compare up to s · p characters to check for left-maximality
            end for
            if max(s, p) = 1 and all matches in xmi are MEMs then
                report all matches in xmi as SMEMs
            else if max(s, p) > 1 then
                ℓ_min = ℓ − s
                report MEMs in xmi whose length is at least ℓ_min
            end if
        end if
        j ← j + s · p
    end for
end for
```

Algorithm 4 MEM and SMEM-finding algorithm

Input: reference genome G, ESSA index I and read sequence R
Output: a set of MEMs between G and R

    // *define variables similar to Algorithm 2*
1: $\ell \leftarrow$ MINIMUMLENGTH($|R|$,e)
2: $s \leftarrow$ sparseness of I
3: $p \leftarrow$ SPARSENESS(ℓ,s) // *p is the sparseness in the read*
4: $\ell_s \leftarrow \ell - (p \cdot s - 1)$
5: *intervals* $\leftarrow$ empty list
6: **for all** sampled positions i in R **do**
7:     calculate *mli* and *xmi* in a similar way to Algorithm 2
8:     *intervals*$[i] \leftarrow (i, mli, xmi)$
9: **end for**
10: sort *intervals* in descending order of rightmost matched position in R
11: *smems* $\leftarrow$ empty dictionary
12: **for all** (i, mli, xmi) in *intervals* **do**
13:     $d \leftarrow$ length of right-maximal match in *xmi*
14:     **if** $[i..i + d]$ is not contained in *smems* **then**
15:         **if** $|xmi| \leq$ EXT **then** // *EXT is a parameter, default 10*
16:             report all mems up to *mli*
17:         **else**
18:             report all mems from *xmi*
19:         **end if**
20:         update *smems* dictionary
21:     **end if**
22: **end for**

characters and in which m is fully contained.

By default, ALFALFA induces sparseness in both the read and reference genome. In this case, Algorithm 3 is not guaranteed to produce the set of all SMEMs. An example of an SMEM that is not reported and a reported MEM that is not an SMEM are shown in Figure S15. The SMEM $m = (2, 2, 5)$ covering $S[2..6]$ and $P[2..6]$ is not found. The suffix $S[2..]$ is not sampled and therefore m cannot be found if the offset in P is 2. For offset 3, m would be reported by Algorithm 2 because it is a MEM. For offset 3, however, Algorithm 3 finds a longer right-maximal exact match $(7, 3, 7)$. It is therefore unable to find m. Although the MEM $m' = (19, 19, 8)$ is contained in the longer MEM $m'' = (30, 19, 11)$, and is thus not an SMEM, it will be reported. This is because suffix $S[30..]$ is not sampled and thus m'' is not found at offset 19 in P but in offset 20.

Although Algorithm 3 does not have the nice theoretical property of guaranteeing to find all SMEMs, we have found that the set of seeds produced by the algorithm is sufficient for our purposes. In a post-processing step, it is easy to filter out all SMEMs from the data set returned by Algorithm 3. In addition, the larger data set allows ALFALFA to build larger chains for use in the extension phase of the mapping algorithm. For this reason and to somewhat counter the effect of sparseness, we have decreased the minimum seed length requirements in line 15.

Table 3: Performance and accuracy results for ALFALFA using different seed-finding settings on a data set of wgsim simulated reads with average length 600bp. ALFALFA was configured to return a maximum of 4 alignments per read. A comparison of the performance against other mappers with this data set can be found in Table S1.1. The settings vary in type of seeds and minimum seed length used.

setting	runtime (h:mm)	accuracy (%)	recall (%)
MEM, $\ell = 40$	1:05	99.937	99.783
SMEM, $\ell = 40$	0:14	99.931	99.777
PSMEM, $\ell = 40$	0:19	99.933	99.781
SMEM, $\ell = 30$	0:22	99.935	99.782
SMEM, $\ell = 50$	0:10	99.913	99.762

From our experiments, we found that the reduced set of SMEMs is ideal for identifying a small set of candidate regions, but was sometimes too small to build large chains to be used in the extension phase of the algorithm. Algorithm 4 is inspired by the seed-finding procedure in BWA-MEM and is a combination of previous algorithms in that it will produce more MEMs in regions in which few SMEMs are found. The algorithm first proceeds as in Algorithm 2 by finding all right-maximal exact matches of a given minimal seed length. It then sorts the intervals of right-maximal exact matches in descending order of rightmost matched position in the read. This sort procedure is used to filter out intervals of right-maximal exact matches that will not be SMEMs in line 14. Finally, the algorithm either finds all MEMs as in Algorithm 2 or only the longest MEMs for an offset in P as in Algorithm 3 based on the size of the latter data set (condition in line 15).

Comparison of seed-finding settings

The choice of parameter settings and seed-finding algorithms not only has an impact on the performance of the seed-finding phase, but also affects the remainder of the mapping algorithm. Table 3 shows the mapping results for the three algorithms given above with fixed minimum length 40 and two additional settings using the default SMEM algorithm with lower and higher minimum lengths. In Table 3, Algorithm 4 is reported as PSMEM. The results show that the use of all MEMs as seeds achieves the highest recall rate, at the cost of the runtime, which is three to four times higher than that of the other methods. The SMEM algorithm, which is the default setting, achieves the best runtime, and is only slightly less accurate. With a few more reads mapped correctly and a slightly higher runtime, the trade-off achieved by the PSMEM algorithm (Algorithm 4) is similar to that of the SMEM algorithm.

The results for different minimum length settings in Table 3 are as one would expect. A higher minimum length results in a lower runtime, but misses some alignments due to the lower number of seeds. In contrast, a lower minimum length results in more seeds, which in turns results in a higher chance of finding a good alignment at the cost of increased runtime.

As an example of the impact of the different seed-finding strategies and

parameter settings on the progress of the algorithm, Figures S16 and S17 show possible changes in the example shown Figures S11 to S13. Figure S16 depicts the situation of a high number of seeds resulting from a low minimum seed length setting. Compared to Figure S11, a lot more candidate regions will be identified and analyzed, resulting in a higher runtime. In general, finding more seeds will increase the runtime of all phases of the algorithm, while increasing accuracy. In some cases, however, an abundancy of seeds may lead to some alignments being missed, as the algorithm might not be able to distinguish good candidate regions or be unable to form a good chain from the seeds in a region. In contrast, Figure S17 shows the most common effect of a low number of seeds. The algorithm will usually still be able to recover all candidate regions containing optimal alignments, but the chain will be made up of fewer seeds. This will increase the runtime of the extension phase of the algorithm.

Overall, the trade-offs obtained by the different settings in Table 3 do not vary greatly. This is probably due to the many heuristics employed in the remainder of the algorithm that are designed to cope with a wide array of (badly chosen) parameter settings. One of these heuristics is a rescue procedure that is used when no seeds were found. This rescue procedure will lower the minimum seed length twice in an attempt to find seeds. If no seeds were found after this procedure, the algorithm skips to the next read.

Candidate regions

ALFALFA groups the seeds found in the previous phase of the algorithm into candidate regions of roughly the size of the read length. These candidate regions are subsequently sorted and explored further if they meet certain criteria. This part of the algorithm corresponds to lines 9-13 and 25 in Algorithm 1.

Candidate region identification

Seed-finding in ALFALFA serves two goals, namely *i)* identifying few candidate regions that have a high likelyhood of containing high-scoring alignments and *ii)* creating collinear chains that cover the entire read. Algorithm 5 takes both goals into consideration when identifying candidate regions. The algorithm first filters the set of seeds to only the longest and rarest seeds, which serves the first goal. Candidate regions are then identified using the filtered list. Afterwards, however, seeds that were filtered out are merged with existing candidate regions and reused during chain building.

In detail, Algorithm 5 filters out all SMEMs appearing more than 10000 times in the reference genome (parameter `-m`), and all MEMs that are not SMEMs if they are either shorter than 50bp (parameter `--min-mem-len`) or appear more than 20 times in the reference genome (parameter `--max-mems`). The parameters employed are similar to those used in the seed-finding Algorithm 4. Filtering the MEMs this way significantly reduces the number of candidate regions and thus the runtime of the algorithm. As an example, we ran ALFALFA on the wgsim simulated data set with 600bp reads (see Table S1.1) using the unfiltered set of seeds. For this test case, the number of candidate regions increased ten-fold and the runtime was five times higher than the runtime of the algorithm that used the filtered list.

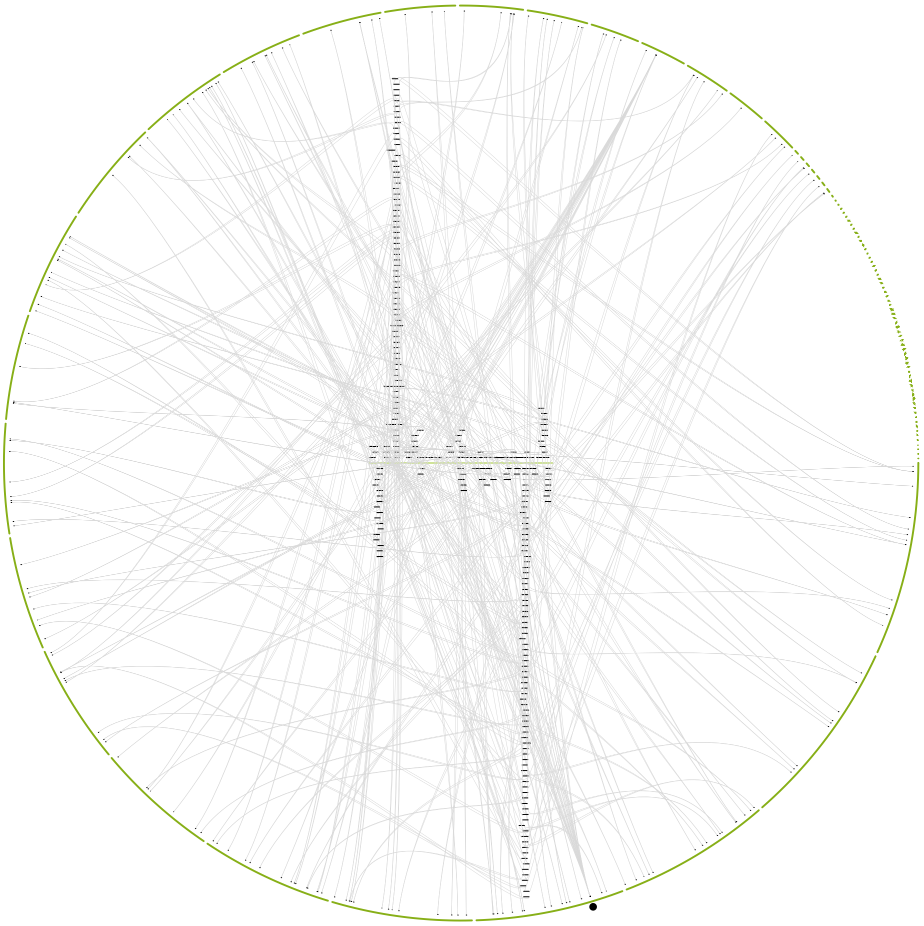

Figure S16: Example illustrating the effect of too many seeds found in the read in Figure S11, showing the collection of seeds (MEMs) that are found between a read (light green bar in the center) and a reference genome (green circle, each segment is a chromosome or contig). The seeds are depicted as black bars around the read, on top of the read for seeds found on the forward strand and below the read for seeds found on the reverse strand. The seeds are connected to their location on the reference genome. The black dot outside the circle represents the simulated location of this read.

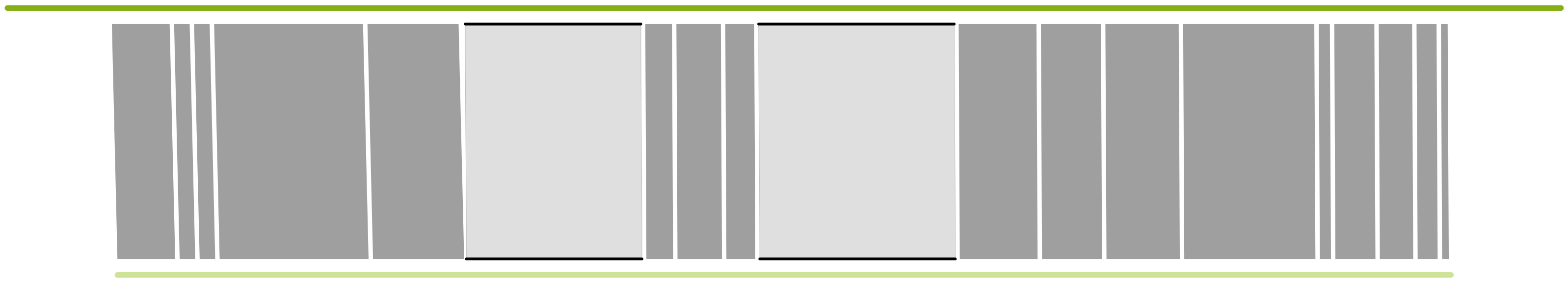

Figure S17: Example illustrating the effect of too few seeds in the read in Figure S13, showing the candidate alignment region found by ALFALFA (top) and the read (bottom), together with the seeds in the candidate region (connected bars). Seeds used in the chain are depicted in black, and seeds that are filtered out are depicted in red. Grey areas show regions in an optimal alignment of three or more consecutive matching bases. Only few areas are covered by seeds (light grey areas), whereas many areas are found using dynamic programming (dark grey areas).

Algorithm 5 Identify candidate regions

Input: set of MEMs and SMEMs *seeds* between reference genome G and read R
Output: list of candidate regions made up from a subset of the seeds

topSeeds $\leftarrow$ empty list
reserve $\leftarrow$ empty list
diff $\leftarrow$ maximum allowed differences
// identify seeds used for candidate region identification
for all s in *seeds* **do**
  if (s is an SMEM **and** appears fewer than $10,000$ times) **or** (s appears fewer than 20 times **and** is at least 50 bases long) **then**
    add s to *topSeeds*
  else
    add s to *reserve*
  end if
end for
sort *topSeeds* according to offset in the reference genome
// identify candidate regions
for all s in *topSeeds* **do**
  $i \leftarrow$ offset of s in G
  $j \leftarrow$ offset of s in R
  $\ell \leftarrow$ length of s
  $C \leftarrow$ current candidate region
  if $G[i..i + \ell - 1]$ falls within C **then**
    if $\ell >$ longest seed in C **then** *// recalculate boundaries of C*
      left bound of $C \leftarrow i - j - \mathit{diff}$
      right bound of $C \leftarrow i - j + |R| + \mathit{diff}$
    end if
    add s to C
    if C now overlaps other regions **then**
      merge C with other overlapping regions
    end if
  else
    start new candidate region with s
  end if
end for
// merge other seeds with existing candidate regions
for all s in *reserve* **do**
  if s can be fit in existing region **then**
    add s to existing region
  end if
end for

The filtered list of seeds is then sorted by offset in the reference genome and traversed left-to-right to find candidate regions. Candidate region boundaries are formed by extrapolating seed boundaries to cover the entire read, supplemented with additional bases to take into account gaps in the alignment. ALFALFA adds seeds to a candidate region as long as they fit the extrapolated boundaries.

Candidate region boundaries are recalculated each time a seed is added that is longer than the longest seed already included in the region. This is based on the idea that longer seeds are more reliable in identifying a candidate region. After recalibration of the candidate region boundaries, ALFALFA checks if the region would overlap the previous one. If this is the case, both regions are merged.

Merging of overlapping regions is performed to avoid possible loss of information in repeated regions. As an example, Figure S18 shows a situation in which parts of the read contain tandem repeats. The black arc on the reference genome (green circle) represents the location of an optimal alignment. Most seeds fall within this region, but several seeds were also found preceding this region. Due to the left-to-right nature of the candidate region identification, a first candidate region is started from the seeds preceding the optimal alignment region. This region extends to halfway the optimal region, after which a second candidate region is started. Both candidate regions cover only part of the optimal alignment region and would lead to sub-optimal alignments. Using the merging algorithm, however, a single candidate region is created in which the optimal alignment is found.

After candidate region identification, seeds filtered out at the beginning of Algorithm 5 are merged back into the candidate regions. The set of all seeds making up a region will be used to rank the region and to eventually build the chain. The extra seeds help to avoid situations such as that of Figure S17 in which the extension procedure is almost as costly as full dynamic programming.

Candidate region selection

After candidate region identification, the resulting list of regions is sorted according to the percentage of bases in the read that are covered by at least one seed. The coverage can be easily computed by traversing the list of seeds that is sorted by start position in the read and calculating the overlap between two consecutive seeds in the list. This second sort does not require additional computational resources as it is mandatory for the chaining algorithm in the extension phase of the algorithm.

In previous experiments, we also tested sorting candidate regions according to the sum of the lengths of the seeds making up the candidate region, but this advantaged adversely repetitive regions in the reference genome.

The list of candidate regions is then traversed in sorted order and candidate regions are extended until a maximum number of feasible alignments have been found (line 1.11). By default, ALFALFA searches for a maximum of 5000 feasible alignments meeting a minimum score. This number can be much higher than the number of reported alignments (parameter `-a`), as the optimal alignment might not be the first feasible alignment found. This setting can be changed using the parameter `--max-alignments`.

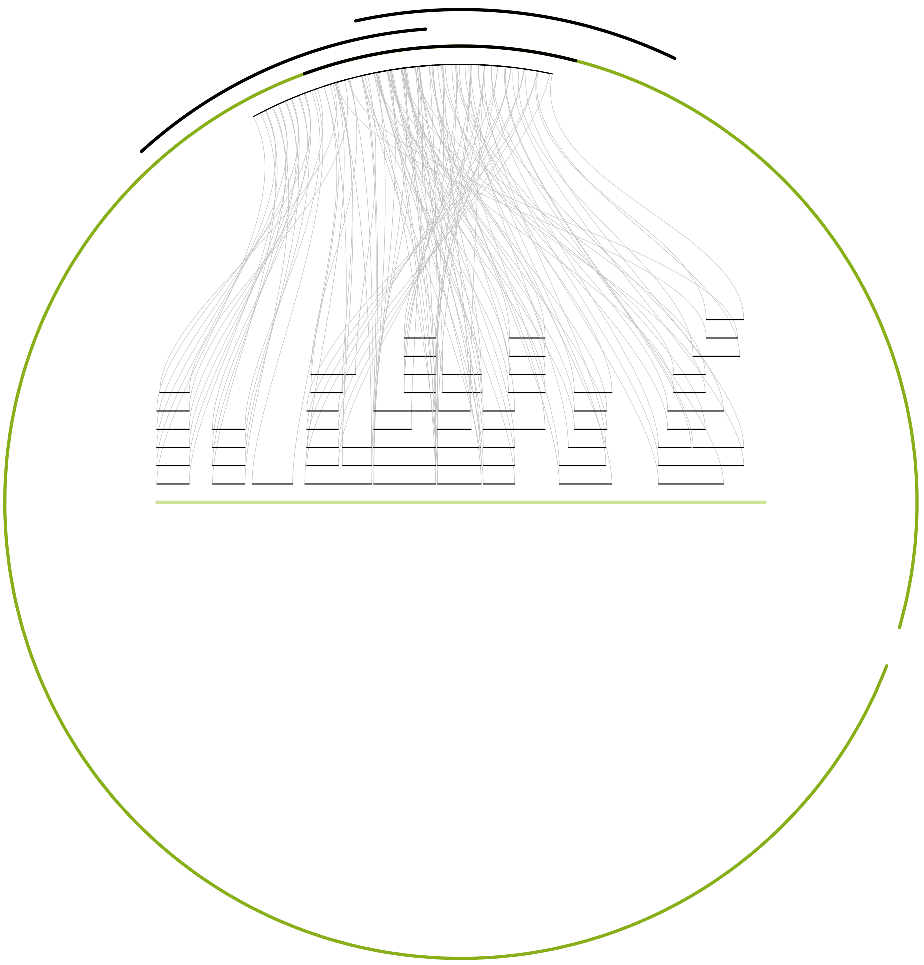

Figure S18: Example of a problem that could arise if overlapping candidate regions would be allowed. The figure represents the read in the center, a region of the reference genome around two candidate regions as the green circle and seeds between the reference genome and the read as connected black bars. The black line on the reference genome represents the region in which an optimal alignment of the read can be found and the two arcs outside the circle represent candidate regions found by ALFALFA. Both candidate regions overlap the region containing an optimal alignment, but not fully contain it. As both regions overlap, ALFALFA will merge them.

For a candidate region to be extended (line 1.13), the following two criteria must be satisfied:

- the region contains at least 2 seeds **or** the read coverage is higher than c,
- fewer than f candidate regions have been extended that did not produce a feasible alignment **or** the region contains a unique seed.

The first extension criterion increases performance of the algorithm by limiting the extension of candidate regions to those regions that are supported by at least two seeds or one long seed. The minimum coverage c is set by the parameter `-c` and is 25% by default.

The first part of the second extension criterion halts the current stage of the algorithm when previously analyzed regions showing good coverage do not produce feasible alignments. The value of f is set by the parameter `-f`, which is set to 10 by default. In contrast, if every candidate region produces at least one feasible alignment, the extension of regions is continued. Furthermore, the bad candidate region counter is reset if a region produces an alignment with a new best alignment score. In addition to continued analysis of successive candidate regions that were successful, a region is always extended if it contains a seed that covers an area of the read that was not previously covered by higher ranked candidate regions.

Rescue procedures

If ALFALFA fails to produce at least one alignment meeting the minimum score set by the user, it can start a two-staged rescue procedure (line 1.25). The first stage reiterates over the list of candidate regions using a significantly lower minimum score for the threshold and minimum coverage requirements (parameter `-c`). As a compensation, the maximum number of alignments calculated per read is lowered (parameter `--max-alignments`). Failure of this rescue procedure is most likely due to bad seed-finding or candidate region identification. Therefore, a second rescue procedure first invokes the seed-finding rescue procedure using less stringent parameter settings, followed by another round of candidate region identification along the same lines as the first rescue procedure.

Rescue procedures are turned on by default. However, it is possible to disallow rescuing using the `--no-rescue` parameter. As expected, the rescue procedures negatively affect the runtime of ALFALFA, but the effect was never significant in all of our experiments. Furthermore, sensitivity of ALFALFA significantly increases using the rescue procedures at the cost of some false positives.

Candidate region extension

This section provides details about the extension phase of the seed-and-extend algorithm (lines 14 to 21 in Algorithm 1). In ALFALFA, candidate mapping regions are linked to the seeds that were used to delimit the region. These seeds are reused in collinear chains, which serve as partial alignments of the read in the candidate region. Full alignments are obtained by applying dynamic programming procedures in between the gaps of the chains.

Algorithm 6 Collinear chaining

Input: reference genome G, read R, list of *seeds* in a candidate region and one seed a used as anchor
Output: *chain* of seeds collinear to a

```
function DISTANCE(G,s1,s2)
    return outer distance between intervals s1 and s2 in sequence G
end function

e ← preset maximum allowed percentage of differences  // parameter -e
add a to chain
sort seeds according to offset in R
p ← a
for all s in seeds to the right of a do
    sDist ← DISTANCE(G,p,a)
    pDist ← DISTANCE(R,p,a)
    skew ← |sDist − pDist|
    if overlap between s and p < 10 and skew ≤ e·max(sDist, pDist) then
        add s to chain
        prevSeed ← s
    end if
end for
prevSeed ← a
for all s in seeds to the left of a do
    ... // same procedure as for seeds to the right of a
end for
```

Chaining

The greedy procedure used by ALFALFA to form collinear chains of seeds is given in Algorithm 6. The algorithm starts from the list of seeds found in the candidate region, sorted by offset in the read, and one seed designated as the *anchor* for the alignment. Starting from the anchor seed, the algorithm extends the chain to the left and right by adding seeds that do not exhibit a large *skew* to the previous seed in the chain and do not overlap with it more than 10 bases.

The *skew* between two seeds is defined as the absolute difference of the distances between the substrings in the read and the reference genome. For example, if the distance between the substrings in the read is 50bp and the distance between the substrings in the reference genome is 70bp, the skew is 20. In this example, a chain consisting of those two seeds would lead to a large deletion in the alignment, which is not common in practice.

An example motivating the use of restricted skew between two seeds is given in Figure S19. Grey areas in the figure represent sequences of more than three matches in an optimal alignment between the reference genome (top) and the read (bottom). Connected bars represent seeds found between the read and the reference genome. Although it is collinear with the other seeds, the pair of connected red lines is not used as part of the chain because it does not meet the skew condition. Note that inclusion of the red seed in the chain would have led to a sub-optimal alignment, as is indicated by the location of the dark grey

areas.

The maximal amount of skew allowed between two consecutive seeds in a chain is dependent on the outer distance between the matching substrings and the maximal allowed percentage of differences in a feasible alignment, which is set by the parameter `-e`. The value of this parameter can be increased if large insertions and deletions are expected to be present in the chain. If optimal alignments would contain long gaps, the algorithm will produce much shorter chains, resulting in a higher dynamic programming cost. It will, however, still be able to retrieve the alignment.

Number of chains per candidate region

Because Algorithm 6 depends on a good choice for the anchor seed, it is likely that the first chain does not result in an optimal alignment within that region. This is the case when candidate regions contain many seeds, which often occurs in repetitive regions of the reference genome or in the cases where candidate regions were merged during identification. ALFALFA therefore constructs multiple chains per candidate region.

Initially, ALFALFA considers the entire list of seeds, ordered by length, as possible anchors for a chain. After each call of Algorithm 6, seeds that were used in the chain are removed from the list of potential anchors. In most cases, we have found that this procedure eliminates most seeds as anchors after the first two chains have been constructed. Moreover, the remaining anchors usually cannot be combined with other seeds due to a high skew. ALFALFA will not consider these single-seed chains if their coverage of the read is below the same threshold set for candidate regions.

A situation in which constructing multiple chains is mandatory is depicted in Figure S20, which uses the same candidate region representation as before. Seeds depicted in black are the only seeds that are part of an optimal alignment, whereas seeds depicted in red lead to suboptimal alignments. Furthermore, there is a tie for the choice of the initial anchor (longest seed), which shows that the longest seed does not always lead to the best possible chain.

Alignment

The chains obtained by Algorithm 6 represent gapped alignments of the read against the reference genome, not containing the unaligned sections between each pair of consecutive seeds and possibly at the beginning and end of the read. Algorithm 7 describes how chains are extended to full alignments that also cover the previously unaligned sections.

Before starting the alignment procedure, Algorithm 7 first checks whether the chain meets the same requirements imposed on candidate regions. Similar to the coverage requirements for candidate regions, single-seed chains that cover few bases of the read are omitted from alignment.

If the chain meets the minimum coverage requirements, the algorithm proceeds by filling the gaps in the alignment between each pair of consecutive seeds. For normal gaps, we use a standard banded dynamic programming routine. In some cases, however, dynamic programming can be avoided. For example, if the gap in both reference genome and read is a single base, the algorithm just inserts a single mutation. Likewise, if there is a positive distance between two

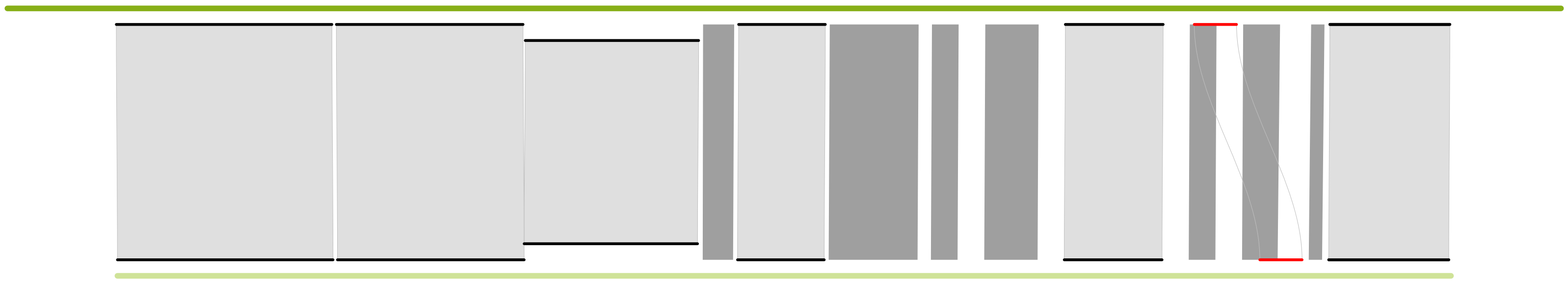

Figure S19: Example motivating the limitation of *skew* in between two seeds in a chain. Grey areas in the figure represent sequences of more than three matches in an optimal alignment between the reference genome (top) and read (bottom). Connected bars represent seeds found between read and reference genome. Black lines correspond to seeds in the chain. Red lines are not included in the chain.

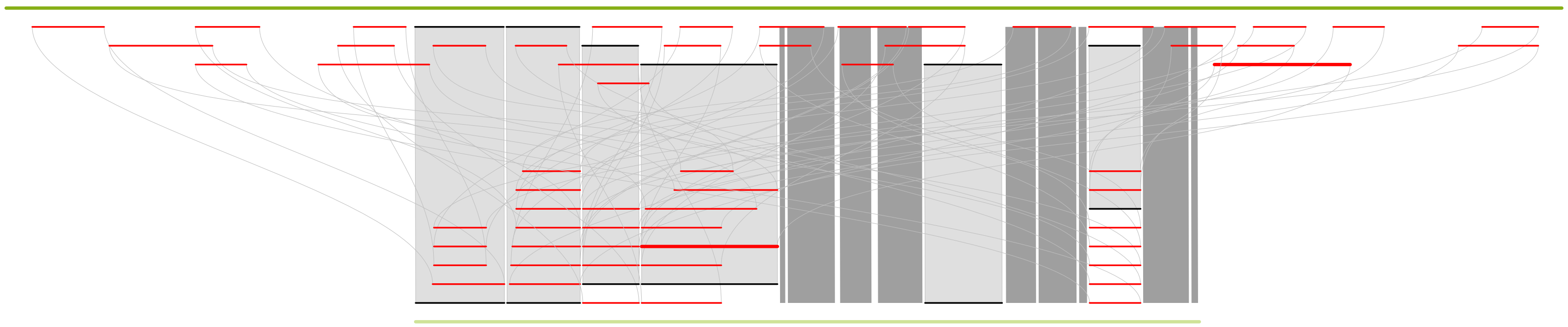

Figure S20: Example motivating the use of multiple chains per candidate region. Connected black and red lines depict seeds between a candidate region in the reference genome (top) and the read (bottom). Only the seeds depicted in black are part of an optimal alignment, of which the matches are shown by the grey areas. The large amount of seeds makes finding a good chain challenging. This can be alleviated by the construction of multiple chains.

Algorithm 7 Extend chain to alignment

Input: reference genome G, read R, collinear *chain* of seeds between G and R
Output: Alignment between R and the region of G bounded by *chain*

```
c ← minimum coverage set by parameter -c
if chain consists of 1 seed and coverage of R < c then
    return
end if
p ← pop first seed from chain
add p matches to alignment
for all seeds s in chain do
    calculate gap between p and s
    if gap can be filled with single mutation then
        add mutation to alignment
    else if gap can be filled with single (long) indel then
        add insertion or deletion of length gap to alignment
    else
        perform banded alignment in gap
    end if
    add s to alignment
    p ← s
end for
if first seed of chain did not start at first base of R then
    perform semi-global alignment between first seed and start of R
    l ← local alignment score
    g ← global alignment score
    if acceptLocal and g < l + 3 mutation differences then
        use local alignment
    else
        use global alignment
    end if
end if
if last seed of chain did not end at last base of R then
    ... // same procedure as above
end if
if alignment score ≤ minscore and no previous alignment found within
    minDistance bases then
    add alignment to R
end if
```

seeds in one sequence, but no gap in the other, the algorithm adds an insertion or deletion of appropriate size. Finally, banded semi-global alignment is performed in the region between the ends of the read and the ends of the chain.

For dynamic programming, we use an affine gap model with default match bonus of 1, mismatch penalty of 4, gap open penalty of 6 and gap extension penalty of 1. The band size is estimated using the maximum allowed percentage of differences in a feasible alignment, which is set by parameter `-e`. This value is decreased after each dynamic programming routine to more correctly represent the current situation after several differences have already been added.

The implementation of the dynamic programming algorithms was obtained from the *klib*[3] library. We made some minor changes to the library, in addition to the changes made in BWA-MEM, to facilitate global and semi-global alignments. These changes allow the semi-global alignment algorithm to report both local and global alignment scores. Similar to BWA-MEM, ALFALFA uses both scores to automatically decide between both alignment types. Local alignment is only preferred if the parameter `--local` is set and the global alignment score is smaller than the adjusted local alignment score for which three mutations were added.

The obtained alignment score is compared to a minimum score to decide whether the alignment is considered feasible. The minimum alignment score is obtained by starting from the maximum obtainable score and substracting the maximum allowed number of differences e multiplied by the mismatch penalty. The maximum number of differences is given by multiplying the value of parameter `-e` with the read length. In addition to being feasible, the alignment should also be e bases separated from another feasible alignment with a higher alignment score.

The use of chain-guided alignment can significantly lower the computational cost of the extension phase of the algorithm. An illustration of the possible magnitude of this effect is given in Figure S21. The green band around the trace shows the fraction of the matrix values that need to be computed in standard banded dynamic programming, whereas the grey areas show the fraction of matrix values that are computed in chain-guided alignment. The band width of both methods was calculated using the same variables, but the band width of the chain-guided alignment decreased as more differences are inserted during the procedure.

In practice, we have found that chain-guided alignment is up to twice as fast as alignments using standard banded dynamic programming. Moreover, chain-guided alignment does not seem to lead to a significant drop in accuracy, thanks to the conservative collinear chaining algorithm.

Alignment post-processing

During the alignment process, ALFALFA stores all alignments whose alignment score is higher than a minimum value. The number of feasible alignments is usually much higher than the requested number of alignments a as set by parameter `-a`. In a final post-processing step (line 28), ALFALFA selects the a highest scoring alignments and computes mapping qualities and CIGAR-strings for these alignments.

[3] https://github.com/attractivechaos/klib

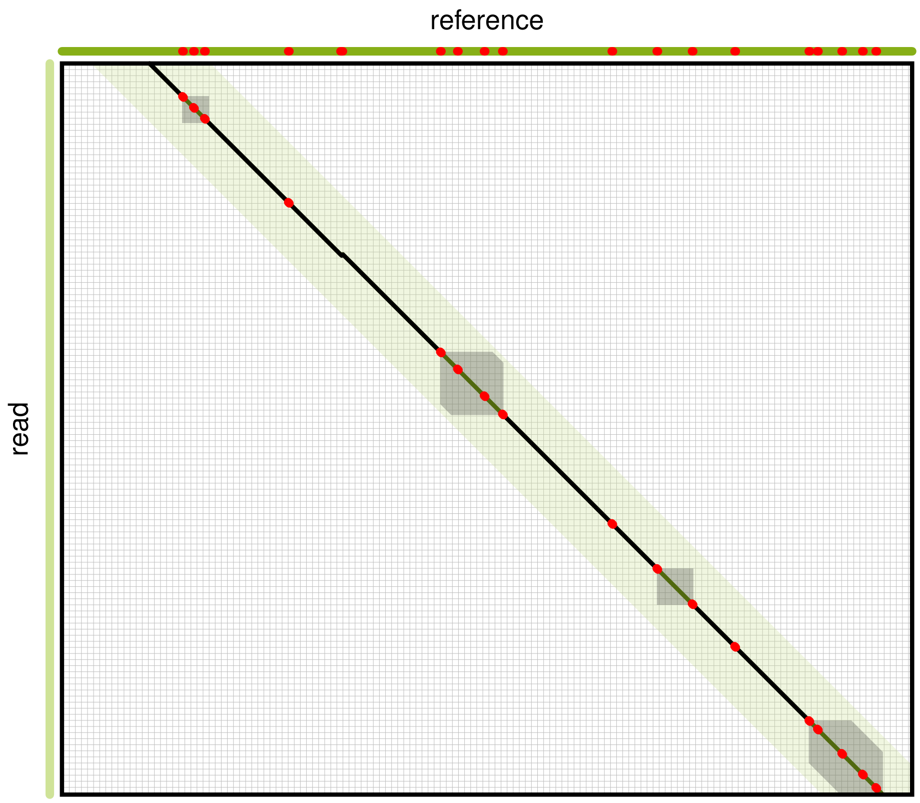

Figure S21: A possible alignment situation to illustrate the magnitude of possible savings in computational cost by using a chain-guided alignment in comparison to standard banded dynamic programming. The figure shows the dynamic programming matrix for the alignment of a reference genome (rows) and read (columns). The piecewise linear line is the trace of an optimal alignment. Black parts of this line indicate locations of seeds part of the chain, red dots indicate mismatches in the alignment and green lines were not covered by seeds. The band size for banded dynamic programming is indicated in green and the areas in grey indicate the areas in which dynamic programming is performed for a chain-guided alignment.

To obtain the CIGAR-string for an alignment, ALFALFA needs to perform another extension of the chain/candidate region that led to the alignment. This is because the dynamic programming routines in Algorithm 7 do not trace back the alignment after finding the alignment score. By default, the algorithm will again perform the chain-guided alignment, but users can also set the `--full-dp` parameter to use a banded dynamic programming routine over the full length of the read instead. This option might improve the quality of the alignment as seed positions may be wrong sometimes. In our benchmarks, enabling this option caused the runtime to increase on average by 70%. For long reads, this means that ALFALFA is still twice as fast as other mappers. The gain in accuracy and recall rate was small, but we did find an improvement in the edit distance and alignment score for a number of reads. In the near future, the option will be used automatically depending on the result of the previous alignment phases.

Mapping quality is estimated by a function that is widely used among read mappers. If s_1 is the best alignment score of the best alignment and s_2 is the alignment score of the second best alignment, the mapping quality for the best alignment is given by

$$250 \times \frac{s_1 - s_2}{s_1}.$$

This means that the mapping quality is zero if the two best alignments have an equal alignment score. In addition, mapping quality uses the sum of the alignment scores of concordantly mapped reads when mapping paired-end reads. For ease of comparison, we also set the maximum attainable mapping quality to 60, which is the same as used by BWA-MEM.

Paired-end read mapping

Similar to single-end read mapping, ALFALFA is designed to report a maximum number (parameter `-a`) of different *feasible* (parameter `-e`) paired-end alignments between reads and reference genomes. The added requirement is that pairs need to be aligned *concordantly* with respect to a preset orientation and insert size. Parameter settings for mapping paired-end reads have been adopted from Bowtie 2 and include options to (dis)allow mates to overlap, contain or dovetail one another and options to (dis)allow discordant or unpaired alignments. A graphical representation of the six different paired-end mapping algorithms implemented in ALFALFA can be found in Figure S22.

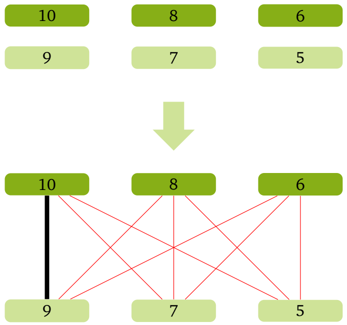

paired-end method 1

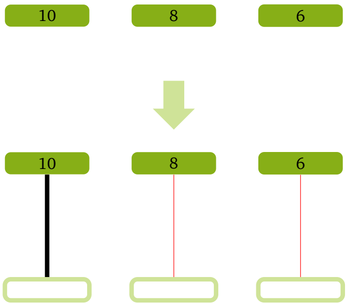

paired-end method 2

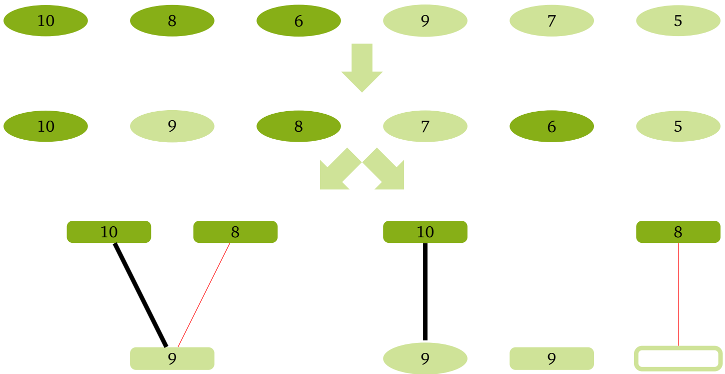

paired-end method 3

paired-end method 4

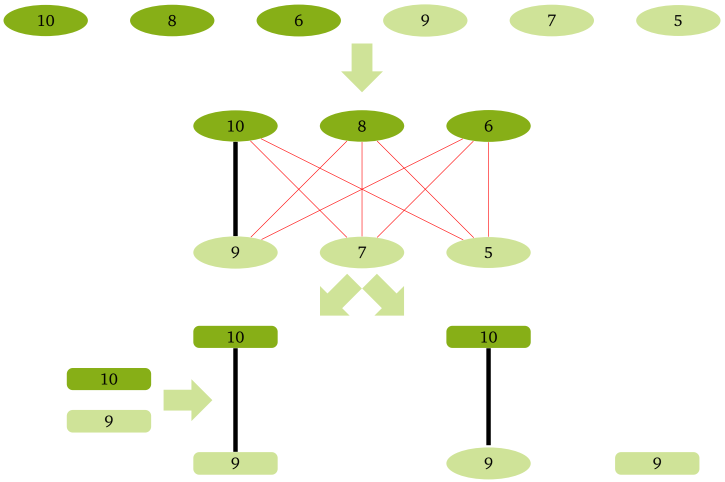

paired-end method 5

paired-end method 6

Figure S22: *(previous page)* Graphical representation of the various methods for paired-end alignment implemented by ALFALFA. The schematic represents alignments as colored rectangles, candidate regions as ellipses and other regions in the reference genome (containing no seeds) as empty rectangles. Dark green is used for mate 1 and light green for mate 2. Numbers within shapes represent scores used to sort candidate regions and alignments, i.e. alignment scores or coverage of bases in the read. Lines between shapes indicate pairing between structures to check orientation and insert size. Structures that can be paired are indicated by thick black lines, whereas failed comparisons are shown in red.

Algorithm 8 Default paired-end read mapping algorithm

Input: reference genome G, list of paired-end reads L_R
Output: SAM file containing alignments of reads onto G

read input and parse parameter options
construct the enhanced sparse suffix array I for G
for all $mate_1$, $mate_2$ in R **do**
    calculate alignments for $mate_1$
    calculate alignments for $mate_2$
    for all pairs of alignments (a_1,a_2) of respectively $mate_1$ and $mate_2$ **do**
        if orientation and insert size between a_1 and a_2 are correct **then**
            report concordant alignment pair (a_1,a_2)
        end if
    end for
    if not enough concordant pairs found **then**
        RESCUE($mate_1$,$mate_2$) *// try other paired-end alignment method*
    end if
    if report discordant or unpaired alignments **then**
        report all discordant and unpaired alignments found
    end ifPOSTPROCESS($mate_1$,$mate_2$)
end for

The first approach we tested is widely used among read mappers. The algorithm independently maps both mates of the pair and checks concordancy for every combination of feasible alignments found for the two mates. This method is also described in Algorithm 8, as it is currently the default paired-end mapping algorithm.

The second method for paired-end mapping is also used frequently among read mappers. For this method, a single mate is mapped, after which alignments for the second mate are found using dynamic programming in a window defined by the concordancy constraints (orientation and insert size). If the alignments of the first mate did not result in concordant alignments, the second mate is aligned and the process of finding a concordant alignment is repeated.

For the next methods, we tried to limit the number of times the single-end extension phase is called, as we found this to be the most costly subroutine in the mapping algorithm. This is done by using information from the candidate regions of both mates. In the third and fourth methods, we first calculated candidate regions for both mates. We subsequently merged the lists of candidate

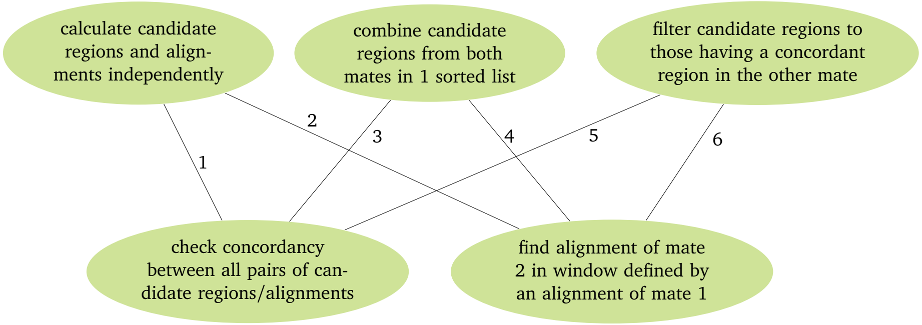

Figure S23: Schematic showing the relationship between all implemented methods for paired-end mapping. The top diagrams represent the different ways candidate regions are used. The bottom diagrams represent the two methods for finding concordant alignments, i.e. pairing two existing alignments or calculating an alignment for one mate given an alignment of the other.

regions into a single list, which is then sorted by coverage of the reads. This procedure effectively prioritizes extension of the best candidate regions among those of both mates.

Using the union of candidate regions from both mates, method 3 behaves like a single-end mapping algorithm and reports alignments for both mates. The alignments reported by the algorithm are then compared to find concordant pairs. In contrast to method 3, method 4 does not postpone the pairing of alignments until all candidate regions have been processed. Immediately after finding a feasible alignment, method 4 searches for a concordant alignment of the other mate in a window defined by the concordancy restraints. In contrast to method 2, however, this method can make use of a chain-guided alignment using the candidate regions for the other mate.

The last two methods also use candidate region information to speed up alignment of paired-end reads. Instead of merging the lists of candidate regions of both mates, however, methods 5 and 6 compare all pairs of candidate regions for the concordancy constraints and filter those candidate regions that do not pair with any other region of the other mate. Using the filtered list of candidate regions, method 5 then behaves exactly like methods 1 and 3, whereas method 6 uses the same strategy employed by method 4. A recap of the relationship between all methods for paired-end alignment can be found in Figure S23.

If the algorithms did not find enough concordant alignments, rescue procedures are used if the command line option `--paired-rescue` was set. These rescue procedures will call method 1, except for method 1 itself, which calls method 2. ALFALFA will also combine the best alignments for both mates into discordant pairs or select unpaired alignments, if required by the user. Finally, the post-processing procedure calculates mapping quality and CIGAR-strings for the alignments that will be reported.

Tests have shown that all methods have similar performance in both time and accuracy. Overall, we chose method 1 as the default, because of its simple design and stable performance over all data sets. In comparison to the first method, the second is somewhat more accurate in some situations, but it is not well suited for very long reads or for data sets with a broad variation in insert

sizes. In contrast, methods 5 and 6 were somewhat faster than method 1 due to the shorter list of candidate regions. However, the lower runtime comes at the cost of a drop in accuracy. The trade-off obtained by methods 3 and 4 is almost identical and lies in between that of method 1 on the one hand and methods 5 and 6 on the other hand. Further testing might reveal more information on which methods perform best for certain data sets.

Supplementary Protocol

This section provides details on our benchmark study to compare several state-of-the-art long read mappers, including a description of the read simulation process and parameters settings used for tuning the read mappers. All tests were run on a cluster with dual-socket quad-core Intel Xeon Nehalem (L5520) processors at clock speed 2.27GHz and 12GB RAM/node running Scientific Linux 6.5. The gcc v4.4.7 compiler was used to build all read mappers from source code, except for GEM which is not open source. The human genome assembly GRCh37, obtained from the UCSC genome browser website, was used as a reference genome for both simulation and mapping processes.

1 Data sets

ALFALFA achieves high performance in mapping long sequencing reads that are expected to become commonplace in the near future. As mapping of this type of read data has not yet been benchmarked and sequencing platforms show various error rates, we examined a broad range of different read lengths, error models and error rates using two existing read simulators.

The wgsim package is not designed to simulate reads for particular sequencing technologies, but allows to specify a uniform error rate and a relative fraction of indels over mutations in generating simulated reads. In order to evaluate the robustness of read mappers against different error models, we explored error rates ranging from 2% to 10% with either low (10%) or high (90%) indel rates.

The Mason read simulator generates reads with errors modeled after the ones produced by Illumina and 454 sequencing technologies. Although these technologies do not (yet) produce reads having lengths as the ones covered in our benchmark study, future sequencing technologies attaining such read lengths could produce errors resembling those of current technologies. The Mason simulated read sets also test the robustness of mappers against different error models.

To prove the competitiveness of ALFALFA on current moderately sized sequencing reads, artificial data sets were simulated using Mason to resemble reads generated by current sequencing platforms. This was done using the same parameter settings as used in the Bowtie 2 and RazerS3 benchmarks [12, 13]. We also used a read data set of shorter reads, simulted using wgsim. In addition, we also included several real read sets in the benchmark study. Note that simulated data allow for an evaluation of the accuracy of read mappers in addition to measuring their speed of execution, whereas real data sets only allow for evaluation of performance.

1.1 Real reads

The real Illumina data set included in the benchmark study consists of sequencing run ERR024139 of the ERX009608 experiment (EBI Short Read Archive). It contains over 26 million paired-end 100bp reads with an average insert size of 300bp. This data set was also used in the benchmark study of CUSHAW2 [3]. The 454 read set that is part of our benchmark has NCBI Short Read Archive accession number SRR003161 and consists of approximately 1.3 million reads

with an average length of 574bp. This data set is also part of the benchmark undertaken for several other mappers [12, 14, 15].

1.2 Wgsim simulated reads

A total of 18 read sets of 200000 reads were simulated with wgsim v0.3.1-r13 [16] using all possible combinations of 3 read lengths, 3 error rates and 2 indel rates. To simulate different indel models, both the mutation rate and indel fraction were set, but base error rate was set to zero. The following command line and arguments were used to generate the read sets:

```
wgsim -e 0.00 -N 200000 -1 <read length> -r <error rate>
    -R <indel rate> <reference.fa> <reads.fq>
```

- read length = $\{1000, 5000, 10000\}$
- error rate = $\{0.02, 0.05, 0.1\}$
- indel rate = $\{0.1, 0.9\}$

A final read set of 600bp single-end reads was used to compare the specificity of read mappers and was obtained using the following command line:

```
wgsim -h -R 0.3 -1 600 -N 500000 -r 0.01 -S 11 <reference.fa>
    <reads.fq>
```

1.3 Mason simulated reads

Read sets with Illumina and 454 error models were simulated with Mason v0.1.1 using either default parameters (100bp and 200bp data sets), parameters used in Bowtie 2 [12] (longer 454 data sets) or RazerS3 [13] (longer Illumina data sets). Due to the long execution time and memory requirements of the Mason read simulator, some data sets were generated in parts (using different seeds for the random generator) and concatenated afterwards. The following command line arguments were used:

1 million 2×100bp and 2×200bp read pairs following the Illumina error model.

```
mason illumina -N 1000000 -o <output_name> -sq -ll 300 -le 30
    -mp -rn 1 -n 100 <reference.fa>
mason illumina -N 1000000 -o <output_name> -sq -ll 3000 -le 300
    -mp -rn 1 -n 200 <reference.fa>
```

1 million 600bp and 800bp reads and 2×600bp and 2×800bp read pairs following Illumina and 454 error models. The simulation was performed in two parts of each 0.5 million reads with seeds 0 and 2000000.

```
mason illumina -N 500000 -o <output_name> -s <seed> -sq -hs 0.006
    -hM 32 -n 600 -pi 0.0001 -pd 0.0001 <reference.fa>
mason illumina -N 500000 -o <output_name> -s <seed> -sq -hs 0.006
    -hM 32 -n 800 -pi 0.0001 -pd 0.0001 <reference.fa>
mason illumina -N 500000 -o <output_name> -s <seed> -sq -ll 3000
    -le 300 -mp -rn 1 -hs 0.006 -hM 32 -n 600 -pi 0.0001
    -pd 0.0001 <reference.fa>
mason illumina -N 500000 -o <output_name> -s <seed> -sq -ll 3000
    -le 300 -mp -rn 1 -hs 0.006 -hM 32 -n 800 -pi 0.0001
    -pd 0.0001 <reference.fa>
```

```
mason 454 -N 500000 -o <output_name> -s <seed> -sq -hn 2 -nm 600
    -ne 60 -k 0.3 -bm 0.4 -bs 0.2 <reference.fa>
mason 454 -N 500000 -o <output_name> -s <seed> -sq -hn 2 -nm 800
    -ne 80 -k 0.3 -bm 0.4 -bs 0.2 <reference.fa>
mason 454 -N 500000 -o <output_name> -s <seed> -sq -ll 3000
    -le 300 -mp -rn 1 -hn 2 -nm 600 -ne 60 -k 0.3 -bm 0.4 -bs 0.2
    <reference.fa>
mason 454 -N 500000 -o <output_name> -s <seed> -sq -ll 3000
    -le 300 -mp -rn 1 -hn 2 -nm 800 -ne 80 -k 0.3 -bm 0.4 -bs 0.2
    <reference.fa>
```

100000 1kbp reads following Illumina and 454 error models.

```
mason illumina -N 100000 -o <output_name> -sq -hs 0.006 -hM 32
    -n 1000 -pi 0.0001 -pd 0.0001 <reference.fa>
mason 454 -N 100000 -o <output_name> -sq -hn 2 -nm 1000 -ne 100
    -k 0.3 -bm 0.4 -bs 0.2 <reference.fa>
```

50000 5kbp reads following Illumina and 454 error models. The simulation was performed in five parts of each 10000 reads by increasing the seed by 5 million for each part, starting from zero.

```
mason illumina -N 10000 -o <output_name> -s <seed> -sq -hs 0.006
    -hM 32 -n 5000 -pi 0.0001 -pd 0.0001 <reference.fa>
mason 454 -N 10000 -o <output_name> -s <seed> -sq -hn 2 -nm 5000
    -ne 500 -k 0.3 -bm 0.4 -bs 0.2 <reference.fa>
```

10000 10kbp reads following Illumina and 454 error models. The simulation was performed in ten parts of each 1000 reads by increasing the seed by 10 million for each part, starting from zero.

```
mason illumina -N 1000 -o <output_name> -s <seed> -sq -hs 0.006
    -hM 32 -n 10000 -pi 0.0001 -pd 0.0001 <reference.fa>
mason 454 -N 1000 -o <output_name> -s <seed> -sq -hn 2 -nm 10000
    -ne 1000 -k 0.3 -bm 0.4 -bs 0.2 <reference.fa>
```

2 Performance and accuracy measurements

Wall times of test runs were measured using the GNU/Linux `time` command. Index construction time was not taken into account, but can be found in Table 1. Peak memory measurements in Table 1 were obtained using the Python script `ps_mem.py` [17]. Tools for measuring recall rate and accuracy are custom developed for this benchmark study and are available using the ALFALFA `evaluate` command. The true positive rate and false positive rate were obtained using the `wgsim_eval.pl` script, which is part of the wgsim tool.

For the *accuracy measure*, a read is considered to be mapped correctly if at least one of the reported alignments maps within 10bp of the original simulated position, or if it has at least one alignment with an edit distance that is not higher than that of the simulated read. The *recall rate* only takes the distance to the simulated origin into consideration. Quality values reported by read mappers were used for the ROC curves in Figures S2 to S7.

The commands that were used to evaluate accuracy using the ALFALFA `evaluate` command require all input files to be sorted by read name, which can be done using SAMtools [18].

For the real read data sets, ALFALFA `evaluate summary` is used to provide information on the number of mapped reads for which the actual mapping locations are unknown.

```
alfalfa evaluate summary [--paired] -i <results.sam> -o <output>
    --reads <number of reads in the data set>
```

ALFALFA `evaluate sam` is used to evaluate the accuracy for reads simulated by Mason. It requires a SAM file containing the original mapping positions as produced by the Mason simulator. The reference genome is required to compute the edit distance from the CIGAR-string.

```
alfalfa evaluate sam -r <reference.fa> -i <results.sam>
    -o <output> --reference-sam <reference sam.sam>
```

ALFALFA `evaluate wgsim` is used to evaluate the accuracy for reads simulated by wgsim. Because wgsim stores information about the original location and the number of differences in the name of a read, no additional SAM file is required. The reference genome is required to compute the edit distance from the CIGAR-string.

```
alfalfa evaluate wgsim -r <reference.fa> -i <results.sam>
    -o <output>
```

3 Read mappers

Many long read mappers have been developed over the last couple of years. As it is practically infeasible to evaluate all read mappers in a single benchmark study, we limited ourselves to five state-of-the-art read mappers whose properties resemble those of ALFALFA, namely Bowtie 2, BWA-MEM, BWA-SW, CUSHAW3 and GEM. All these mappers are reported to have a high performance in mapping next generation sequencing data, including currently available long reads, but are not specifically designed for a single sequencing technology. They are accurate in the presence of many indels, have a low memory footprint and are freely available. If possible, the tested read mappers were built from source code and their index structures were generated locally. Read mappers were configured with default parameter settings, unless their authors suggested specific settings for certain types of data.

3.1 ALFALFA (v0.8) commands

The sparseness of the index structure has to be specified during index construction. The results in this paper use sparseness 12, unless stated otherwise. We also explored the effect of the sparseness in Figure S9. Paramater `-a` was used the set the maximum number of reported alignments. Results in Figure 4 were obtained using `-a 1`. Table $S1$ also contains results for `-a 4`. For the wgsim data sets, edit distance was specified as the percentage of differences that were generated using the parameter `-e`. For all remaining data sets, the default edit distance was used. Global alignment was specified for data sets with read length $<$ 500bp, whereas local alignment was specified for longer reads.

Index construction:

```
alfalfa index -r <reference.fa> -p <index_prefix> -s <sparseness>
```

Single-end reads:

```
alfalfa align -i <index_prefix> -0 <reads.fq> -o <output.sam>
     -e <max edit distance> -a <alignment count> [--local]
```

Paired-end reads:

```
alfalfa align -i <index_prefix> -1 <mate1.fq> -2 <mate2.fq> -I
    <min_insert_size> -X <max_insert_size> -o <output.sam> -e <max
    edit distance> -a <alignment count> [--local]
```

3.2 BWA-MEM (v0.7.9a) commands

Both BWA-MEM and BWA-SW use the same index construction command from the BWA-suite. The parameter `-a` was used to report multiple alignments and the parameter `-I` was used to set the mean insert size. The default insert size standard deviation was used.

Index construction:

```
bwa index -p <index_prefix> <reference.fa>
```

Single-end reads:

```
bwa mem [-a] <index_prefix> <reads.fq> > <output.sam>
```

Paired-end reads:

```
bwa mem [-a] -I <mean_insert_size> <index_prefix> <mate1.fq>
    <mate2.fq> > <output.sam>
```

3.3 BWA-SW (v0.7.9a) commands

Both BWA-MEM and BWA-SW use the same index construction command from the BWA-suite. BWA-SW does not allow the user to specify a maximum percentage of errors nor does it allow to specify bounds on the insert size.

Index construction:

```
bwa index -p <index_prefix> <reference.fa>
```

Single-end reads:

```
bwa bwasw -f <output.sam> <index_prefix> <reads.fq>
```

Paired-end reads:

```
bwa bwasw -f <output.sam> <index_prefix> <mate1.fq> <mate2.fq>
```

3.4 Bowtie (v2-2.2.3) commands

We mainly used the default parameter settings of Bowtie 2. To report multiple alignments, we used parameter `-k 4`. For the results requiring a single alignment per read, we omitted parameter `-k`. Global alignment was specified for data sets with read length < 500bp, whereas local alignment was specified for longer reads. For paired-end reads, we also specified minimum and maximum insert size.

Index construction:

```
Bowtie 2-build <reference.fa> <index_prefix>
```

Single-end reads:

```
Bowtie 2-align -x <index_prefix> -U <reads.fq> -S <output.sam>
    [--bwa-sw-like] [-k 4]
```

Paired-end reads:

```
Bowtie 2-align -x <index_prefix> -1 <mate1.fq> -2 <mate2.fq>
    -S <output.sam> -I <min_insert_size> -X <max_insert_size>
    [--bwa-sw-like] [-k 4]
```

3.5 CUSHAW (v3.0.3) commands

We mainly used the default parameter settings of CUSHAW3, but used paramater `-multi` to specify the maximum number of alignments to be reported. We specified average insert size and standard deviation for paired-end reads.

Index construction:

```
cushaw3 index -p <index_prefix> <reference.fa>
```

Single-end reads:

```
cushaw3 align -r <index_prefix> -f <reads.fq> -o <output.sam>
    [-multi <alignments>]
```

Paired-end reads:

```
cushaw3 align -r <index_prefix> -q <mate1.fq> <mate2.fq> -o
    <output.sam> -avg_ins <avg_insert_size> -ins_std
    <std_insert_size> [-multi <alignments>]
```

3.6 GEM (build 1.376 beta) commands

A pre-built GEM index was downloaded from the GEM website as the indexer of this mapper ran into a fatal error on our test environment. By default, GEM does not output its results into SAM format but provides a tool to convert its output to SAM format. For fastQ input files, GEM requires setting the quality value offset. For real and simulated data generated by the Mason read simulator, we set this quality value to `offset-33`. Because wgsim does not produce meaningful quality values, we set the quality offset to `ignore` for data sets generated by this read simulator.
Parameter settings were mainly taken from the original GEM publication [15], including `--fast-mapping` option. We used parameter `-d` to set the maximum number of reported alignments per read. For paired-end reads, minimum and maximum insert size were set using the definition in the GEM paper. These parameter settings deviate from the definition used by other read mappers. For wgsim data, error percentages for the `-m` and `-e` options were set to the values used by the simulator (2%, 5% or 10%). For 454 data, we used `-m 0.08 -e 0.08`. For Illumina data we used `-m 0.06 -e 0.06`. Lower values were tried as well for Illumina data sets, but these resulted in a significant drop in accuracy.

Single-end reads:

```
gem-mapper -q <quality-offset> -m <error_percentage>
    -e <error_percentage> --fast-mapping -I <index_name>
    -i <reads.fq> -o <output_prefix>
gem-2-sam -q <quality-offset> --expect-single-end-reads
    -i <output_prefix.map> -o <output.sam>
```

Paired-end reads:

```
gem-mapper -q <quality-offset> -m <error_percentage>
    -e <error_percentage> --fast-mapping -p -E 0.30
    --min-insert-size <min_insert_size>
    --max-insert-size <max_insert_size> -I <index_name>
    -1 <mate1.fq> -2 <mate2.fq> -o <output_prefix>
gem-2-sam -q <quality-offset> --expect-paired-end-reads
    -i <output_prefix.map> -o <output.sam>
```

Supplementary Data

This section provides details on the command line options that can be used to tweak ALFALFA, default settings and general usage tips. The software package offers separate commands for index construction, read mapping and evaluating mapping accuracy. Index construction can also be combined with read mapping during a single run of the package. The `alfalfa` command has the following general anatomy

```
alfalfa <command> [<subcommand>] [options]
```

where `command` is either `index`, `align` or `evaluate`. Only the `evaluate` command requires an additional subcommand.

In what follows we describe the options associated with each of the commands. Options can have a single-letter (preceded by a single hyphen) or multi-letter (preceded by a double hyphen) name, or both. In the latter case, both names of the option can be used interchangeably. Each description of an option starts with its name or names (separated by a forward slash), followed by a tuple (between round brackets) indicating the data type and default value of the argument that has to be passed to the option. If no default value is given, it is mandatory to pass an argument to the option. Options for which no tuple is given are used as toggles to enable/disable certain features and need no extra argument.

1 Indexing a reference genome

The `index` command is used to construct the data structures for indexing a given reference genome. The constructed index is stored to disk over multiple files that are contained in the same directory and that have names sharing the same prefix. Index files contain bookkeeping information (extension `.aux`) and individual arrays of an enhanced sparse suffix array: reference genome (extension `.ref`), suffix array (extension `.sa`), longest common prefix array (extension `.lcp`), inverse suffix array (extension `.isa`; optional), child array (extension `.child`; optional) and 10-mer lookup array (extension `.kmer`; optional). The inverse suffix array is the only array that is not constructed by default. The child array and 10-mer lookup array are not strictly necessary and may be omitted in order to save memory, but at the cost of a drop in performance. The `index` command can be skipped as the `align` command also provides the option to generate an index and store it to disk. All options for customizing the `index` command can therefore also be used in combination with the `align` command.

1.1 Options

`-r/--reference` (`file`). Specifies the location of a file that contains the reference genome in multi-fasta format.

`-s/--sparseness` (`int`, 12). Specifies the sparseness of the index structure as a way to control part of the speed-memory trade-off.

`-p/--prefix` (`string`, filename passed to the `-r` option). Specifies the prefix that will be used to name all generated index files. The same prefix has to be passed to the `-i` option of the `align` command to load the index structure when mapping reads.

`--no-child`. By default, a sparse child array is constructed and stored in an index file with extension `.child`. The construction of this sparse child array is skipped when the `--no-child` option is set. This data structure speeds up seed-finding at the cost of (4/s) bytes per base in the reference genome. As the data structure provides a major speed-up, it is advised to have it constructed.

`--suflink`. Suffix link support is disabled by default. Suffix link support is enabled when the `--suflink` option is set, resulting in an index file with extension `.isa` to be generated. This data structure speeds up seed-finding at the cost of $4/s$ bytes per base. It is only useful when sparseness is less than four and minimum seed length is very low (< 10), because it conflicts with skipping suffixes in matching the read. In practice, this is rarely the case.

`--no-kmer`. By default, a 10-mer lookup table is constructed that contains the suffix array interval positions to depth 10 in the virtual suffix tree. It is stored in an index file with extension `.kmer` and requires only 8MB of memory. The construction of this lookup table is skipped when the `--no-kmer` option is set. The lookup table stores intervals for sequences of length 10 that only contain {`A`,`C`,`G`,`T`}. This data structure speeds up seed-finding if the minimum seed length is greater than 10.

`-h/--help`. Prints to standard error the version number, usage description and an overview of the options that can be used to customize the software package.

2 Mapping and aligning a read set

The `align` command is used for mapping and aligning a read set onto a reference genome. As this process can be customized through a long list of options, we have grouped them into several categories.

2.1 I/O options

`-r/--reference` (`file`, **part of the index**). Specifies the location of a file that contains the reference genome in multi-fastA format.

`-i/--index` (`string`). Specifies the prefix used to name all generated index files. If this option is not set explicitly, an index will be computed from the reference genome according to the settings of the options that also apply to the `index` command.

`--save`. Specifies that if an index is constructed by the `align` command itself, it will be stored to disk. This option is ignored if the index is loaded from disk (option `-i`).

`-0/--single` (`file`). Specifies the location of a file that contains single-end reads. Both fastA and fastQ formats are accepted. If both single-end and paired-end reads are specified, single-end reads are processed first.

`-1/--mates1` (`file`). Specifies the location of a file that contains the first mates of paired-end reads. Both fastA and fastQ formats are accepted.

`-2/--mates2` (`file`). Specifies the location of a file that contains the second mates of paired-end reads. Both fastA and fastQ formats are accepted.

`-o/--output` (`file`, filename passed to the `-r` option with additional `.sam` extension). Specifies the location of the generated SAM output file containing the results of read mapping and alignment.

2.2 Alignment options

`-a/--alignments` (`int`, `1`). Specifies the maximum number of alignments reported per read.

`--no-forward`. Do not compute alignments on the forward strand.

`--no-reverse`. Do not compute alignments on the reverse complement strand.

`-e/--edit-distance` (`float`, `0.08`). Represents the maximum percentage of differences allowed in accepting alignments and used in combination with the dynamic programming score function to calculate the minimum alignment score.

`--no-rescue`. Disables rescue procedures that are normally initiated when ALFALFA does not find seeds and/or alignments with the current parameters.

`-t/--threads` (`int`, `1`). Number of threads used during read mapping. Using more than one thread results in reporting read alignments in a different order compared to the order in which they are read from the input file(s).

2.3 Seed options

`--seed` (`MEM | SMEM | PSMEM`, `SMEM`). Specifies the type of seeds used for read mapping. Possible values are `MEM` for maximal exact matches, `SMEM` for super-maximal exact matches, and `PSMEM` for SMEMs with additional rare MEMs. The use of SMEMs generally boosts performance without having a negative impact on accuracy compared to the use of MEMs. On the other hand, there are usually many more MEMs than SMEMs, in general resulting in a higher number of candidate genomic regions. Reporting all MEMs might be useful if reporting more candidate mapping locations is preferred.

`-l/--min-length` (`int`, `auto`). Specifies the minimum seed length. This value must be greater than the sparseness value used to build the index (option `-s`). By default, the value of this option is computed automatically using the following procedure. A value of 40 is used for reads shorter than 1kbp. The value is incremented by 20 for every 500bp above 1kbp, with the total increment being divided by the maximum percentage of errors allowed in accepting alignments (option `-e`).

`-m/--max-seeds` (`int`, `10000`). Specifies the maximum number of same-length seeds that will be selected per offset in the read sequence. The value passed to this option is multiplied by the automatically computed skip factor that determines sparse matching of sampled suffixes from the read sequence. As a result, the actual number of seeds per starting position in the read might still vary. Higher values of this option result in higher numbers of seeds, increasing in turn the number of candidate genomic regions.

`--max-smems` (`int`, `10`). Specifies the maximum number of SMEMs per offset in the read sequence to allow MEM-finding. This only applies to PSMEM seeds.

--max-mems (int, 20). Specifies the maximum number of MEMs per offset in the read that can be used for candidate region identification.

--min-mem-length (int, 50). Specifies the minimum length MEMs need to have to be used for candidate region identification.

--no-sparseness. Disables the use of sparseness in the read sequence during seed-finding.

2.4 Extend options

--max-alignments (int, 5000). Specifies the maximum number of alignments calculated per read. This value should be higher than the number of reported alignments (option -a). Decreasing this value can increase performance of the algorithm, at the cost of a lower accuracy and worse mapping quality estimation.

-f/--max-failures (int, 10). Specifies the maximum number of successive candidate regions that are investigated without success before ALFALFA stops extending the candidate regions of a read. Extension can be restarted only if the remaining candidate regions contain unique seeds.

--reset-failures. If set, the counter of successive candidate regions that are investigated without success is reset if a feasible alignment is found. By default the counter is only reset if a new best alignment is found.

--skip-unique. By default, ALFALFA extends all candidate regions containing unique seeds. If this flag is set, the uniqueness criterion is not taken into account when deciding upon the extension of a candidate region.

-c/--min-coverage (float, 0.25). Specifies the minimum percentage of the read length that candidate regions containing a single seed need to cover before extension of the candidate region is taken into consideration.

--local. By default, ALFALFA uses global alignment during the last phase of the mapping process. Global alignment in essence is end-to-end alignment, as it entirely covers the read but only covers the reference genome in part. Local alignment is used during the last phase of the mapping process if the --local option is set, which may result in soft clipping of the read.

-b/--bandwidth (int, 100). Specifies the maximum bandwidth that is used by the banded alignment algorithm. The bandwidth used is automatically inferred from the specification of the maximum percentage of errors allowed in accepting alignments (option -e), but is bound by this parameter.

-M/--match (int, 1). Specifies the positive score assigned to matches in the dynamic programming extension phase.

-U/--mismatch (int, -4). Specifies the penalty assigned to mismatches in the dynamic programming extension phase.

-O/--gap-open (int, -6). Specifies the penalty O for opening a gap (insertion or deletion) in the dynamic programming extension phase. The total penalty for a gap of length ℓ equals $O + \ell \cdot E$. The use of affine gap penalties can be disabled by setting this value to zero.

-E/--gap-extend (int, -1). Specifies the penalty E for extending a gap (insertion or deletion) in the dynamic programming extension phase. The total penalty for a gap of length ℓ equals $O + \ell \cdot E$.

--full-dp. By default, ALFALFA uses chain-guided alignment to retrieve the CIGAR alignment. If this parameter is set, banded dynamic programming is performed instead. Activating this setting can greatly increase runtime, but can sometimes lead to more optimal alignments.

2.5 Paired-end mapping options

-I/--min-insert (int, 0). Specifies the minimum insert size.

-X/--max-insert (int, 1000). Specifies the maximum insert size.

--orientation (fr | rf | ff, fr). Specifies the orientation of mates. `fr` means a forward upstream first mate and reverse complemented downstream second mate or vice versa. `rf` means a reverse complemented upstream first mate and forward downstream second mate or vice versa. `ff` means a forward upstream first mate and forward downstream second mate or vice versa. Note that these definitions are literally taken over from Bowtie 2.

--no-mixed. Disables searching for unpaired alignments.

--no-discordant. Disables searching for discordant alignments.

--dovetail. Allows switching between upstream and downstream mates in the definition of their orientation (option `--orientation`).

--no-contain. Disallows concordant mates to be fully contained within each other.

--no-overlap. Disallows concordant mates to overlap each other.

--paired-mode (1 | 2 | 3 | 4 | 5 | 6, 1). Specifies the algorithm used to align paired-end reads. The possible algorithms are discussed in detail in the methods section. Algorithms `1` and `2` do not use information from candidate regions. Algorithms `3` and `4` prioritize extension of candidate regions over both reads. Algorithms `5` and `6` filter the list of candidate regions using the paired-end restraints. Algorithms with an odd number pair mapped reads after alignment. Algorithms with an even number perform dynamic programming across a window defined by the insert size restrictions to search for a bridging alignment reaching the other mate.

--paired-rescue. Enables a rescue procedue if no concordant alignment is found using the current parameter settings.

2.6 Miscellaneous options

-v/--verbose (int, 0). Turns on lots of progress reporting about the alignment process. Higher numbers give more verbose output. Information is printed to standard error and is useful for debugging purposes. The default value `0` disables progress reporting. The maximum verbosity level currently supported is `7`.

-h/--help. Prints to standard error the version number, usage description and an overview of the options that can be used to customize the software package.

3 Evaluating mapping accuracy

The `evaluate` command is used for evaluating the accuracy of simulated reads and summarizing statistics from the SAM formatted alignments reported by a read mapper. It requires an additional subcommand that influences both the functionality and the input of the `evaluate` command. Currently supported subcommands are `summary`, `sam` and `wgsim`.

The `evaluate` command requires all input files to be sorted by read name. This can easily be done using SAMtools. Furthermore, read names of both mates should be identical for paired-end reads.

3.1 Options shared by all subcommands

`-i/--input-sam` (`file`). Specifies the location of a SAM file that contains the read mapping alignments that need to be evaluated.

`-o/--output` (`file`, standard output). Specifies the location of the file that will contain the generated output.

`-q/--quality` (comma-separated list of `ints` between `0` and `255`, `0`). The values in the list represent quality thresholds. For each specified quality threshold, output is produced that reports only on the subset of alignments with quality value greater than or equal to the threshold.

`-p/--print`. Triggers the generated output to contain a list of all reads from the input SAM file followed by a binary value. Zero indicates that the read is either unmapped or incorrectly mapped and one indicates that the read was mapped (`summary` subcommand) or mapped correctly (other subcommands).

`-h/--help`. Prints to standard error the version number, usage description and an overview of the options that can be used to customize the software package.

3.2 Summary subcommand options

The `evaluate summary` subcommand reports statistics about the number of mapped reads for which the actual mapping locations are unknown.

`--reads` (`int`). Specifies the number of reads given as input to the read mapper that produced the input SAM file (option `--input-sam`). This number can be different from the number of reads contained in the input SAM file (option `--input-sam`) if for example unmapped reads are not reported.

`--paired`. By default, the input SAM file (option `--input-sam`) is supposed to contain single-end reads. If the `--paired` option is set, it is supposed to contain paired-end reads. The summary for paired-end reads contains information on the number of reads mapped as paired and unpaired, as indicated by the `flag` field of the SAM format.

3.3 Sam subcommand options

The `evaluate sam` subcommand is used to evaluate the accuracy for sequencing reads generated by the Mason simulator and other read simulators that produce a reference SAM file containing alignments for the simulated reads.

`-r/--reference` (`file`). Specifies the location of a file that contains the reference genome in multi-fasta format.

`-w/--window` **(comma-separated list of `ints`, `10`).** The values in the list represent window sizes around the position in the reference genome from which the simulated read was extracted. An alignment is considered to be mapped correctly if it is mapped within a given window around the simulated position. Output is generated for each individual value.

`--input-edit` (`string`, `NM`). Specifies the field of the SAM format that contains the edit distance of the alignments in the input SAM file (option `-i`). If no such field exists, the edit distance is computed from the CIGAR string, the read sequence and the reference genome. An alignment that is not mapped within a certain window around the simulated position (option `-w`) is considered accurately mapped if its edit distance is less than the edit distance of the alignment taken from the reference SAM file (option `--reference-sam`).

`--reference-sam` (`file`). Specifies the location of a reference SAM file containing alignments of the simulated reads as generated by the Mason simulator. Alignments contained in this file should be sorted by read name, which is easily done using SAMtools.

`--reference-edit` (`string`, `XE`). Specifies the field of the SAM format that contains the edit distance of the alignments in the reference SAM file (option `--reference-sam`). The default value is set to `XE` because this is the field used by the Mason simulator.

3.4 Wgsim subcommand options

The `evaluate wgsim` subcommand is used to evaluate the accuracy for reads simulated by wgsim.

`-r/--reference` (`file`). Specifies the location of a file that contains the reference genome in multi-fasta format.

`-w/--window` **(comma-separated list of `ints`, `10`).** The values in the list represent window sizes around the position in the reference genome from which the simulated read was extracted. An alignment is considered to be mapped correctly if it is mapped within a given window around the simulated position. Output is generated for each individual value.

`--input-edit` (`string`, `NM`). Specifies the field of the SAM format that contains the edit distance of the alignments in the input SAM file (option `-i`). If no such field exists, the edit distance is computed from the CIGAR string, the read sequence and the reference genome. An alignment that is not mapped within a certain window around the simulated position (option `-w`) is considered accurately mapped if its edit distance is less than the edit distance of the alignment taken from the reference SAM file (option `--reference-sam`).

References

[1] Vyverman M, De Baets B, Fack V, Dawyndt P: **essaMEM: finding maximal exact matches using enhanced sparse suffix arrays**. *Bioinformatics* 2013, **29**:802–804.

[2] Li H: **Aligning sequence reads, clone sequences and assembly contigs with BWA-MEM** [http://arxiv.org/abs/1303.3997].

[3] Liu Y, Schmidt B: **Long read alignment based on maximal exact match seeds**. *Bioinformatics* 2012, **28**:i318–i324.

[4] Liu Y, Popp B, Schmidt B: **CUSHAW3: sensitive and accurate base-space and color-space short-read alignment with hybrid seeding**. *PloS one* 2014, **9**:e86869.

[5] Li H: **Exploring single-sample SNP and INDEL calling with whole-genome de novo assembly**. *Bioinformatics* 2012, **28**:1838–1844.

[6] Gusfield D: *Algorithms on Strings, Trees and Sequences: Computer Science and Computational Biology*. New York: Cambridge University Press 1997.

[7] Manber U, Myers G: **Suffix arrays: a new method for on-line string searches**. *SIAM Journal on Computing* 1993, **22**:935–948.

[8] Abouelhoda MI, Kurtz S, Ohlebusch E: **Replacing suffix trees with enhanced suffix arrays**. *J Discrete Algorithms* 2004, **2**:53–86.

[9] Navarro G, Mäkinen V: **Compressed full-text indexes**. *ACM Comput Surveys* 2007, **39**:2:1–2:61.

[10] Vyverman M, De Baets B, Fack V, Dawyndt P: **Prospects and limitations of full-text index structures in genome analysis**. *Nucleic Acids Res* 2012, **40**:6993–7015.

[11] Kurtz S, Phillippy A, Delcher AL, Smoot M, Shumway M, Antonescu C, Salzberg SL: **Versatile and open software for comparing large genomes**. *Genome Biol* 2004, **5**:R12.

[12] Langmead B, Salzberg SL: **Fast gapped-read alignment with Bowtie 2**. *Nat Methods* 2012, **9**:357–359.

[13] Weese D, Holtgrewe M, Reinert K: **RazerS 3: Faster, fully sensitive read mapping**. *Bioinformatics* 2012, **28**:2592–2599.

[14] Li H, Durbin R: **Fast and accurate long-read alignment with Burrows-Wheeler transform**. *Bioinformatics* 2010, **26**:589–595.

[15] Marco-Sola S, Sammeth M, Guigó R, Ribeca P: **The GEM mapper: fast, accurate and versatile alignment by filtration**. *Nat Methods* 2012, **9**:1185–1188.

[16] Li H: **The wgsim read simulator** [https://github.com/lh3/wgsim].

[17] Brady P: **Memory measurement script** [http://www.pixelbeat.org/scripts/ps_mem.py].

[18] Li H, Handsaker B, Wysoker A, Fennell T, Ruan J, Homer N, Marth G, Abecasis G, Durbin R, 1000 Genome Project Data Processing Subgroup: **The Sequence Alignment/Map (SAM) format and SAMtools**. *Bioinformatics* 2009, **25**:2078–2079.
